# Inhibition of type II NADH dehydrogenase in *Mycobacterium tuberculosis* reveals collateral vulnerability in the electron transport chain

**DOI:** 10.1128/aac.00756-26

**Published:** 2026-09-24

**Authors:** Vien Q. T. Ho, Vincent Fontaine, Ernesto Anoz-Carbonell, Kamel Djaout, Clement Vigier, Leo Faion, Samsher Singh, Sushovan Dam, Jonathan Chatagnon, Stéphanie Slupek, Rudy Antoine, Aurélie Chauffour, Alexandra Aubry, Benoit Deprez, Kevin Pethe, Nicolas Veziris, Nicolas Willand, Robert Jansen, Baptiste Villemagne, Ruben C. Hartkoorn

**Affiliations:** 1 University Lille, CNRS, Inserm, CHU Lille, Institut Pasteur de Lille, U1019-UMR 9017, Center for Infection and Immunity of Lille (CIIL) 27023, Lille, France; 2 Centre d'Immunologie et des Maladies Infectieuses, CIMI, Sorbonne Université, CNRS, Inserm 27063, Paris, France; 3 University Lille, Inserm, Institut Pasteur de Lille, U1177-Drugs and Molecules for Living Systems 173548, Lille, France; 4 Lee Kong Chian School of Medicine, Nanyang Technological University 54761, Singapore, Singapore; 5 Centre National de Référence des Mycobactéries et de la Résistance des Mycobactéries aux Antituberculeux, AP-HP, Sorbonne Université 27063, Paris, France; 6 Singapore Centre for Environmental Life Sciences Engineering (SCELSE), Nanyang Technological University 54761, Singapore, Singapore; 7 National Center for Infectious Diseases (NCID), Singapore, Singapore; 8 Radboud Institute for Biological and Environmental Sciences, Radboud University 6029 , Nijmegen, the Netherlands; Queen Mary University of London, London, United Kingdom

**Keywords:** *Mycobacterium tuberculosis*, drug combination, respiration chain, NADH dehydrogenase, synergy

## Abstract

To sustain the anti-tuberculosis drug development pipeline, novel antibiotics must be developed both as standalone agents and as components of future combination regimens. To this end, tricyclic-spirolactams (TriSLa)-based inhibitors of mycobacterial type II NADH dehydrogenase (Ndh-2) represent a promising novel class of antibiotics, though activity is carbon source dependent. Initial studies established an enhanced understanding of the impact of TriSLa on mycobacteria using metabolomics and transcriptomics. Then, CRISPRi chemical-genetics and extensive *in vitro* TriSLa-combination studies showed that Ndh-2 inhibition resulted in rendering other components of the electron transport chain vulnerable, even in conditions when TriSLa was inactive alone. Finally, identified *in vitro* synergistic TriSLa-antibiotic combinations were validated to also give modestly improved *in vivo* efficacy. Together, this work expands our understanding of how Ndh-2 inhibition impacts bacterial physiology and reveals how it sensitizes the bacterium to electron transport chain inhibitors, providing a foundation for future TriSLa-based combination regimens.

## INTRODUCTION

Tuberculosis (TB), caused by *Mycobacterium tuberculosis* (*Mtb*), is the leading cause of death by an infectious disease and a major global public health threat (1). The current WHO-recommended treatment regimen for drug-susceptible TB was implemented in the 1970–1980s, a multidrug therapy incorporating the antibiotics isoniazid, rifampicin, ethambutol, and pyrazinamide (2). Increasing rates of multidrug-resistant (MDR) and extensively drug-resistant (XDR) tuberculosis are, however, progressively rendering this regimen non-efficacious, leaving the treatment regimen dependent on other second- and third-line antibiotics, which are less effective and more toxic. More recently, both the FDA and WHO have approved a novel BPaL/M-based regimen (containing bedaquiline, pretomanid, linezolid, and moxifloxacin) for the treatment of MDR and XDR-TB strains (3). While BPaL/M signifies a major advance in the treatment of tuberculosis, emerging resistance to both bedaquiline and pretomanid threatens the future effectiveness of this regimen (4–6). Consequently, there remains an urgent need to develop novel anti-*Mtb* compounds and to integrate them effectively into next-generation treatment regimens.

As a potent inhibitor of the ATP synthase, the discovery and development of bedaquiline (BDQ) as a cornerstone antibiotic for the treatment of MDR tuberculosis has paved the way for further developments of additional inhibitors of the bacterial electron transport chain (ETC) (7). An advanced example is telacebec (Q203), a potent antibiotic targeting the cytochrome bc1-aa3 oxidase, which is currently undergoing clinical development (phase II clinical trial was recently completed) (8, 9). Similarly, the type II NADH dehydrogenase (Ndh-2) of the ETC, for which there is no mitochondrial homolog, has gained considerable interest as a drug target. To date, several Ndh-2 inhibitors have been described, with mercapto-quinazolinones and tricyclic-spirolactams (TriSLa) being the most potent (10–14). Extensive optimization of TriSLa has led to the development of BDM91465, a low nanomolar inhibitor of Ndh-2 biochemical activity and *Mtb* growth, with improved drug metabolism and pharmacokinetic properties (15). Furthermore, BDM91465 was demonstrated to be efficacious in a murine model of *Mtb* infection (15), mimicking an infection by an Ndh-2 knock-out strain (16).

The *Mtb* ETC consists of three NADH dehydrogenases, namely a proton-pumping type I NADH dehydrogenase (Ndh-1, analogous to mitochondrial complex I) and two homologous type II NADH dehydrogenases (Ndh-2, designated Ndh and NdhA). Various studies have revealed considerable plasticity and functional redundancy between the *Mtb* NADH dehydrogenase systems, which depend on media carbon source, particularly fatty acids (FA) (16–18). Overall, at least one functional NADH dehydrogenase system is required for bacterial viability. Ndh-1 was found to be able to support bacterial growth in media lacking fatty acids where there was limited cellular NADH production, while the primary Ndh-2, Ndh, was required for growth in media containing fatty acids and where NADH production was greater (16). As a result, the Ndh-2 inhibitors, such as TriSLa, are conditionally active antibiotics, showing potent activity in fatty acid media, but relatively inactive in fatty acid-free media (12). Inactivation of Ndh-2 and full dependence on Ndh-1 prevents *Mtb* from sufficiently catalyzing the NADH oxidation for maintaining a normal NADH/NAD^+^ ratio and ATP production. Moreover, this state additionally leads to membrane hyperpolarization by excessive functioning of Ndh-1 (12, 16).

Considering the notable impact of *in vitro* media carbon source on NADH dehydrogenase essentiality and on the antibiotic activity of TriSLa, it was important to define their role *in vivo*. Ndh-1-deficient *Mtb* was found not to be attenuated for *in vivo* growth in mice, while infection with an Ndh-2-deficient *Mtb* strain produced an attenuated phenotype where bacteria remained capable of replication in murine lungs but at a reduced rate compared to the wild-type *Mtb* (16, 17). This attenuation mirrored the *in vivo* anti-*Mtb* efficacy of TriSLa (BDM91465) and is recapitulated *in vitro* when performing TriSLa activity assays on *Mtb* grown in fatty acid-free media. In addition, higher levels than the level of maximum dose of TriSLa did not improve efficacy, suggesting that BDM91465 monotherapy achieved full target inhibition *in vivo* (15).

While Ndh-2 is conditionally essential, we hypothesized that Ndh-2 inhibition by TriSLa in fatty acid-free media, or eventually *in vivo*, will nonetheless cause significant remodeling of bacterial metabolism and physiology in a manner that will alter their sensitivity to other antibiotics in combination. To test this hypothesis, we first characterized the impact of TriSLa exposure on *Mtb* under both fatty acid-rich and fatty acid-free conditions by transcriptomic and metabolomic analyses. We then employed a CRISPRi-based chemical-genetics approach to search for hypervulnerable targets in *Mtb* in the presence of TriSLa. Finally, we conducted extensive combination studies of TriSLa with other anti-*Mtb* agents to identify synergistic *in vitro* interactions, with subsequent validation *in vivo*. Collectively, this study advances our understanding of the effects of Ndh-2 inhibition on *Mtb* and highlights antibiotic combinations that synergize with TriSLa both *in vitro* and *in vivo*.

## RESULTS

### Dissecting the influence of single and multiple carbon sources on the anti-*Mtb* activity of TriSLa

Previous work showed that TriSLa’s anti-*Mtb* activity was dependent on FAs, such as oleic acid in culture media, which was consistent with *ndh-2* gene essentiality in fatty-acid-based media (12). To further dissect the contributions of different carbon sources in TriSLa activity, we evaluated activity in a minimal MMAT-based medium supplemented with tyloxapol and fatty acid-free BSA. In addition, we provided L-glutamine, an amino acid identified as the main nitrogen donor for intracellular *Mtb*, at a low concentration of 0.01%, reflecting its physiological plasma levels (19, 20). Single or combined carbon sources were then added to this minimal MMAT-based medium to determine their effect on TriSLa minimal inhibitory concentration (MIC).

**Fig 1:**
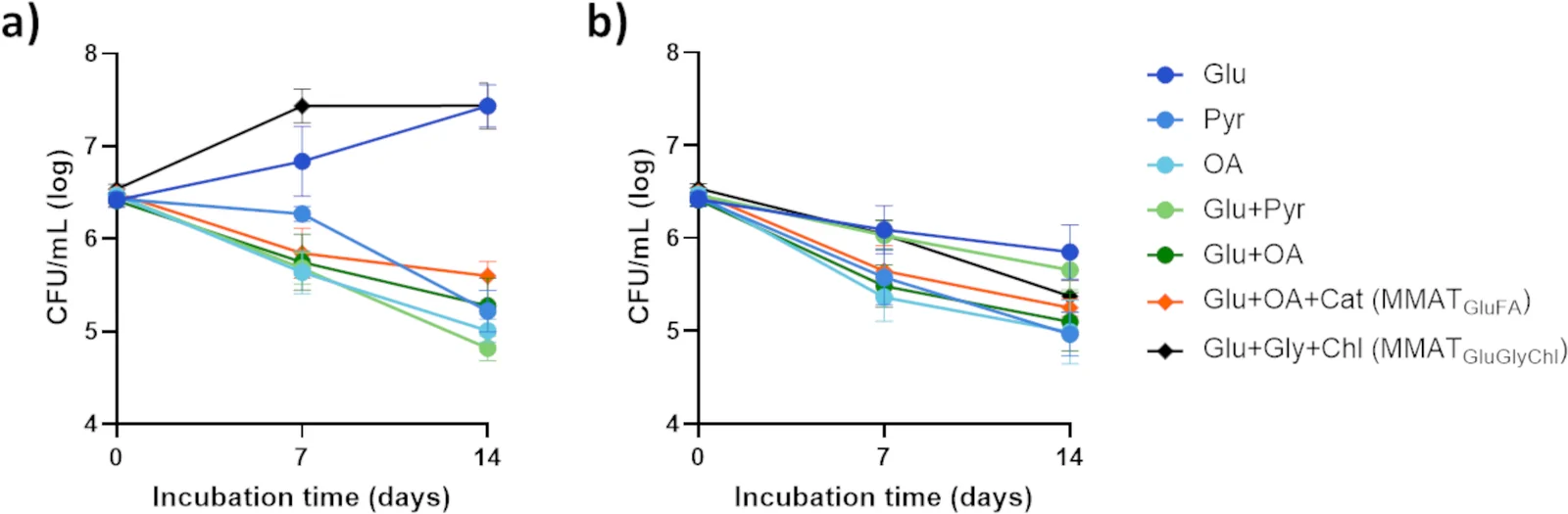
TriSLa anti-*Mtb* activity in MMAT-based media with supplemented carbon sources. Time-kill kinetics of (**a**) 0.4 µM BDM91465 and (**b**) 2 µg/mL BDQ on H37Rv growing in MMAT-based media supplemented with different carbon sources. Glu: 0.2% glucose, Pyr: 0.2% pyruvate, OA: 200 µM oleic acid, Chl: 0.02% cholesterol, Cat: 0.0003% catalase. Carbon sources for each condition were labeled as follows: Glu (dark blue circle), Pyr (blue circle), OA (light blue circle), Glu + Pyr (light green circle), Glu + OA (dark green circle), Glu + OA + Cat (MMAT_GluFA_) (orange diamond), Glu + Gly + Chl (MMAT_GluGlyChl_) (black diamond). All media were made with fatty acid-free BSA and 0.01% L-glutamine. Data are the mean and SD of three biological replicates.

Regarding the role of single carbon sources, *Mtb* grew optimally on pyruvate or fatty acids (oleic or palmitic acid), but more poorly on glycerol, glucose, acetate, propionate, or cholesterol (Table S1). BDM91465 was not active in MMAT-based media supplemented with glucose, glycerol, acetate, propionate, or cholesterol (MIC₉₀ > 10 µM), intermediately active in media with pyruvate (MIC₉₀ = 0.4 µM), and potently active in media with fatty acids (MIC = 0.03 µM for oleic acid and MIC₉₀ = 0.18 µM for palmitic acid) (Table S1). Addition of a second carbon source (glucose or glycerol) enhanced overall growth of bacteria while having little impact on the antibacterial activity of TriSLa, with the exception of MMAT supplementation with pyruvate and glucose or glycerol, where TriSLa activity was as observed in MMAT supplemented with fatty acid (Table S1). The unexpected activity of BDM91465 in MMAT supplemented with pyruvate and glucose or glycerol breaks the dogma that Ndh-2 vulnerability is uniquely dependent on fatty acids, and this may involve the impact of pyruvate on NADH/NAD^+^ ratios (see later section). During the analysis of carbon sources on TriSLa activity, bacteria were analyzed in parallel to BDQ, and its activity remained largely independent of carbon source (Table S2). In addition to MIC analysis, time-kill assays on H37Rv with 0.4 µM BDM91465 (10× MIC in fatty-acid media) or 2 µg/mL BDQ (3.6 µM, approximately 10× MIC) by measuring bacterial loads using colony-forming units (CFU) confirmed the role of carbon sources on TriSLa activity (Fig. 1).

### Selection of culture media for downstream experiments

TriSLa BDM91465 was recently found to mildly attenuate *Mtb* growth *in vivo* (15), and murine infections with an *ndh-2-*deficient *Mtb* mutant have previously been shown to be similarly attenuated (16). Both these studies showed that the vulnerability of Ndh-2 *in vivo* resembles the TriSLa susceptibility profile of *Mtb* when cultured in the non-fatty acid-containing media. Because of the important role of media in TriSLa activity, it was decided that all downstream assays investigating the impact of TriSLa and its role in combination studies needed to be performed in media where TriSLa was active and inactive. For the selection of media, we wanted to minimize growth-rate effects of different media and wanted to maintain a physiologically relevant concentration of glucose in both media, as it would allow for comparative metabolic downstream. As the “TriSLa-active” medium, MMAT_GluFA_ was selected, consisting of MMAT supplemented with 0.2% glucose (Glu), 200 µM oleic acid (FA), and 0.0003% catalase (which did not alter TriSLa activity—Table S1). As a “TriSLa-inactive” medium, MMAT_GluGlyChl_ was selected, consisting of MMAT supplemented with 0.2% glucose (Glu), 0.2% glycerol (Gly), and 0.02% cholesterol (Chl). We note that while cholesterol metabolism shares some features of fatty acid metabolism (e.g., β-oxidation-like side-chain degradation initiated by C26 hydroxylation), its inclusion in the media did not result in TriSLa activity, likely because its use as a carbon source leads to less NADH generation in these *in vitro* assays. Growth of *Mtb* (H37Rv-GFP) in MMAT_GluFA_ and MMAT_GluGlyChl_ was found to be comparable (Fig. S1a through c, measured by both OD_600_ and GFP), with the MIC of BDM91465 in MMAT_GluFA_ and MMAT_GluGlyChl_ being 0.022 µM and >10 µM, respectively (Table S1), which was further confirmed in time-kill assays (Fig. 1).

### Impact of media and TriSLa on bacterial ATP production, NADH/NAD^+^ ratio, and reactive oxygen species generation

While *Mtb* displayed a similar growth rate in MMAT_GluFA_ and MMAT_GluGlyChl_ (Fig. S1a through c), differences in the media carbon source are expected to impact central metabolism, likely changing the production of NADH (though NADH/NAD^+^ ratio can be expected to be maintained in homeostasis) and thus altering downstream bacterial ATP synthesis. To assess this impact, first the NADH/NAD^+^ ratios were measured on bacteria grown in both media in the absence and presence of 0.4 µM BDM91465 (10× MIC expected to inhibit Ndh-2 of *Mtb* in both media). For the DMSO control condition, the NADH/NAD^+^ ratio was found to be indifferent in both media (homeostasis maintained), while the addition of 0.4 µM BDM91465 significantly increased this NADH/NAD^+^ ratio in both media over 4 and 24 h in a similar manner, primarily by increasing relative NADH concentrations (Fig. S2). These data confirm the role of BDM91465 in changing the bacterial NADH/NAD^+^, but also show that in MMAT_GluGlyChl_, where the bacteria grow in the presence of TriSLa, the NADH/NAD^+^ ratio is still altered.

As an additional comparison to MMAT_GluFA_ and MMAT_GluGlyChl_, we also examined the NADH/NAD^+^ ratio of *Mtb* grown in MMAT supplemented with glucose and pyruvate (MMAT_GluPyr_) to evaluate if a change in redox state could help explain increased sensitivity to TriSLa. Surprisingly, bacteria grown in MMAT_GluPyr_ exhibited a substantially higher NADH/NAD^+^ ratio (Fig. S3a), primarily driven by a marked reduction in intracellular NAD^+^ levels (Fig. S3b). One possible explanation for the lower NAD^+^ concentrations is that increased pyruvate metabolism to acetyl-CoA enhances NAD^+^ consumption, thereby lowering the intracellular NAD^+^ pool; this, however, needs further investigation. In the presence of 0.4 µM BDM91465, this NADH/NAD^+^ ratio in *Mtb* grown in MMAT_GluPyr_ increased further and is consistent with the response seen in other media (Fig. S3d and e). Together, these findings may point to the altered basal NADH/NAD^+^ ratio being associated with TriSLa sensitivity of *Mtb* grown in MMAT_GluPyr_, but this needs to be further explored. This altered redox state may also explain previous reports of reduced isoniazid activity in pyruvate-containing media, as depletion of the NAD^+^ pool could limit the formation of the active isoniazid-NAD adduct (21).

Although the NADH/NAD^+^ ratio serves as an indicator of the cellular redox state, it does not provide information on the rate of NADH generation from NAD^+^, as this is masked by the rapid and continuous electron flux that dynamically interconverts the two cofactors. To assess this, we measured bacterial ATP content as an indirect readout of the dynamic electron transport flux from produced NADH. This analysis showed that bacteria grown in MMAT_GluGlyChl_ contained only 40% of the ATP found in bacteria grown in MMAT_GluFA_ (Fig. S4), supporting the hypothesis that bacteria grown in MMAT_GluFA_ have greater NADH production. In the presence of 0.4 µM BDM91465, bacterial ATP levels decreased in both media conditions, but the reduction was substantially greater in MMAT_GluFA_ (61% drop) than in MMAT_GluGlyChl_ (26% drop), further supporting the prominent role of Ndh-2 in the electron transport chain during growth in MMAT_GluFA_. Collectively, these data show that although the antibacterial activity of TriSLa differs between MMAT_GluFA_ and MMAT_GluGlyChl_, exposure to TriSLa induces similar changes in bacterial ATP levels and the NADH/NAD^+^ ratio (though to different extents), providing evidence of effective Ndh-2 inhibition in both media.

Previous studies have reported that an increase in NADH accumulation induced by antibiotic treatment can result in the generation of reactive oxygen species (ROS) (22, 23). As TriSLa causes an increase in the NADH/NAD^+^ ratio, we assessed if this redox change also caused ROS production in *Mtb*. To measure such ROS production, the 2′,7′-dichlorofluorescein diacetate assay was used (as reported in references 24, 25), and results revealed that TriSLa BDM91465 did not trigger a significant increase in ROS production, while this was observed for clofazimine, an antibiotic shown to induce ROS (26) (Fig. S3a and b). Furthermore, we found that the ROS scavenger thiourea (27, 28) did not alter the bactericidal activity of BDM91465 in either MMAT_GluFA_ or MMAT_GluFA_ without catalase, while this was observed for the moxifloxacin control (Fig. S3c and d) (28). Collectively, these data show that *Mtb* exposure to TriSLa did not lead to a detectable increase in ROS production in *Mtb*, though one cannot exclude that possible ROS production was neutralized by the bacterial antioxidant systems. An additional reason to support that TriSLa antibiotic activity is not primarily through ROS is that its antibiotic activity is slow and time-dependent, while it would be expected that ROS would lead to rapid killing.

### Limited *Mtb* transcriptional stress response to Ndh-2 inhibition by TriSLa

To characterize the early transcriptional response of *Mtb* to Ndh-2 inhibition, RNA sequencing analysis was performed following a 4 h exposure to TriSLa in both MMAT_GluFA_ and MMAT_GluGlyChl_. Under these conditions, 0.16 µM of BDM91465 was confirmed to decrease ATP levels of *Mtb* grown in both media, which served as a surrogate marker of Ndh-2 inhibition (Fig. S6). Under these early conditions of Ndh-2 inhibition by BDM91465, only a limited immediate transcriptional stress response was observed in either medium (Data set S1), although a more pronounced response may emerge at later time points. A similar, limited early transcriptional response has also been previously reported for other electron transport chain inhibitors, such as BDQ and AX-35 (29, 30). Despite the modest global response, several differentially expressed genes were identified. These changes included the overexpression of genes related to sulfate transport (*subi*, *cysW*, *cysT*) in MMAT_GluGlyChl_, the overexpression of *whiB3* in MMAT_GluFA_, and the down-regulation of *whiB7* in both media (Fig. S7). To investigate whether these transcriptional changes contributed to TriSLa susceptibility, genetically engineered Δ*whiB3* and Δ*whiB7* mutants of H37Rv were evaluated, but neither was found to have altered susceptibility to TriSLa compared with the wild-type strain (Table S3).

### Metabolomic and stable-isotope tracing analysis of *Mtb* following TriSLa exposure

To investigate the metabolic consequences of Ndh-2 inhibition by TriSLa, metabolomic profiling was performed on *Mtb* grown in MMAT_GluFA_ and MMAT_GluGlyChl_ following 24 h exposure to DMSO, BDM91465 (400 nM; 10× MIC), or bedaquiline (BDQ; 2 µg/mL; 10× MIC) as a control drug. As glucose was a common carbon source present in both MMAT_GluFA_ and MMAT_GluGlyChl_, stable-isotope tracing analysis was also performed by substituting glucose with ^13^C_6_-glucose. Metabolomics and metabolomics flux changes measured following exposure to BDQ (reference control) confirmed bacterial accumulation of ^13^C-(iso)citrate, α-ketoglutarate, and methylisocitrate, which are in line with previous reports (31) and helped validate assay setup (Fig. S8 and S10) (for full list, Data set S2).

**Fig 2:**
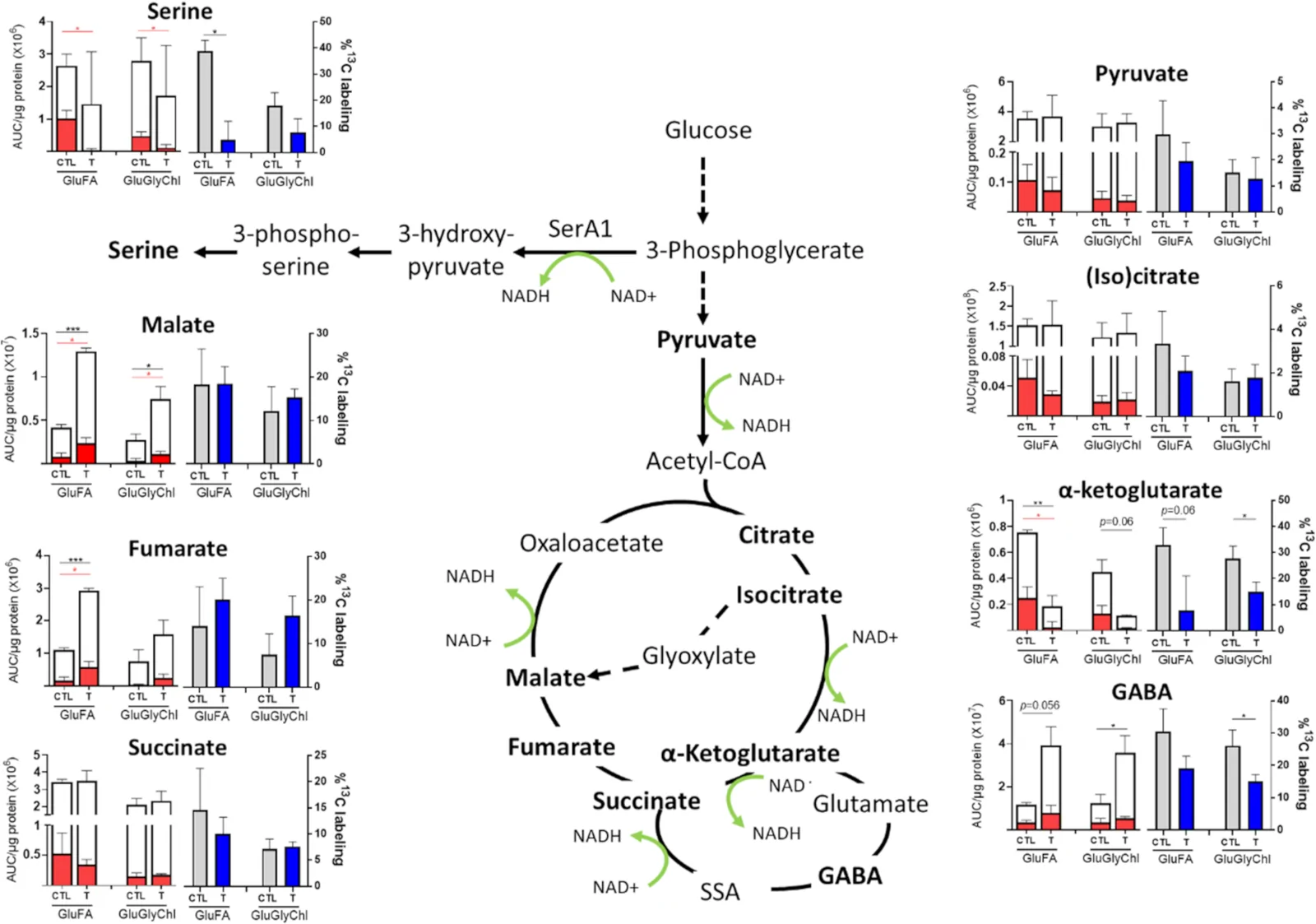
TriSLa partially rerouted the TCA cycle and had an impact on metabolic flux involved in serine biosynthesis. H37Rv was incubated with DMSO or TriSLa BDM91465 (0.4 µM) for 24 h in MMAT_GluFA_ or MMAT_GluGlyChl_ (using ^13^C_6_-glucose), after which metabolomics experiments were performed, and the metabolites of the TCA cycle extracted: pyruvate, (iso)citrate, succinate, malate, fumarate, and GABA (from GABA shunt), as well as serine. For each metabolite, bar charts on the left show (left y-axis) the total amount of ^13^C_6_-labeled metabolites (red bar) and unlabeled metabolites (white bar) measured. The right-hand bar charts (white and blue) show the percentage of ^13^C_6_-labeled metabolites (gray columns are for DMSO-treated samples, blue columns are for TriSLa-treated samples). Data are the mean and standard deviation of three biological replicates. (CTL): DMSO-treated samples; (T): TriSLa-treated samples. Statistical analysis was performed using t-tests with Welch’s correction. *P < 0.05; **P < 0.01; ***P < 0.001. Similar analysis for bedaquiline is shown in Fig. S8.

Focusing on the impact of Ndh-2 inhibition on metabolites of the TCA cycle, TriSLa exposure was found to have no significant impact on the absolute amounts and isotope labeling of bacterial pyruvate, (iso)citrate, or succinate. The presence of TriSLa did, however, significantly lower the level of measured bacterial α-ketoglutarate, with an accompanying reduction in flux from ^13^C_6_-glucose, in both MMAT_GluFA_ and MMAT_GluGlyChl_ (greater change in MMAT_GluFA_) (Fig. 2). Simultaneously, TriSLa induced the accumulation of GABA, fumarate, and malate in both media (again with a greater change in MMAT_GluFA_), though there were no significant changes in metabolic flux from ^13^C_6_-glucose. These changes in TCA cycle metabolites are consistent with reduced activity of NAD^+^-dependent TCA cycle enzymes following Ndh-2 inhibition by TriSLa, potentially driven by decreased NAD^+^ availability and/or an altered NADH/NAD^+^ redox balance, thereby promoting metabolite accumulation and metabolic rerouting. As an example, a lower activity of the NAD^+^-dependent malate dehydrogenase could explain the increased concentrations of malate and fumarate, while impairment of NAD^+^-dependent α-ketoglutarate dehydrogenase and/or NAD^+^-dependent succinate-semialdehyde dehydrogenase may lead to GABA accumulation. The observed reduction in α-ketoglutarate itself may reflect decreased activity of the NAD^+^-dependent isocitrate dehydrogenase, limiting α-ketoglutarate production, and shifting TCA flux through the glyoxylate shunt to form malate (which is increased), explaining the absence of isocitrate accumulation. Notably, the activation of the glyoxylate shunt has been described under growth conditions with high NADH as a mechanism of decreasing oxidative stress resulting from the increased NADH production, which is a consequence of Ndh-2 inhibition, though this hypothesis would require further validation (32). Finally, comparable metabolic alterations were observed in both MMAT_GluFA_ and MMAT_GluGlyChl_, indicating TriSLa-mediated Ndh-2 inhibition induced metabolic rewiring under both growth conditions. These shared metabolic responses suggest that the metabolic consequences of Ndh-2 inhibition are largely independent of the differential antibacterial activity of TriSLa observed between the two media.

In addition to metabolites in the TCA cycle, the study also elucidated the effects on amino acid production of TriSLa. The data showed that in both MMAT_GluFA_ and MMAT_GluGlyChl_, exposure to BDM91465 led to a significant shutdown in flux from ^13^C_6_-glucose to serine (Fig. 2). In agreement with arguments given for changes in the TCA cycle, this decreased flux from glucose to serine may be attributed to decreased activity of the NAD^+^-dependent 3-phosphoglycerate dehydrogenase (serA1) (33). Interestingly, previous studies show that supplementation of high concentrations of serine to *Mtb* can cause resistance to TriSLa and other Ndh-2 inhibitors, possibly to compensate for this decrease (10, 12). In addition to decreasing levels of serine, TriSLa was also found to interfere with the flux from glucose to glycine (probably through serine) in both MMAT_GluFA_ and MMAT_GluGlyChl_, and to histidine in MMAT_GluFA_ (Fig. S9). Inversely, we detected increasing amounts of alanine, lysine, tyrosine, and phenylalanine (largely independent of flux from glucose) following TriSLa treatment, with no current clear explanations. No significant changes were observed for glutamate or aspartate (Fig. S9).

### CRISPRi chemical-genetics profiling reveals *Mtb* vulnerability and tolerance to TriSLa

**Fig 3:**
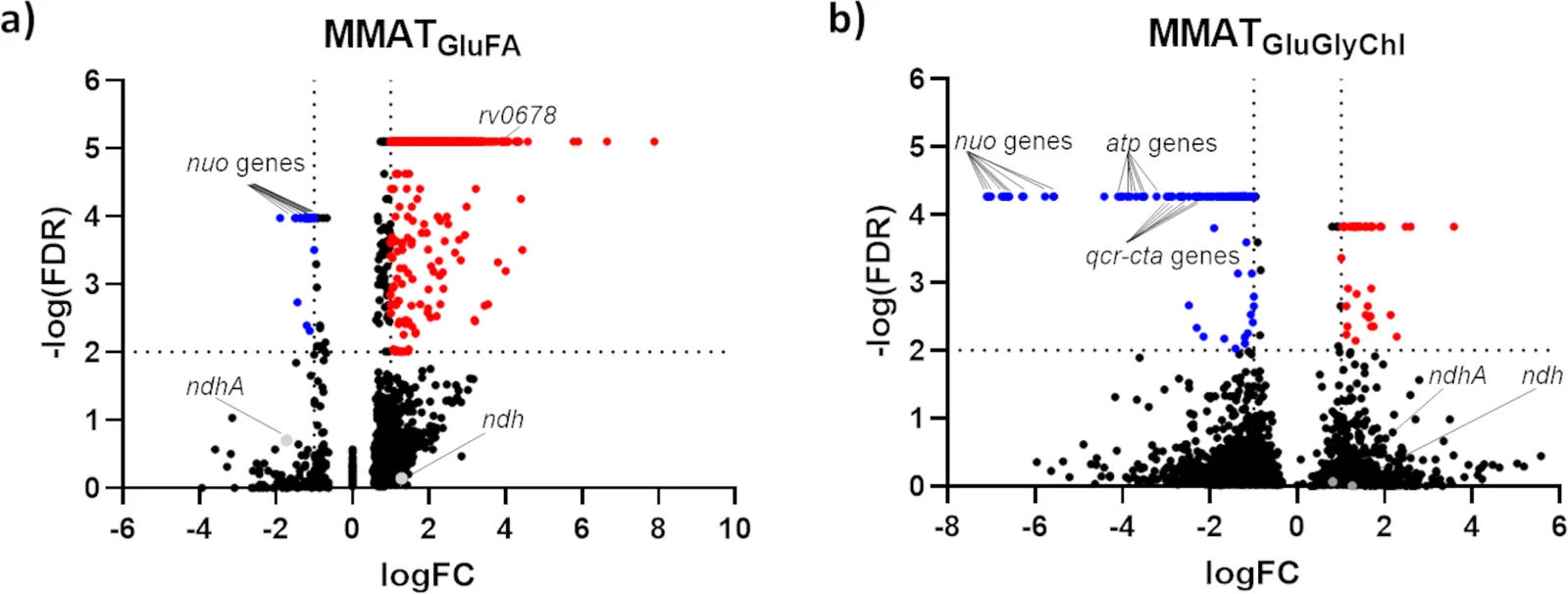
CRISPRi chemical-genetic screening of *Mtb* genes influencing the efficacy of TriSLa. Volcano plots depict the *Mtb* genetic vulnerability against sub-MIC concentrations of TriSLa BDM91465 in (**a**) MMAT_GluFA_ and (**b**) MMAT_GluGlyChl_. Significantly depleted hit genes (Log_2_FC ≤ −1 and FDR ≤ 0.01) are colored in blue, while significantly enriched hit genes (Log_2_FC ≥ 1 and FDR ≤ 0.01) are in red.

A CRISPRi chemical genomics screen was implemented in both MMAT_GluFA_ and MMAT_GluGlyChl_ (quality control of the library preparation was included in Table S9) to identify *Mtb* genes conferring vulnerability or resistance to Ndh-2 inhibition by TriSLa (34). In MMAT_GluFA_, the sub-MIC concentration of BDM91465 at 10 nM was selected as it achieved approximately 50% growth inhibition of the screening library (Fig. S11). Under this condition, the CRISPRi screen detected 25 significantly depleted genes in the presence of TriSLa (hyper-susceptible genes, FDR ≤ 0.01, Log_2_FC ≤ −1) and 645 loci significantly enriched in the presence of TriSLa (591 genes and 54 rRNA/tRNAs, hyper-tolerant genes, FDR ≤ 0.01, Log_2_FC ≥ 1). The overarching genes whose depletion resulted in hyper-susceptibility to BDM91465 in MMAT_GluFA_ were genes encoding the type I NADH dehydrogenase complex (13/25 genes, *nuoA-N*) (Fig. 3a; Fig. S12a), supporting the described synthetic lethality between Ndh-1 and Ndh-2 dehydrogenases in *Mtb* (full list of depleted genes in Data set S3) (18). On the opposite side, numerous genes were found to be strongly enriched, whose knockdown conferred *Mtb* tolerance against TriSLa. These included genes involved in central and bioenergetic metabolism, such as glycolysis (*ppgK*, *pfkA*, *fbA*, *gap*, *pgk*, *gpm1*, *eno*, *pykA*, *aceE*, *dlaT*, *gltA2*) and the TCA cycle (*acn*, *icd2*, *fum*, *mdh*) (Fig. S13), which may be explained by a consequential reduction in bacterial NADH production. Similarly, genes involved in lipid metabolism, including fatty acid uptake (*lucA* and mce1-mediated fatty acid uptake, including *yrbE1A-B*, *mce1A-F*, *lprK,* and *mkl*) and fatty acid β-oxidation (acyl-CoA ligases including *fadD2*, *fadD32,* and acyl-CoA dehydrogenases including *etfA*, *etfB*, *etfD*, *fadE5*, *fadE24*), were highly overrepresented (35, 36), which would be in agreement with their role in NADH production through fatty acid metabolism. The fact that mutations in fatty acid uptake and metabolism genes have not previously been isolated as TriSLa-resistant mutants may be because they have only a limited impact on activity, or because they have a high fitness cost on the media used for selection. Apart from central metabolism, there was enrichment of *mmpR5* (*rv0678*), the repressor of the mmpS5/L5 efflux transporter, hinting that TriSLa may be a substrate of MmpL5/S5 (37). Follow-up experiments confirmed that Rv0678 loss-of-function mutations conferred low-level (twofold) resistance to BDM91465 (Table S4). Finally, and in line with subsequent studies in this manuscript, this CRISPRi screen showed that numerous targets implicated in cell wall biosynthesis (*inhA*, *dprE1*, and *embB*) were enriched and thus antagonized by Ndh-2 inhibition in MMAT_GluFA_ (Table S10).

In MMAT_GluGlyChl_, where TriSLa is inactive, a higher concentration of BDM91465 (400 nM) was used for library selection, which induced only a small overall growth inhibition (approximately 40%). Under this screening condition, 104 genes were found significantly depleted in the presence of TriSLa (hyper-susceptible genes, FDR ≤ 0.01, Log_2_FC ≤ −1), and only 43 genes were significantly enriched (hyper-tolerant genes, FDR ≤ 0.01, Log_2_FC ≥ 1). Similar to MMAT_GluFA_, data showed that in MMAT_GluGlyChl_ the depletion of genes encoding for type I NADH dehydrogenase complex (*nuoA-N*) conferred sensitivity to BDM91465. However, uniquely to these media, the depletion of other ETC components was also associated with vulnerability to TriSLa, including the components of the cytochrome bcc:aa3 supercomplex (*qcrA*, *qcrB*, *qcrC*, *ctaD*, *ctaE* and *ctaB*) and the components of ATP synthase (*atpA-H*, *Rv1303*, and *Rv1312*) (Fig. 3b; Fig. S12b). In addition to the ETC, depletion of a number of genes encoding for NAD^+^-dependent enzymes also sensitized *Mtb* to TriSLa, including *ceoB-ceoC* encoding for TrK potassium transporter and multiple pyridine nucleotide transhydrogenase genes within an operon (*pntAa*, *pntAb*, and *pntB*) (Fig. S14; Data set S3). Notably, depletion of genes encoding the NAD^+^-dependent enzyme SerA1 (*serA1*), along with *serC* and *serB2*, that make up part of the serine biosynthesis pathway from glucose, induced *Mtb* to be hypervulnerable to TriSLa, which was in agreement with the metabolomic data. As with MMAT_GluFA_, a few genes involved in central metabolism were also highly enriched in MMAT_GluGlyChl_ (for full list, see Data set S3). Taken together, this CRISPRi chemical-genomic data illustrates the differential media-dependent vulnerability of *Mtb* to TriSLa, proposing a number of targets whose inhibition may synergize with BDM91465. In MMAT_GluGlyChl_, no enrichment of genes involved in cell wall biosynthesis was observed, as was seen for MMAT_GluFA_ (Table S10).

### Synthetic lethality between *Mtb* Ndh-1 and Ndh-2 chemical inhibition

Reports have shown that it is not possible to simultaneously delete Ndh-1, as well as both Ndh-2 genes (*ndh* and *ndhA*), and that genetic inactivation of *ndh-2* rendered *Mtb* to be hypersensitive to the Ndh-1 inhibitors (17, 18). This hypersensitivity to Ndh-1 inhibition by rotenone was further confirmed using a *de novo* generated H37Rv ndh-2 deletion strain (H37RvΔ*ndh*Δ*ndhA*) (Table S5). Furthermore, we observed that Ndh-2 loss sensitized *Mtb* toward the cytochrome bcc:aa3 inhibitor Q203 (Telacebec). In addition, against an Ndh-1 deletion strain (H37RvΔ*nuoF*), TriSLa BDM91465 became as active in MMAT_GluGlyChl_ as in MMAT_GluFA_ media, confirming that although TriSLa is still effectively able to inhibit Ndh-2 in MMAT_GluGlyChl_, Ndh-1 alone is sufficient to maintain the required NADH oxidation in this fatty-free condition.

**Fig 4:**
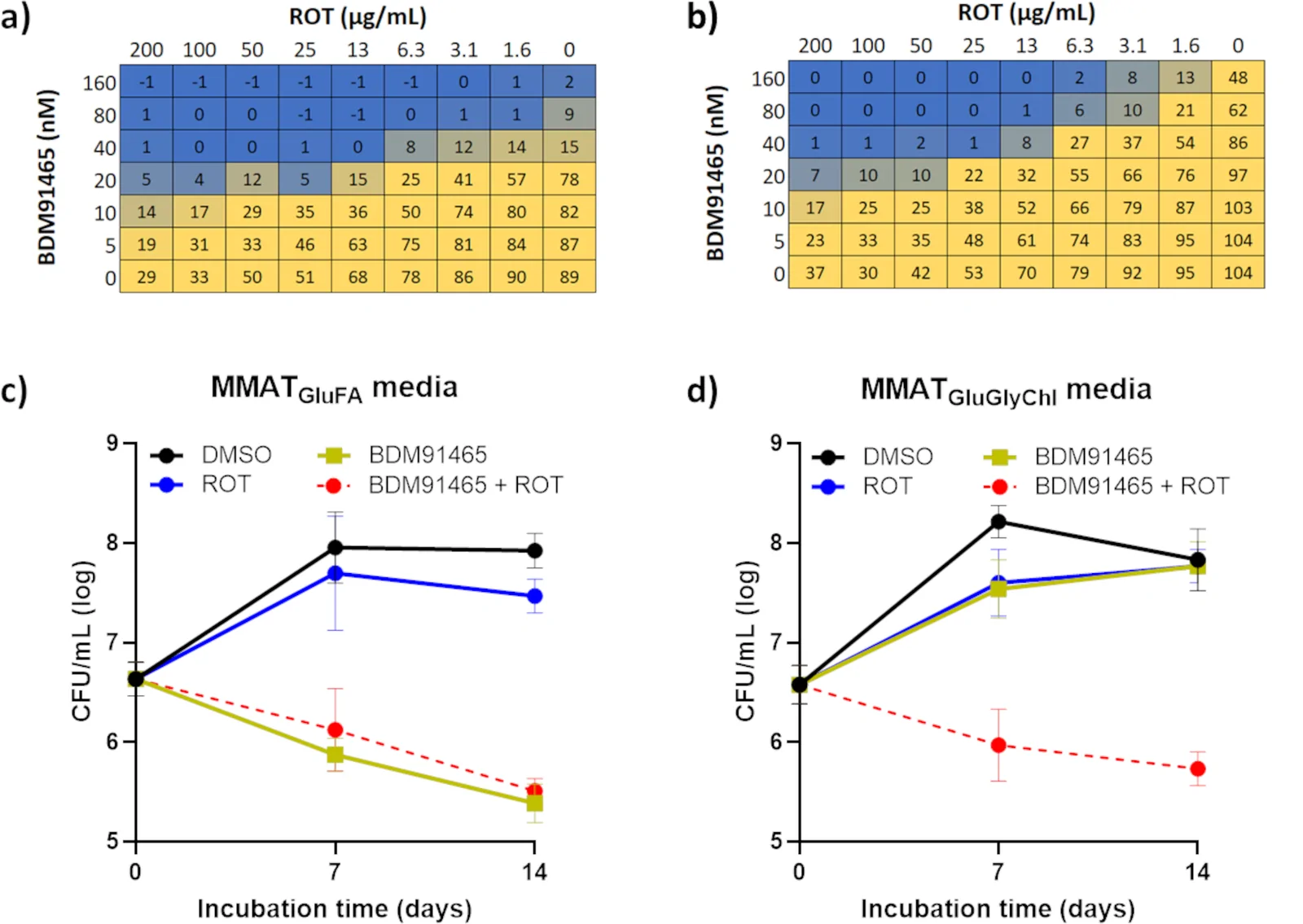
Synthetic lethality of Ndh-2 inhibition by TriSLa BDM91465 and Ndh-1 inhibition by rotenone. Checkerboard assay results for BDM91645 in combination with rotenone (ROT) against H37Rv-GFP *Mtb* growing in (**a**) MMAT_GluFA_ and (**b**) MMAT_GluGlyChl_. Bacterial growth of H37Rv-GFP was depicted as percentage of GFP fluorescence (excitation 485 nm, emission 510 nm) compared to the untreated control, and colored arbitrarily to facilitate analysis. The efficacy of the TriSla-rotenone combination was also measured in time-kill kinetics assays using an above MIC concentration of BDM91465 (0.4 µM) with rotenone (200 µg/mL) in both (**c**) MMAT_GluFA_ and (**d**) MMAT_GluGlyChl_. Data are presented as a mean and SD of at least three biological replicates.

To build on the synthetic lethality of *Mtb* NADH dehydrogenases, MIC-based checkerboard assays were performed with TriSLa BDM91465 and rotenone (Fig. 4a and b) (assays were also performed with an additional TriSLa BDM88689 [12]) and rotenone; Fig. S15), revealing clear synergistic anti-*Mtb* activity in both MMAT_GluFA_ and MMAT_GluGlyChl_ media. When the combination study was repeated on TriSLa-resistant H37Rv isolate RC1.1 (carrying a Y403H missense mutation in Ndh), no synergy was observed, validating that the TriSLa-rotenone synergy is mediated through inhibition of Ndh (Fig. S16) (12). Time-kill assays with BDM91465 (0.40 µM, 10× MIC), rotenone (200 µg/mL), or their combination revealed that in MMAT_GluFA_, the time-dependent bactericidal activity of TriSLa was not improved in combination with rotenone compared to the inhibitors alone, pointing to the dominant role of Ndh-2 in this medium (Fig. 4c). In MMAT_GluGlyChl_, neither rotenone nor BDM91465 were able to suppress *Mtb* growth, but in combination, they clearly synergized to become bactericidal to the same level observed in MMAT_GluFA_, showing that both Ndh-1 and Ndh-2 are able to manage the required NADH oxidation in the absence of fatty acids (Fig. 4d). Taken together, these observations support the synthetic lethality of Ndh*-*1 and Ndh*-*2 in *Mtb*, as demonstrated previously through genetic and chemical inhibition (18).

### Combination studies of TriSLa with anti-*Mtb* drugs

Combination studies between TriSLa and a panel of anti-*Mtb* clinical or preclinical drugs were performed in both MMAT_GluFA_ and MMAT_GluGlyChl_ media using two approaches. In the first approach, antibiotic combinations were evaluated on their ability to prevent bacterial growth (thus at near MIC antibiotic concentrations) using checkerboard assays. For the second approach, the “maximal” bactericidal activity of antibiotics was analyzed (by CFU counting) both alone and in combinations using antibiotic concentrations greater than their MICs (approximately 5–10 times). This approach aims to investigate whether TriSLa and other antibiotics can boost or interfere with each other’s maximal *Mtb* killing efficacy, mimicking the effective concentrations of the *in vivo* exposure situation.

**Fig 5:**
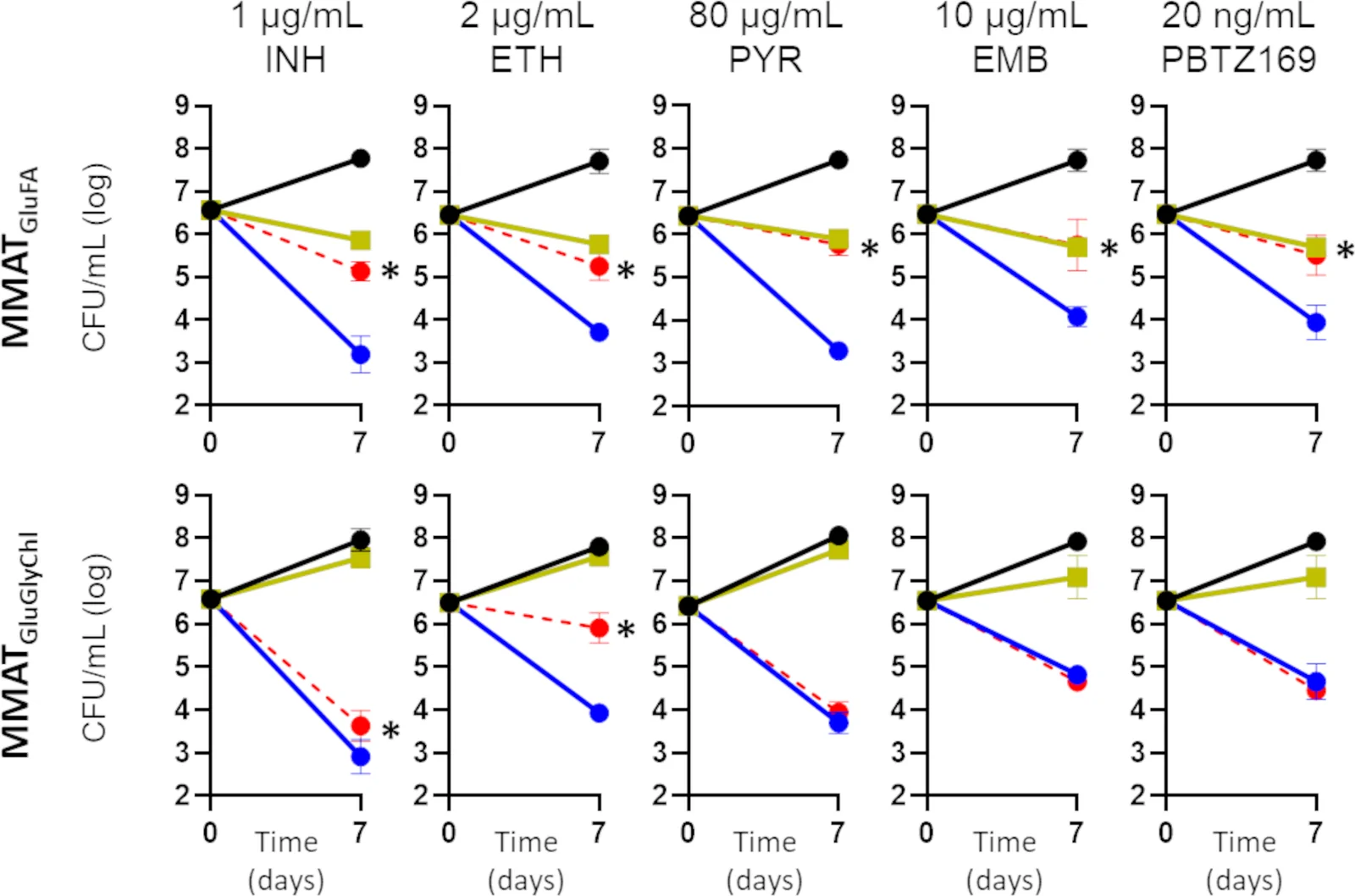
TriSLa antagonized the bactericidal activity of cell wall biosynthesis inhibitors in a medium-specific manner. Bactericidal activity of 0.4 µM TriSLa (BDM91465; 10× MIC), alone or in combination with above-MIC concentrations of isoniazid (INH, 1 µg/mL), ethionamide (ETH, 2 µg/mL), pyridomycin (PYR, 80 µg/mL), ethambutol (EMB, 10 µg/mL), and macozinone (PBTZ169, 20 ng/mL), was assessed by CFUs in MMAT_GluFA_ (top) and MMAT_GluGlyChl_ (bottom) media. Black lines indicate DMSO-treated controls; yellow/green lines, 0.4 µM BDM91465; blue lines, antibiotics alone; and red dashed lines, antibiotic-BDM91465 combinations. Data represent mean ± SD of at least three biological replicates. Asterisk highlights antagonistic combinations. For isoniazid, ethionamide, and pyridomycin antibiotic combinations, the experiments were continued up to 14 days and are shown in Fig. S19.

The first part of the combination studies aimed to determine the anti-*Mtb* activity of TriSLa BDM91465 (as well as BDM88689) in combination with antibiotics inhibiting mycobacterial cell wall biosynthesis; in particular, InhA-targeting prodrugs; isoniazid (INH) and ethionamide (ETH), direct InhA inhibitor pyridomycin, EmbA/B targeting ethambutol (EMB), and the DprE1 inhibitor macozinone (PBTZ169). The results from these checkerboard assays (Fig. S17 for BDM91465 and Fig. S18 for BDM88689) found no clear evidence of synergy or antagonism in either MMAT_GluFA_ or MMAT_GluGlyChl_. In contrast, analysis of the combinations in the CFU assay with higher than MIC concentrations of antibiotics revealed different results. In MMAT_GluFA_, all cell wall-targeting antibiotics alone caused an expected >2-log drop in bacterial load over 7 days, while TriSLa alone caused the expected 0.5 log drop in CFU. The bactericidal activity of the combinations, however, revealed clear antagonism, as the combination was shown to be much less effective than the cell wall-targeting antibiotic alone (Fig. 5). In MMAT_GluGlyChl_, where cell wall-targeting antibiotics maintained strong bactericidal activity, whereas TriSLa could not manage to prevent bacterial growth, this antagonism was no longer observed, particularly for the combinations with pyridomycin, EMB, or PBTZ169. The MMAT_GluFA_-specific antagonism seen between TriSLa and cell wall biosynthesis targeting inhibitors is in line with target vulnerability findings from the CRISPRi screen (Table S10). Despite the loss of antagonism seen between TriSLa and most cell wall inhibitors in MMAT_GluGlyChl_, antagonism was retained with the prodrugs INH and ETH in these media, but not with the direct InhA inhibitor pyridomycin, and thus likely not target-mediated. Instead, these findings suggest that TriSLa impairs the efficient bioactivation of INH and ETH into their active NAD^+^ adducts, likely by altering the bacterial NAD^+^/NADH redox balance. The impact of TriSLa in combination with INH, ETH, or pyridomycin was also evaluated for a prolonged period, and these experiments did demonstrate that the addition of TriSLa prevented time-dependent regrowth seen with INH and ETH, thus likely preventing the emergence of resistance, though this was not confirmed (Fig. S19).

A second series of combination studies focused on the combinations of TriSLa BDM91465 with rifampicin (RIF), linezolid (LNZ), *para*-amino-salicylic acid (PAS), or pretomanid (PRE), streptomycin (STR), and moxifloxacin (MOX) (Fig. S20 and S21). Similar to the cell wall inhibitors, STR and MOX revealed a similar interaction pattern, with checkerboard data showing an indifferent interaction while bactericidal assays indicated antagonism of the combination in MMAT_GluFA_ but not in MMAT_GluGlyChl_ within 1 week of treatment (Fig. S20). RIF activity was found not to be altered by the addition of TriSLa in either the checkerboard or CFU assay in both media. Combination studies between TriSLa and LNZ, PAS, or PRE overall displayed no obvious negative or positive interactions, though there were signs of mild antagonism in checkerboard assays in MMAT_GluFA_, which was not ultimately translated to bactericidal activity.

**Fig 6:**
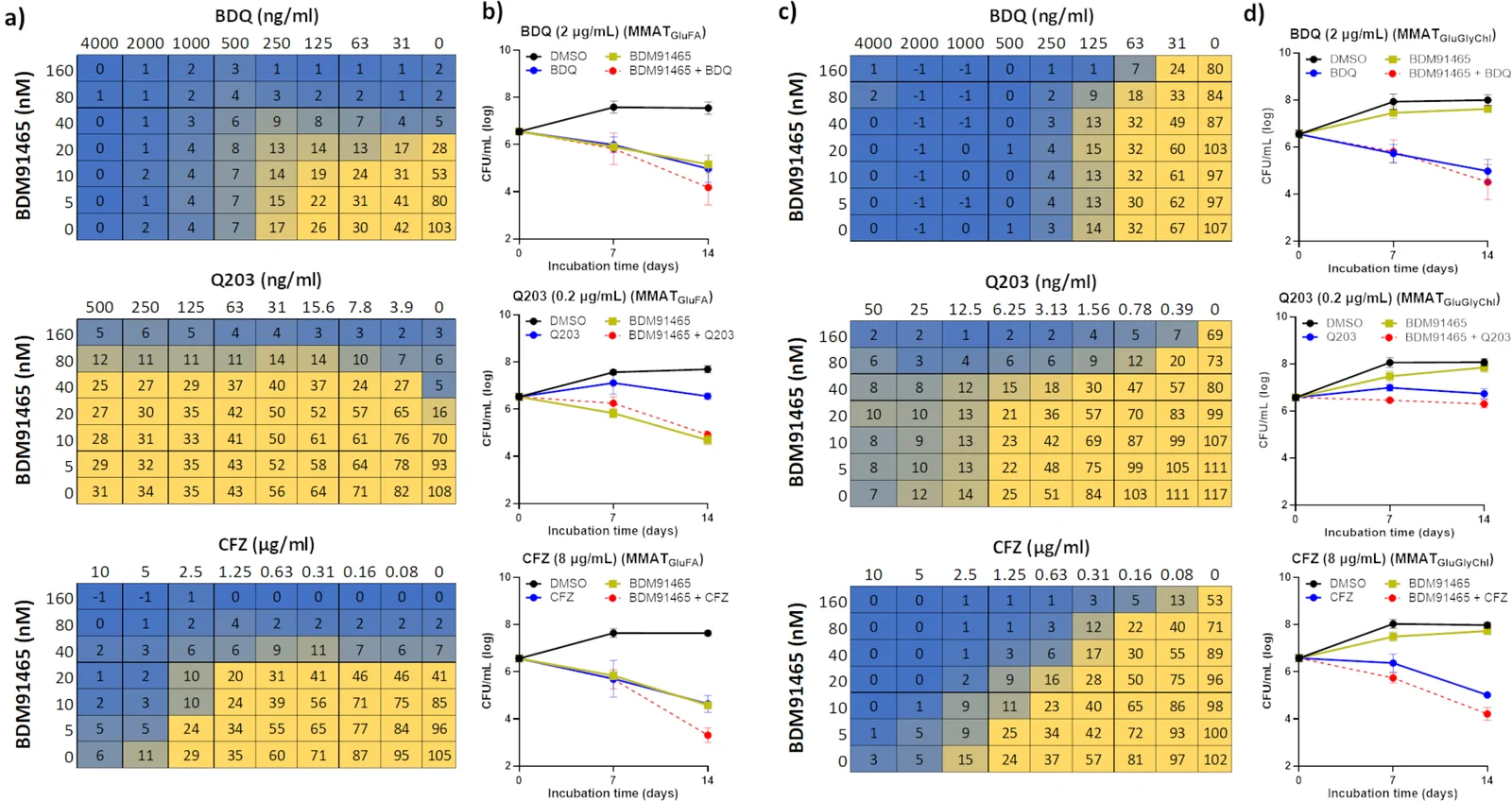
Potential synergistic activity observed between TriSLa and inhibitors of bacterial respiration. The anti-tuberculosis activity of TriSLa in combination with BDQ (top), Q203 (telacebec) (middle), and CFZ (bottom) was assayed by (**a, c**) checkerboard assays and (**b, d**) time-kill assays in both (**a, b**) MMAT_GluFA_ and (**c, d**) MMAT_GluGlyChl_. Checkerboard assay data depict the mean GFP fluorescence of H37Rv-GFP (following 1 week incubation) compared to the untreated control, and colored arbitrarily to facilitate analysis. Time-kill kinetic assays (**b, d**) were performed with 0.4 µM TriSLa BDM91465, alone or in combination with 2 µg/mL BDQ, 0.2 µg/mL telacebec (Q203), or 8 µg/mL CFZ and assessed by CFU. Data represent mean ± SD of at least three biological replicates.

Finally, TriSLa was evaluated in combination with other antibiotics targeting the ETC, specifically the ATP-synthase inhibitor BDQ, the cytochrome bcc:aa3 inhibitor telacebec, and clofazimine (CFZ), an antibiotic believed to be partially activated by oxidoreductases of the ETC (26, 38). In MMAT_GluFA_, checkerboard assays between TriSLa with BDQ, CFZ, or telacebec revealed a mild antagonism between these antibiotics at sub-MIC concentrations (Fig. 6; Fig. S22), while at above-MIC concentrations, time-kill assays indicated no clear detrimental interaction over 7 days, and even mild synergistic activity between TriSLa and BDQ and CFZ after 14 days (Fig. 6). In contrast, checkerboard assays in MMAT_GluGlyChl_ showed a mild synergy between TriSLa and BDQ (fourfold lower MIC_90_), and a more apparent synergy between TriSLa and telacebec (16-fold lower MIC_90_) and CFZ (eightfold lower MIC_90_) (Fig. 6). This observed synergy in MMAT_GluGlyChl_ translated to observed synergy in the time-kill assays where TriSLa combinations led to a greater bactericidal activity compared to the antibiotics alone (approximately 0.5-log greater drop for TriSLa/telacebec and 0.8-log greater drop for TriSLa/CFZ). Based on the observed synergy between TriSLa and telacebec, we also confirmed that this synergy is seen with other inhibitors of the cytochrome bcc:aa3, which are structurally unrelated to telacebec, including AX-35 (29) and QcrAB inhibitor 11626252 (39) (Fig. S24 and S25). Finally, we confirmed that observed synergy was dependent on Ndh-2 inhibition by repeating combination studies with TriSLa-resistant strain RC1.1 (Ndh mutation Y403H) (Fig. S23) (12), and it was verified that this synergy was also observed when bacteria were grown in a previously described fatty acid-free medium (Sauton’s minimal medium with glucose and glycerol as in Fig. S26) (15).

In an attempt to understand the mechanism behind the synergy seen between TriSLa and inhibitors of cytochrome bcc:aa3, we decided to measure the impact of these combinations on bacterial oxygen consumption rate (OCR) in different media using Seahorse technology. Firstly, data confirmed a lower basal rate of bacterial respiration in MMAT_GluGlyChl_ compared to MMAT_GluFA_ (Fig. S27a and c)**,** in line with the expected change in NADH-derived electron flux. In either medium, neither telacebec (as previously published [40]) nor TriSLa caused an instant decrease in OCR, while their combination significantly decreased OCR in MMAT_GluFA_ (*P* = 0.002) and in MMAT_GluGlyChl_ (*P* = 0.057, not significant) (Fig. S27b and d). Since antibiotic synergy between telacebec and TriSLa was observed exclusively in MMAT_GluGlyChl_, whereas OCR synergy was more pronounced in MMAT_GluFA_, the data do not support a direct correlation between OCR and antibiotic synergy.

### Study of TriSLa-antibiotic combinations on infected macrophages

As well as extracellular bacteria, the impact of TriSLa/antibiotic combinations was evaluated by checkerboard assays on intracellular *Mtb* (RAW 264.7 macrophages infected with H37Rv-GFP *Mtb*). Data confirmed that BDM91645 and BDM88689 alone were not able to fully control the intracellular bacterial growth, with the highest concentration tested (160 nM and 1.6 µM, respectively) restricting intracellular growth to around 15% compared to the DMSO control. Checkerboard assays with a selection of 11 antibiotics revealed that in most cases there was neither clear synergy nor clear antagonism, with the exception of rifampicin, which showed a tendency to synergize with both TriSLas (Fig. S28). A confounding factor in the analysis of this data is that it is not well understood to what extent the rich culture media of the RAW 264.7 cells influence the carbon-source utilization of intracellular *Mtb*. As a result, this question should be addressed in future studies to understand the *Mtb* carbon-source preference relevant to *in vivo* infection.

### *In vivo* efficacy of the candidate TriSLa-containing combinations

**Fig 7:**
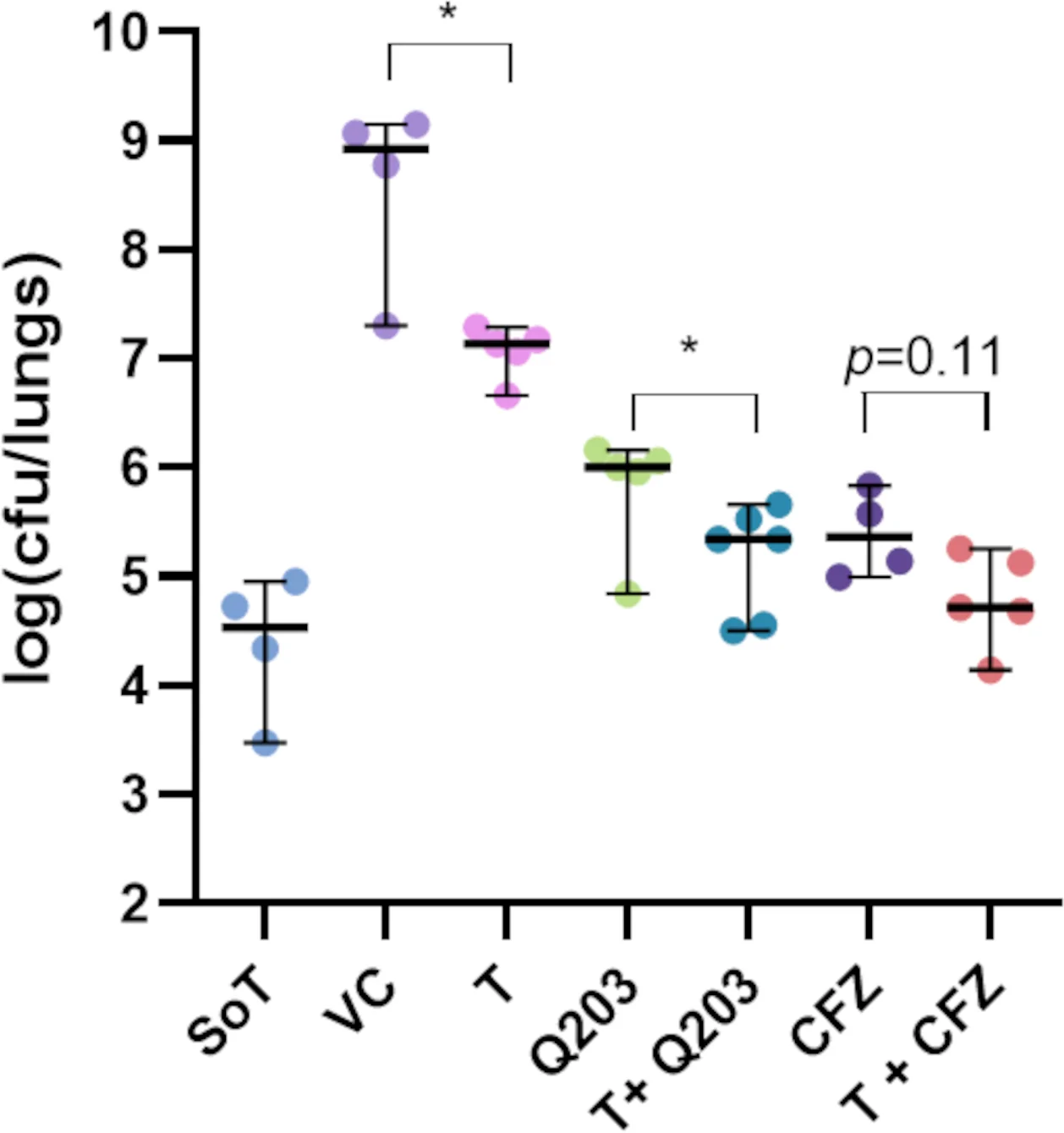
*In vivo* efficacy of TriSLa BDM91465 in combinations with telacebec and clofazimine in a murine model of acute tuberculosis infection. BALB/c mice were infected intranasally with H37Rv (10^5^ CFU), and treatment was initiated 3 days after infection and maintained for 2 weeks. Mice received either a vehicle control (VC); 60 mg/kg of BDM91465 (T); 10 mg/kg of telacebec (Q203), 20 mg/kg of CFZ; or a combination of 60 mg/kg BDM91465 with 10 mg/kg telacebec (T + Q203) or a combination of 60 mg/kg BDM91465 with 60 mg/kg clofazimine (T + CFZ). Bacterial loads (CFU) in the lungs were enumerated at the start of treatment (SoT, 3 days post-infection) and following 2 weeks of treatment. Statistical analysis was performed using a non-parametric Mann-Whitney U test (* *P* < 0.05).

Based on the CRISPRi data and the *in vitro* anti-*Mtb* activity of TriSLa combinations, we went further to verify whether the vulnerability and synergy observed in MMAT_GluGlyChl_ medium can be translated to the improvement of *in vivo* efficacy using a murine model of an acute *Mtb* lung infection. Specifically, we aimed to evaluate if a combination therapy of TriSLa (BDM91465, 60 mg/kg, p.o.) with standard effective doses of CFZ (20 mg/kg, p.o.) or telacebec (10 mg/kg, p.o.) would be better than the efficacy of the individual compounds (8, 41, 42). In this rapid acute model (treatment commenced 3 days post-infection, with daily treatment during 2 weeks), administration of BDM91465 alone resulted in the expected attenuation of lung bacterial growth, yielding approximately a 1-log reduction in CFU per lung compared to the vehicle control group (Fig. 7). Similarly, both telacebec and CFZ alone partially suppressed bacterial growth to a greater extent than TriSLa monotherapy, but still did not manage to fully limit the lung infection. The combination treatment with BDM91465/CFZ or BDM91465/Telacebec produced a non-significant trend toward enhanced bacterial clearance relative to the respective single agents (approximately 0.5-log additional reduction) (*P* ≈ 0.067 and *P* ≈ 0.068, respectively). Overall, these moderate combination effects are consistent with the *in vitro* findings, where BDM91465/CFZ and BDM91465/Telacebec combinations in MMAT_GluGlyChl_ medium outperformed single agents without evidence of antagonism. Collectively, these results support the potential incorporation of TriSLa in future combination regimens for tuberculosis treatment.

## DISCUSSION

Tricyclic spirolactams are an advanced class of type II NADH dehydrogenase inhibitors that exhibit potent but media-dependent “conditional’’ anti-*Mtb* activity. *In vivo* efficacy studies identified TriSLa to attenuate bacterial growth in an acute model of *Mtb*-infected mice, which was similar to the scenario of Ndh-2-deficient *Mtb in vivo* survival. These observations show that the overall efficacy of TriSLa observed *in vivo* resembled the TriSLa efficacy *in vitro* observed in media lacking fatty acids. With an objective to rationally design and understand how to achieve favorable synergistic interaction with Ndh-2 chemical inhibition, we initially set out to unravel the impacts of TriSLa on *Mtb* with the aim to discover *Mtb* bottlenecks induced by these compounds. Transcriptomic studies provided little information, as there appeared to be a limited immediate transcriptional stress response to TriSLa treatment, which was previously reported for other ETC inhibitors like BDQ or AX-35 (29, 30). Metabolomic data were, however, much more informative, revealing a number of important findings. Firstly, TriSLa exposure was identified to mediate the metabolic rerouting in the TCA cycle and amino acid biosynthesis independent of the culture media. As TriSLa is primarily active in FA-containing media, this finding highlighted the media-independent effects of Ndh-2 inhibition on central metabolism and that many of these metabolic changes do not lead to antibacterial activity. Secondly, we noticed that a lot of the metabolomic remodeling, including a potential rerouting through the glyoxylate shunt, was associated with NAD^+^-utilizing enzyme reactions, which would agree with TriSLa-mediated decrease in bacterial NAD^+^ concentration. Finally, in line with previously documented TriSLa resistance by media supplementation with serine, it was interesting to observe that TriSLa inhibited serine biosynthetic flux from glucose, whose pathway was partially NAD^+^-dependent. As serine biosynthesis is highly essential for intracellular *Mtb*, future studies should look into the therapeutic potential of the combination of TriSLa with *Mtb* serine biosynthesis inhibitors (19). Finally, to uncover *Mtb* genes that are particularly vulnerable upon TriSLa exposure, we performed the CRISPRi-chemical genetics screening. The method clearly highlighted multiple *Mtb* collateral vulnerability factors to TriSLa in media where Ndh-2 inhibition by TriSLa does not cause substantial growth inhibition, particularly components of Ndh-1, Cytochrome bcc:aa3, and ATP synthase. Taken together, these profiling data consolidated the role of TriSLa in altering the bacterial NADH/NAD^+^ ratio and proved to be a platform to discover collateral sensitivities of Ndh-2 inhibition, especially in media where TriSLa itself is unable to control growth.

For the analysis of the potential synergy and antagonism between TriSLa and other anti-*Mtb* compounds and drugs, evaluation of combinations was performed in different media, not only at the sub-MIC concentrations (checkerboard), but also at concentrations multiple times greater than the MIC. Evaluating the combinations at these high concentrations of antibiotics is essential to check how these antibiotics interact with TriSLa when they reach their maximal and therapeutic activities. Indeed, data confirmed that TriSLa combinations acted considerably differently at sub-MIC concentrations compared to combinations above MIC. A remarkable finding was that in FA-containing media, the slow time-dependent bactericidal activity of TriSLa (as measured by CFU) antagonized the efficient killing of antibiotics targeting cell wall biosynthesis (as well as fluoroquinolone), antagonism that was not observed at sub-MIC concentrations (checkerboard assays). These findings are in line with other published reports where a similar *in vitro* antagonism was shown between bacteriostatic and slow time-dependent bactericidal antibiotics targeting the ETC (BDQ and telacebec) and INH or MOX (43, 44). Our proposed explanation for this antagonism is that cell wall-targeting antibiotics and fluoroquinolones are often the most potent against actively replicating bacteria (or alternatively, non-replicating *Mtb* is tolerant to these antibiotics). Therefore, as TriSLa suppresses bacterial replication in these media, these cell wall inhibitors become less able to induce their bactericidal efficacies. This hypothesis is supported by TriSLa not antagonizing the same activity of pyridomycin, EMB, benzothiazinones, and MOX in media lacking fatty acids, where TriSLa does not prevent bacterial growth. The notable exceptions are combinations between TriSLa with INH and ETH, where antagonism is maintained in both media. The likely reason behind this result is not related to the antibiotic target (as there is no antagonism with direct InhA-targeting pyridomycin), but because these prodrugs require enzymatic activation to form active NAD^+^ adducts, and that this process becomes less efficient in the presence of TriSLa that alters the NAD^+^/NADH ratio (45). Finally, we noticed that while there was no synergy between TriSLa and cell wall-targeting antibiotic INH and PRE, time-kill assays showed that the combination of TriSLa with both drugs appeared to prevent the classical time-dependent regrowth of resistant strains to PRE and INH at the later time points. Taken that the *in vivo* efficacy of TriSLa mimics *in vitro* data with FA-free media, it would appear unlikely that the antagonism observed in FA-containing media between TriSLa and cell wall-targeting antibiotics would translate to antagonism *in vivo*, though this was not confirmed. However, for the case of INH and ETH, careful considerations should be taken into account as antagonism may indeed be expected.

Combination studies also revealed synergistic antibiotic activity between TriSLa and other inhibitors targeting or associated with the electron transport chain, in particular BDQ, telacebec, and CFZ. This synergy was particularly observed in FA-free media, both by checkerboard assays and by time-kill curves, and in agreement with the results from the CRISPRi analysis. These data confirmed once again that even when TriSLa exerts limited anti-*Mtb* effects in FA-free media, it still significantly remodels the physiological state of *Mtb*. In addition, with the hypothesis that FA-free media mimic TriSLa’s *in vivo* efficacy, it was a good sign to discover that TriSLa appeared to indeed moderately improve the infection control of both telacebec and CFZ in mice infected with *Mtb*. In summary, these data expand our understanding of the impact of Ndh-2 inhibition on *Mtb* and provide an important platform for the design of potential combination regimens based on TriSLa.

## MATERIALS AND METHODS

Detailed Materials and Methods are listed in the Supplemental material.

## Data Availability

Data set S1 shows the complete *Mtb* RNAseq transcriptomic data generated in the absence and presence of BDM91465. Data set S2 shows the complete data output of the metabolomics analysis of *Mtb* treated with BDM91465. Data set S3 shows results of the complete media-specific CRISPRi chemical-genetics analysis performed on *Mtb* treated with BDM91465. Raw reads of the CRISPRi chemical genetics, the transcriptomic analysis, and the whole-genome sequencing data of all the mutants were deposited to SRA under project PRJNA1424713.

## SUPPLEMENTAL MATERIAL

Data set S1 aac.00756-26-s0001.xlsx Transcriptomic stress response.

Data set S2 aac.00756-26-s0002.xlsx Metabolomics.

Data set S3 aac.00756-26-s0003.xlsx CRISPRi chemical genetics.

Supplemental material aac.00756-26-s0004.pdf Supplemental methods; Tables S1 to S10; Fig. S1 to S28.

ASM does not own the copyrights to Supplemental Material that may be linked to, or accessed through, an article. The authors have granted ASM a non-exclusive, world-wide license to publish the Supplemental Material files. Please contact the corresponding author directly for reuse.
