# Supplementary material for: Inhibition of type II NADH dehydrogenase in Mycobacterium tuberculosis reveals collateral vulnerability in the electron transport chain
Source: Antimicrob Agents Chemother. 2026 Sep 24;70(11):e00756-26. doi: 10.1128/aac.00756-26 (PMC13731233; doi:10.1128/aac.00756-26)
Supplement: Supplementary file 1 [file aac.00756-26-s0004.pdf]

# - Supporting Information -

Materials and Methods

Tables S1 to **S10**

Figures S1 to **S28**

SI References

Inhibition of type II NADH dehydrogenase in *Mycobacterium tuberculosis* reveals collateral vulnerability in the electron transport chain

Vien Q.T. Ho[1], Vincent Fontaine[2], Ernesto Anoz-Carbonell[1], Kamel Djaout[1], Clement Vigier[3], Leo Faion[3], Samsher Singh[4], Sushovan Dam[1], Jonathan Chatagnon[1], Stéphanie Slupek[1], Rudy Antoine[1], Aurélie Chauffour[2], Alexandra Aubry[2,5], Benoit Deprez[3], Kevin Pethe[4], Nicolas Veziris[2], Nicolas Willand[3], Robert Jansen[6], Baptiste Villemagne[3*], Ruben C. Hartkoorn[1*]

[1] Univ. Lille, CNRS, Inserm, CHU Lille, Institut Pasteur de Lille, U1019 - UMR 9017 - CIIL - Center for Infection and Immunity of Lille, Lille, France.

[2] Sorbonne Université, CNRS, Inserm, Centre d'Immunologie et des Maladies Infectieuses, CIMI, F-75013 Paris, France.

[3] Univ. Lille, Inserm, Institut Pasteur de Lille, U1177 - Drugs and Molecules for Living Systems, Lille, France.

[4] Lee Kong Chian School of Medicine, Nanyang Technological University, Singapore; Singapore Centre for Environmental Life Sciences Engineering (SCELSE), Nanyang Technological University, Singapore; National Center for Infectious Diseases (NCID), Singapore.

[5] AP-HP. Sorbonne Université, Centre National de Référence des Mycobactéries et de la Résistance des Mycobactéries aux Antituberculeux, Paris, France

[6] Radboud Institute for Biological and Environmental Sciences, Radboud University, Nijmegen, the Netherlands.

*__Corresponding authors__: Baptiste Villemagne & Ruben C. Hartkoorn

Email: baptiste.villemagne@univ-lille.fr, ruben.hartkoorn@inserm.fr

Materials and Methods

Bacterial strains, culture conditions, plasmids and generation of knock-out strains

Mycobacterium tuberculosis (*Mtb*) strains H37Rv or H37Rv-GFP (H37Rv carrying pJK-D6 (1)), as well as other *Mtb* strains indicated in **Table S6**, were routinely cultured in Middlebrook 7H9 supplemented (BD-DIFCO) with 10% commercial OADC (BD), 0.2 % glycerol and 0.05% Tyloxapol (Sigma Aldrich). Where indicated, *Mtb* cultures were switched to a previously described MMAT-based media (2). (1 g/L KH_2PO_4, 2.5 g/L Na_2PO_4, 0.5 g/L $(NH_4)_2SO_4$, 0.1 g/L L-Glutamine, 50 mg/L $MgSO_4$, 50 mg/L ferric ammonium citrate, 0.1 mg/L $ZnSO_4$, 0.5 mg/L $CaCl_2$, and 0.05% Tyloxapol), supplemented with 0.5 % fatty acid-free BSA (fraction V, Roche) and the carbon sources of interest. For $MMAT_{GluGlyChl}$, MMAT-base media was supplemented with a final concentration of 0.2% glucose, 0.2% glycerol and 0.02% cholesterol (prepared in ethanol stock). For $MMAT_{GluFA}$, MMAT-base media was supplemented with a final concentration of 0.2% glucose, 0.2 mM oleic acid and 0.0003% catalase (Sigma Aldrich). For metabolomic flux analysis, glucose was substituted with D-glucose-$^{13}C_6$ (Interchim SA). In several experiments, *Mtb* culture was switched to Sauton's media as previously described with minor modifications (0.5 % fatty acid free BSA, 0.5 g/L KH_2PO_4, 0.5 g/L $MgSO_4.7H_2O$, 2 g/L citric acid, 0.05 g/L ferric ammonium citrate, 0.001 g/L ZnSO4, 0.85 g/L NaCl, 0.1 g/L L-Asparagine together with 0.2 % glycerol, 0.2 % glucose, 0.0004% catalase) (3).

All the strains and plasmids used in the study were listed in **Table S6** and **Table S7**, respectively. *Mtb* knock-out strains of genes including *nuoF*, *sdh1*, *ndh*, *whiB3* and *whiB7* were generated using ORBIT recombineering system method as previously described (4). Briefly, H37Rv WT *Mtb* harboring plasmid pKM444 was co-electroporated with 1 µg of the attP-containing oligonucleotides targeting the indicated genes **(Table S8)** and 200 ng of the attB-containing plasmid pKM464. Transformants were selected on 7H11-Hygromycin agar plates and deletions were confirmed by PCR and Sanger sequencing of the 5′ and 3′ ends of the plasmid insertions with the primer couples also listed in **Table S8.** The replicative plasmid pKM444 and the integrated payload plasmid pKM464 were subsequently cured using the excisionase plasmid pKM512, encoding Bxb1 excisionase functions (gp47 and Int) and SacRB. Following ATc induction, the excisionase plasmid was counter-selected on 7H10 agar supplemented with 10% sucrose, yielding markerless knock-out strains carrying a single attB scar at the target locus. The genetic modification was confirmed by PCR and Sanger sequencing with the primers listed in

Table S8. Additionally, knock-out strains were found to be sensitive to hygromycin, kanamycin, and zeocin.

To generate the double knock-out strain H37Rv Δ*ndh* Δ*ndhA*, a frameshift mutation was introduced into *ndhA* in a H37Rv Δ*ndh* background by using a CRISPR-based system (5). Briefly, plasmid pCRISPR-ndhA was electroporated into H37Rv Δ*ndh*, and transformants were selected on 7H11 agar supplemented with kanamycin and 500 ng/mL anhydrotetracycline (ATc) to induce *cas9* expression. Individual colonies were isolated, and the Cas9-targeted genomic region was amplified by PCR and sequenced to identify frameshift mutants. This approach resulted in a 14 bp deletion (ΔGCTGATCCTGCGCC; c.219_232delGCTGATCCTGCGCC) in *ndhA* (*Rv0392c*), causing a frameshift mutation (p.Arg73fs). Finally, the plasmid L5 attB::empty was electroporated into the knock-out strain to replace pCRISPRx-Sth1Cas9-L5. For all knock-out strains, the absence of additional mutations was confirmed by whole-genome sequencing (SRA under Project PRJNA1424713).

Generated *Mtb* strains having deletions in *nuoF*, *sdh1* and *ndh-ndhA* were transformed with replicative plasmid pJK-D6 encoding the green fluorescent protein (GFP), a kind gift from Luciana Leite, Butantan Institute, Brazil (strains then renamed H37Rv Δ*nuoF*, H37Rv Δ*sdh1*, H37Rv Δ*ndh2* in Table S6). This plasmid carries de *gfp* gene under the control of a strong promoter generated through error-prone PCR of the PL5 promoter from the mycobacteriophage L5 resulting in high levels of GFP expression (1)

Chemistry and compounds used in the study

BDM88689 (6), BDM91465 (7) and AX-35 (8) were synthesized according to previously described methods (Purity > 95%). Pyridomycin was isolated from cultured *Dactylosporangium fulvum* according to previously published protocol (9). PBTZ169 and 11626252 were kind gifts from Dr. Vadim Makarov (Bakh Institute of Biochemistry, Russian Academy of Science, Moscow, Russia). All other antibiotics were purchased as following: bedaquiline (BDQ) (AiFChem and ChemShuttle), telacebec (AiFChem and MedChem Express), ethambutol (Cayman Chemical), Linezolid (Acros Organics and BLDPharm), moxifloxacin, pretomanid (MedchemExpress), rifampicin (Carbosynth), streptomycin (Euromedex) and clofazimine, ethionamide, isoniazid, para-aminosalicylic and rotenone (all from Sigma Aldrich). For experimental use and distribution, all compounds and antibiotics were dissolved in DMSO.

Minimal inhibitory concentration (MIC) and checkerboard assays

H37Rv *Mtb* strains (WT and mutants), and isogenic strains expressing GFP (transformed with pJKD6-GFP were used to determine the minimum antibiotic activity (MIC) of compounds using either the resazurin microtiter-based assay (REMA) or the bacterial GFP fluorescence signal (1). Briefly, bacteria were grown to mid-log phase (OD_{600} between 0.4 and 0.8) in Middlebrook 7H9 media supplemented with 10% OADC and 0.05% tyloxapol. Bacteria were then diluted to OD_{600}=0.001 in the culture media of choice, and added to 96-well flat bottom plates (Sarstedt) that either already contained the antibiotics of interest (added using Digital D300e dispenser from Tecan), or where antibiotics were added by serial dilutions. Plates were sealed with sticky PCR-SP-S sealing films (Axygen) and incubated for 7 days at 37°C. Bacterial load was then determined by either measuring GFP fluorescence (Excitation: 485 nm; Emission: 510 nm) or by addition of resazurin (0.01 %, overnight, 37°C) and measuring resazurin reduction by fluorescence (Excitation: 560 nm; Emission: 590 nm) using the EnSight™ Multimode Plate Reader (Perkin Elmer). MICs were defined as the lowest concentration of antibiotic that resulted in either < 5% of GFP signal, or < 5% of resazurin reduction. For checkerboard assays a similar protocol was followed as for MIC determination, except that initially the checkerboard compound combinations were dispensed into 384-well flat bottom plates (Greiner) using a D300e Digital Dispenser (Tecan), followed by the addition of 50 µL of bacterial suspension at the final OD_{600}=0.02.

Determination of time-dependent bactericidal antibiotic activity

To determine the time-dependent killing of bacteria by antibiotics, bacterial survival was measured by determining remaining CFU (Colony-Forming Unit). Briefly, a mid-log phase culture (OD_{600} between 0.4 and 0.8) of *Mtb* was diluted to an OD_{600}=0.02 in the desired culture and added to 96-well plates (Sarsted) containing the tested compounds. When indicated, *Mtb* was incubated with ROS scavengers Thiourea (10 mM) for 1 hour before treatment with the tested compounds. Plates were sealed and incubated at 37°C for 7 days or 14 days after which bacterial viability was determined by plating 50 µl of 1 in 10 bacterial dilutions onto Middlebrook 7H11 agar having 0.5% glycerol and 10% OADC at 37°C. CFU were then determined from week 3 to week 6 after plating, to determine the number of bacteria remaining after compound exposure.

Determination of antibiotic activity on intracellular *Mtb*.

The assessment of antibiotic activity on intracellular *Mtb* in RAW 264.7 was performed according to the previously described protocols (10) with minor modifications. In a T75 flask (Corning Inc), confluent RAW 264.7 (ATCC # TIB-71) cells growing in of RPMI media supplemented with 10%

FBS were infected with H37Rv-GFP (pJKD6-GFP) *Mtb* at MOI of 2 for 2h at 37 °C. After infection, supernatant was removed and cells were washed twice with PBS. Cells were then detached, collected by centrifugation at 1400 rpm and resuspended in RPMI media. Subsequently, 50 µL of the cellular suspension was added to the 384-well assay plates (20,000 cells/well) having wells filled with the indicated compounds by Tecan D300e dispenser. Plates were incubated for 5 days at 37°C, 5% CO_2. Confocal images were recorded on an automated high-content fluorescent microscope (InCell 6500, Cytiva). Images of four fields per well were acquired, covering almost the entire surface of the wells. Each image was then analyzed using Columbus software to quantify the GFP signal of the bacteria.

Detection of Reactive Oxygen Species (ROS)

The ROS detection assay was performed as previously described (11, 12). *Mtb* was grown in $MMAT_{GluFA}$ to the OD_{600} 0.4-0.6. Bacteria was then washed and re-suspended in Middlebrook 7H9-Tween (0.05%) containing 40 µM 2′,7′-dichlorofluorescein diacetate (DCFDA) and incubated in the dark at 37ºC for 30 minutes. Bacteria was then washed and resuspended in Middlebrook 7H9-Tween. Bacteria loaded with DCFDA were then dispensed into black-walled clear-bottom 96-well plates (Sarstedt) that already contained the desired test compounds. After 24 h and 48 h of incubation, fluorescence intensity (Ex485/Em535) was measured using an EnSight™ Multimode Plate Reader (Perkin Elmer).

Quantification of intracellular ATP following exposure to antibioitics

BDM91465 and BDQ were dispensed into the wells of white polystyrene 96-well plates (Corning Inc) at the indicated concentration by D300e Digital Dispenser (Tecan). H37Rv *Mtb* in mid-log phase resuspended in $MMAT_{GluFA}$ or $MMAT_{GluGlyChl}$ was then added to each well (50 µl) to the final OD_{600} of 0.01. After 4h or 24h, bacterial ATP levels were measured using the BacTiter-Glo reagent (Promega) as per manufacturer's instructions, and luminescence measured using an EnSight™ Multimode Plate Reader (Perkin Elmer) and the final values were normalized to the biomass represented by the GFP signal (for treatment after 24 hours).

Determination of bacterial NADH/NAD$^+$ ratio

The *Mtb* NADH/NAD$^+$ ratio was determined as previously described (6). Briefly, H37Rv *Mtb* were grown to mid-log phase in $MMAT_{GluFA}$, $MMAT_{GluGlyChl}$ or $MMAT_{GluPyr}$ and then incubated with DMSO, BDM91465 (0.4 µM) or BDQ (2 µg/mL) for 4 h or 24 h. *Mtb* cells were then pelleted from 12 mL of each group and then resuspended in 300 µL of 0.2 M HCl (for NAD$^+$ extraction) or 300 µL of

0.2 M NaOH (for NADH extraction). Cells were transferred to 2 mL Lysing Matrix B tubes (MP Bio) tubes and lysed using a MM 400 mixer mill (Retsch GmbH, Haan, Germany). Samples were subsequently incubated at 55ºC for 10 minutes and then cooling on ice. NAD^+ or NADH from samples were then neutralized with 300 µL 0.1 M NaOH or 300 µL 0.1 M HCl, respectively. Finally, NAD^+ and NADH amount of each sample was determined using the NAD^+/NADH-Glo™ assay (Promega) according to the manufacturer's instructions. For each experiment, a NADH standard curve with a blank control was added in addition to the main samples.

Determination of Oxygen consumption using the Seahorse XFe96 analyzer

H37Rv *Mtb* were cultured to mid-log phase (OD_{600} between 0.4 and 0.8). Subsequently, *Mtb* culture was washed and diluted to OD_{600} of 0.4 in either $MMAT_{GluFA}$ or $MMAT_{GluGlyChl}$ and cultured for 24h before the assay. Oxygen consumption rates (OCR) were measured using the Seahorse XFe96 Analyzer (Agilent). Agilent Seahorse cell culture microtiter plates were coated with 22.4 µg/mL Corning® Cell Tak (Corning Inc). *Mtb* cells were then washed and resuspended in the indicated MMAT media at the OD_{600} of 0.4. 50 µL of bacterial suspension was added to each well and centrifuged to adhere the bacteria to the cellTak coated culture microplate (Agilent), followed by additional 130 µL of respective media. Tested compounds or drugs was injected in real-time after 5 basal readouts. Final concentration of DMSO was 1%, used as drug vehicle. Oxygen consumption rates (OCR) were then monitored using the Seahorse XFe96 Analyzer with the OCR was derived from 4 min of continuous measurements. Each data point was interspaced with 2 min of continuous mixing. Compounds tested were injected in real-time through the injection port. All data were analyzed using the Wave Desktop 10.1.2, as well as the GraphPad Prism 10 software.

RNA isolation and transcriptomic profiling

H37Rv *Mtb* was cultured in $MMAT_{GluFA}$ or $MMAT_{GluGlyChl}$ to mid-log phase. Subsequently, 10 mL of *Mtb* at OD_{600} = 0.4 was incubated with BDM91465 (0.16 µM) or DMSO in 50-mL tubes (Corning) for 4h at 37ºC. The *Mtb* pellet was collected by centrifugation at 3200 g for 5 min. The pellet was resuspended in 1 mL of RNApro™ (FastRNA® ProBlue Kit, MP biomedicals) and homogenized in impact-resistant 2 mL tubes containing 0.1 mm silica spheres (Lysing Matrix B, MP biomedicals) using a FastPrep FP120 cell disrupter (Thermo Fisher Scientific) at 6.5 m/sec for 40 sec. The lysed cells were centrifuged at 10,000 x *g* for 10 min to remove cellular debris and total RNA was extracted following the instructions of FastRNA® ProBlue Kit (MP Biomedicals). For further purification (to remove possible contaminating DNA), 5 µg of total RNA extract was treated twice

with DNase (Sigma Aldrich) followed by purification by AMPure XP beads (Ampure Beckman). Ribosomal RNA (rRNA) depletion was performed on 1 µg of RNA using QIAseq FastSelect – 5S/16S/23S Kits (Qiagen, reference 335925). Libraries for Illumina sequencing were prepared with QIAseq Stranded RNA Library kit (Qiagen, reference 180743). Illumina NextSeq 500 system (Illumina, Inc.) was used in a high-output mode according to the manufacturer's protocols for the sequencing of cDNA libraries. All samples were multiplexed on one lane of the flow cell and sequenced in single-read sequencing mode with read lengths of 150 base pairs (bp). Raw RNA-seq reads were processed with Illumina quality control tools using default settings. Mapping all the reads on *Mtb* rRNA sequences using Bowtie2 (http://bowtie-bio.sourceforge.net/bowtie2/index.shtml) allowed filtering out of residual rRNA-specific reads. RNA sequencing data analysis was performed using the SPARTA pipeline with default parameters (13). Briefly, the raw reads are first processed with Trimmomatic to remove Illumina adapter sequences and low-quality reads. The quality of the reads is then assessed using fastQC. The reads were mapped on the *Mtb* H37Rv reference genome using Bowtie. The number of reads mapped to the different features was calculated using HTSeq-count. The differential gene expression analysis was done using the edgeR package. Potential Batch Effects were detected using MultiDimensional Scaling (MDS) plot.

CRISPRi chemical-genetic screening

Library transformation in *E. coli*: The plasmid library (Addgene 163954) was electroporated into MegaXDH10BTM T1R ElectrocompTM cells (Invitrogen, C64900-03) by following the Addgene provided protocol. Briefly, five transformations were set up and plated on LB-Kanamycin (50 µg/mL). A total of 2.79×10^7 transformants were obtained, representing a 289-fold coverage of the library. Transformants were scrapped off the LB-agar plates and inoculated into 1 L LB with 50 µg/mL kanamycin and grown overnight. The cells were harvested by centrifugation and the plasmids were extracted using CompactPrep Plasmid Maxi kit (Qiagen, 12863), spread over 6 columns, following manufacturer instructions. The plasmid library was eluted in nuclease free water, aliquoted and stored at -20 °C.

Library transformation in H37Rv *Mtb*: H37Rv *Mtb* was grown in 7H9 (BD, Difco) supplemented with glycerol (0.2%), Tween-80 (0.05 %) and OADC (BD, Difco) to the OD_{600} of 0.37. Bacterial cells were washed three times in a solution of glycerol (10 %) and Tween-80 (0.05%) and concentrated 50-fold. Sixty transformations were set up where 200 ng of plasmid pool was added to 100 µl of cells. Electroporation was performed with Gene Pulser (Bio-Rad) at 1000 ohms, 2500

V and 25 µF in a 2 mm cuvette (Fisher Scientific, 15542423). Electroporated cells were diluted 25-fold in fresh 7H9 and incubated at 37°C for 24 hours. A sample of the recovery bacterial suspension was diluted and pated on 7H11 with 25 µg/mL kanamycin to count transformants (3.97 10^7 transformants obtained, representing 410-fold coverage of the library). The rest of the bacterial recovery suspension was spread on 7H11 with 25 µg/mL kanamycin (12x12 cm plates, 1 plate per transformation) and incubated at 37°C. After 21 days of outgrowth, bacterial cells were scrapped off the agar plates, pooled, homogenized and stocked at -80°C with 15% glycerol (60 primary stock tubes total). Three primary stock tubes were unfrozen and grown for 4 days in 200 ml 7H9 supplemented with glycerol (0.2%), Tween-80 (0.05 %) and OADC (BD, Difco) to declump the bacterial population. The culture was then centrifuged for 2 mins at 100 g (room temperature). The pellet was discarded and the remaining bacterial population was adjusted to the OD_{600} of 1 with 15% glycerol, aliquoted (1 mL per tube) and stocked at -80°C. These tubes served as bacterial input in the CRISPRi screen.

CRISPRi screening: CRISPRi chemical-genetic screening using the *Mtb* CRISPRi library RLC12 (Addgene #163954) was performed as previously described (14, 15). All the steps were performed in the T-75 vented tissue culture flasks (Sarstedt) at 37°C. Three independent H37Rv *Mtb* populations transformed with RLC12 CRISPRi library were cultured to the mid-log phase in $MMAT_{GluFA}$ or $MMAT_{GluGlyChl}$ media, representing biological triplicates. Kanamycin (20 µg/mL) was maintained throughout the experiment. The bacterial suspensions were then diluted back to OD_{600}=0.1 and treated with 100 ng/mL anhydrotetracycline (ATc) (IBA Lifesciences) for 5 days. After sgRNA target pre-depletion, the chemical-genetic screen was initiated by inoculating triplicate cultures at an OD_{600} of 0.1, in the presence of 100 ng/mL ATc and treated with DMSO or the indicated BDM91465 concentration in the same MMAT media as utilized in the pre-depletion step for 14 days. After every 5 days, cultures were washed in PBS and diluted back to OD_{600} of 0.1 with replenished ATc, kanamycin and DMSO or BDM91465. After a total of 14 days of treatment, bacterial pellets were collected by centrifugation (4000 g, 5 mins) and stored at -20°C.

Genomic DNA for each pellet were extracted using CTAB-Lysozyme as previously described (16). Bacterial pellets were first suspended in 800 µl TE buffer and incubated with lysozyme (15 mg/mL) overnight at 37 °C. Subsequently, 70 µl SDS 10% and 5 µl proteinase K (20 mg/mL) were added and samples were incubated for 30 minutes at 65°C. Next, 200 µl NaCl (2.5 M) and 80 µl CTAB 10% were added and samples were incubated for another 30 minutes at 65°C. Then 750 µl ice-cold chloroform was added and samples were mixed thoroughly. Aqueous phase was collected

for each sample and removed from BSL-3 lab after centrifugation for 16.000g for 5 minutes. After 30 mins of treatment with 25 µg of RNase A (Invitrogen) at 37 °C, samples were extracted with 1 volume of phenol:chloroform:isoamyl alcohol (Invitrogen). The aqueous phase was further extracted with 1 volume of chloroform. Finally, genomic DNAs were then precipitated by addition of 0.6 volumes of isopropanol and 0.02 volumes of sodium acetate (3 M) and incubated overnight at -20 °C. Genomic DNA was washed one with 80% ethanol and resuspended in 50 µl nuclease-free water. DNA concentration was determined with a Qubit BR dsDNA assay kit (Thermo Fisher). CRISPRi sequencing libraries were prepared as previously described (14). Amplification was verified by 2% agarose gel electrophoresis. Amplicons ~230 bp were purified by double sided bead selection (Macherey-Nagel 744970) using a ratio of 0.6 and 1 and quantified using KAPA SYBR FAST qPCR kit (Roche KK4600). Libraries were sequences on Illumina Novaseq PE150 platform at 600-fold depth.

Sequences of sgRNAs were extracted from single end reads using *cutadapt* from their cloning site (between [GTGATAGATATAATCTGGGA.. ..GTTTTTGTAGCTCGAAAGAAG]). Sequences shorter than 4 bases were discarded (-*m* 4) (17). Extracted sequences were mapped and counted to the RLC12 sgRNA libraries using MAGeCK *count* function (18). MAGeCK *test* function was used for Robust Rank Aggregation.Directional calling for each gene was done as previously described elsewhere (15). MAGeCK outputs and final dataset used for volcano plots are supplied in supplementary files.

Metabolomics and Stable-isotope tracing of *Mtb* treated with BDQ and BDM91465

H37Rv *Mtb* was cultured in $MMAT_{GluFA}$ or $MMAT_{GluGlyChl}$ to mid-log phase (0.4-0.6) in 20 ml. Subsequently, *Mtb* pellet was collected, washed with PBS, resuspended in $MMAT_{GluFA}$ or $MMAT_{GluGlyChl}$ containing $^{13}C_6$-Glucose (Interchim SA) without Tyloxapol and incubated with DMSO, BDM91465 (0.4 µM) or bedaquiline (2 µg/mL) for 24h. Bacterial pellets were then collected by centrifuged at 4000 g in 5 minutes at 37°C. Supernatants were gently removed and pellets were then resuspended in 1 mL of cold quenching buffer (acetonitrile:methanol:water in a ratio 2:2:1, v/v/v with 0.1 M formic acid) and added into a cooled FastPrep tube (Lysing Matrix B, 2mL tubes, MP Biomedicals: 6911100). Samples were stored at -80°C. For metabolites collection, pellets were bead-beated with MP Biomedicals FastPrep-24 Classic at 4.5 m/s, 3x times, for 45s each. Between cycles, samples were cooled on ice for at least 5 min. After centrifugation at 13.000 g for 10 minutes at 4°C, supernatants were transferred to filter tubes (Spin-X centrifuge tube filter,

Costar: 8160) and centrifuged at 5000 g for 5 min at 4°C. Filtered supernatants were then stored at -80°C.

Metabolomics was performed using an Agilent II LC system coupled to a 6546 high-resolution quadrupole time-of-flight mass spectrometer as described (19). In brief, samples (2 μL) were injected onto a Diamond Hydride type C column (150 X 2.1 mm, 4 μm) and separated using a 0.4-mL/min gradient of water with 0.2% formic acid in acetonitrile with 0.2% formic acid (B) (0–2 min, 85% B; 3–5 min, 80% B; 6–7 min, 75% B; 8–9 min, 70% B; 10–11 min, 50% B; 11–14 min, 20% B; 14–24 min, 5% B; followed by 10 min re-equilibration at 85% B). Mass spectra were recorded from m/z 50–1200 in the positive or negative ionization mode at 1 spectrum/s. Features corresponding to the reported analytes were identified based on accurate mass and retention time. Peak areas were integrated using MassHunter Profinder Software (Agilent), which was also used to assess $^{13}C_6$-labeling and correction for natural $^{13}C_6$ abundance.

In vivo **efficacy testing in mice**

The experimental project was favorably evaluated by the ethics committee n°005 Charles and clearance was given by the French Ministry of Education and Research under the number APAFIS #46959-202401191152302. The animal facility received the authorization to carry out animal experiments (license number D-75-13-08). Female BALB/C mice were infected with H37Rv *Mtb* via the intranasal route infection at an inoculum of 1 × 10^5 CFU per mouse. Drug treatment was given at 3 days post-infection. All the drugs were formulated in 50% KLEPTOSE® HP Oral grade Hydroxypropyl β-cyclodextrin (Roquette) (pH=3.5) and administered via gavage for 6 days/week for the 1st week and 5 days/week for the 2nd week in the total of 14 days. BDM91465, telacebec and Clofazimine were utilized in single drug treatment or in combination at 60 mg/kg, 10 mg/kg and 20 mg/kg, respectively. Mice were then sacrificed and bacterial load in the lung was determined by CFU plating.

# - Supplemental Tables -

Table S1: The antibiotic activity represented by minimal inhibitory concentration (MIC) of TriSLa BDM91465 on H37Rv-GFP *Mtb* when grown in MMAT-base media containing single or double carbon combination, as well as the final selected $MMAT_{GluFA}$ and $MMAT_{GluGlyChl}$ media. MIC_{90} were defined as the amount of compound that resulted in the detection of less than 10 % of the GFP signal compared to no antibiotic control. Data are the averages of three independent assays, with ranges indicated. In addition to antibiotic susceptibility, the relative bacteria growth is shown for each media, relative to media containing glucose and fatty acids (mean ± SD).

		**BDM91465 MIC_{90} (µM)** ***(percentage growth compared to Glucose + Oleic acid)***		
		**2nd Carbon Source**		
		**None**	**0.2 % Glucose**	**0.2 % Glycerol**
**1st Carbon Source**	**0.2 % Glucose**	>10 *(32±8%)*		
	**0.2 % Glycerol**	>10 *(60±20%)*	>10 *(98±24%)*	
	**0.2 % Acetate**	>10 *(53±12%)*	>10 *(72%±44%)*	>10 *(171±37%)*
	**0.2 % Propionate**	>10 *(15±3%)*	>10 *(41%±5%)*	10 *(86±13%)*
	**0.02% Cholesterol**	>10 *(23±12%)*	>10 *(53%±6%)*	>10 *(143±23%)*
	**0.2 % Pyruvate**	0.37 *(344±95%)*	0.040 *(288%±140%)*	0.022 (0.013-0.040) *(587±43%)*
	**200 µm Palmitic Acid**	0.18 (0.040-0.37) *(107±53%)*	0.086 (0.013-0.12) *(159±52%)*	0.059 (0.013-0.12) *(225±35%)*
	**200 µm Oleic Acid**	0.031 (0.013-0.040) *(106±18%)*	0.031 (0.013-0.040) ***(100%)***	0.031 (0.013-0.04) *(53±13%)*
$MMAT_{GluGlyChl}$	**0.2 % Glucose 0.2 % Glycerol 0.02% Cholesterol**	>10 *(164±35%)*		
$MMAT_{GluFA}$	**0.2 % Glucose 200 µM Oleic Acid 0.0003% Catalase**	0.022 (0.013-0.040) *(288±16%)*		

Table S2: The antibiotic activity represented by minimal inhibitory concentration (MIC) of bedaquiline on H37Rv-GFP *Mtb* when grown in MMAT-base media containing single or double carbon combination, as well as the final selected $MMAT_{GluFA}$ and $MMAT_{GluGlyChl}$ media. MIC_{90} were defined as the amount of compound that resulted in the detection of less than 10 % of the GFP signal compared to no antibiotic control. Data are the averages of three independent assays, with ranges indicated.

		**Bedaquiline MIC_{90} (μM)** *(percentage growth compared to Glucose + Oleic acid)*		
		**2nd Carbon Source**		
		**None**	**0.2 % Glucose**	**0.2 % Glycerol**
**1st Carbon Source**	**0.2 % Glucose**	0.62 (0.37-1.11)		
	**0.2 % Glycerol**	0.86 (0.37-1.11)	0.62 (0.37-1.11)	
	**0.2 % Acetate**	0.37	0.21 (0.12-0.37)	0.37
	**0.2 % Propionate**	0.37	0.21 (0.12-0.37)	0.12
	**0.02% Cholesterol**	0.86 (0.37-1.11)	0.62 (0.37-1.11)	0.37
	**0.2 % Pyruvate**	0.12	0.29 (0.12-0.37)	0.37
	**200 μm Palmitic Acid**	0.29 (0.12-0.37)	0.62 (0.37-1.11)	0.26 (0.04-0.37)
	**200 μm Oleic Acid**	0.37	0.37	0.06 (0.01-0.12)
$MMAT_{GluGlyChl}$	**0.2 % Glucose<br>0.2 % Glycerol<br>0.02% Cholesterol**	0.37		
$MMAT_{GluFA}$	**0.2 % Glucose<br>200 μM Oleic Acid<br>0.0003% Catalase**	0.37		

Table S3. BDM91465 susceptibility of *Mtb* and isogenic strains lacking *whiB3* and *whiB7*. Minimal inhibitory concentration (MIC) of BDM91465 was measured on WT H37Rv, and isogenic mutants H37RvΔ*whiB3* and H37RvΔ*whiB7* growing in both $MMAT_{GluFA}$ and $MMAT_{GluGlyChl}$ by Resazurin microtiter assay following 7 day exposure. Data are the average MICs of two independent experiments.

	Minimal inhibitory concentration (MIC) (µM)					
	WT H37Rv		H37RvΔ*whiB3*		H37RvΔ*whiB7*	
	$MMAT_{GluFA}$	$MMAT_{GluGlyChl}$	$MMAT_{GluFA}$	$MMAT_{GluGlyChl}$	$MMAT_{GluFA}$	$MMAT_{GluGlyChl}$
**BDM91465**	0.062	>0.5	0.031	>0.5	0.046 (0.031-0.062)	>0.5

Table S4. BDM91465 susceptibility of *Mtb* and isogenic strains with loss of function mutation in Rv0678 (N70D). Minimal inhibitory concentration (MIC) of BDM91465 on WT H37Rv-GFP and OH190 RC20.1-GFP (isogenic strain carrying Rv0678(N70D)) with each strain harboring GFP plasmid, were were measured in $MMAT_{GluFA}$. Data are the average of three independent experiments with the concentration range in the brackets.

	Minimal inhibitory concentration (MIC)	
	**H37Rv-GFP**	**OH190 RC20.1-GFP**
**BDM91465 (µM)**	0.020 (0.015-0.031)	0.041 (0.031-0.062)
**BDQ (µM)**	0.083 (0.062-0.125)	0.208 (0.125-0.25)
**CFZ (µM)**	4	8
**INH (µg/ml)**	0.063	0.063

Table S5. Antibiotic susceptibility of *Mtb* lacking NDH-1 (H37RvΔ*nuoF*), NDH-2 (H37RvΔ*ndh-2*) and succinate dehydrogenase (H37RvΔ*sdh*). The minimal inhibitory concentration (MIC) of BDM91645, bedaquiline (BDQ), clofazimine (CFZ), telacebec (Q203), rifampicin (RIF) and rotenone (ROT) was determined on WT H37Rv, H37RvΔ*nuoF*, H37RvΔ*ndh*Δ*ndhA* (Δ*ndh-2*) and H37RvΔ*sdh* strains (all harboring a GFP reporter plasmid). *Mtb* viability was detected by GFP fluorescence (Excitation 485 nm, Emission 510 nm) after 7 day incubation with the antibiotics. Data are the average of three independent experiments with the range in MIC data indicated in brackets.

	Antibiotic MIC in indicated strains and media							
	H37Rv-GFP		H37Rv-Δ*ndh-2*		H37Rv-Δ*nuoF*		H37Rv-Δ*sdh1*	
	$MMAT_{GluFA}$	$MMAT_{GluGlyChl}$	$MMAT_{GluFA}$	$MMAT_{GluGlyChl}$	$MMAT_{GluFA}$	$MMAT_{GluGlyChl}$	$MMAT_{GluFA}$	$MMAT_{GluGlyChl}$
**BDM91465 (µM)**	0.026 (0.015-0.031)	>1	No Growth	>1	0.0078	0.026 (0.015-0.031)	0.016	>1
**BDQ (µM)**	0.29 (0.125-0.5)	0.17 (0.125-0.25)	No Growth	0.125	0.42 (0.25-0.5)	0.25	0.5	0.25
**CFZ (µM)**	3.3 (2.5-5)	3.3 (2.5-5)	No Growth	3.3 (2.5-5)	4.2 (2.5-5)	5	5	4.2 (2.5-5)
**Q203 (µM)**		0.15 (0.063-0.25)	No Growth	0.0078		0.42 (0.25-0.5)		0.21 (0.125-0.25)
**RIF (µg/mL)**	0.072 (0.031-0.125)	0.125	No Growth	0.10 (0.063-0.125)	0.063	0.21 (0.125-0.25)	0.083 (0.063-0.125)	0.21 (0.125-0.25)
**ROT (µM)**	>100	>100	No Growth	8.3 (6.3-12.5)	>100	>100	>100	>100

Table S6. List of *Mtb* strains used or generated in this study**.** Strains used in the main manuscript are highlighted in **bold**, and the names used in the text are indicated in parentheses**.**

**Strain**	**Descriptions**	**Drug resistance marker**
H37Rv WT	Laboratory reference strain	-
H37Rv pJK-D6 (H37Rv-GFP)	H37Rv transformed with plasmid pJK-D6 (GFP)	Kan^r
H37Rv RC1.1 pJK-D6	H37Rv RC1.1 (strain with *ndh* mutation (t1207c : Tyr403His) derived from study (6)) transformed with plasmid pJK-D6 (GFP)	Kan^r
H37Rv pKM444	H37Rv transformed with plasmid pKM444 (ORBIT system)	Kan^r
H37Rv OH190 RC20.1-GFP	H37Rv OH190 RC20.1 (strain with *rv0678* mutation (a208g : Asn70Asp) derived from study (20)) transformed with plasmid pJK-D6 (GFP)	Kan^r
H37Rv pKM444 Δ*nuoF*::pKM464	H37Rv pKM444 with the plasmid pKM464 inserted at *nuoF* (*Rv3150*, knock-out)	Kan^r, Hyg^r
H37Rv pKM512 Δ*nuoF*::attB	H37Rv pKM444 Δ*nuoF*::pKM464 transformed with plasmid pKM512, that allows for pKM444 curing and excision of pKM464, leaving an attP site inserted at *nuoF*	Zeo^r
H37Rv Δ*nuoF*::attB	H37Rv pKM512 Δ*nuoF*::attB subcultured in presence of sucrose, resulting in loss of pKM512	-
H37Rv Δ*nuoF*::attB pJK-D6 (H37Rv Δ*nuoF*)	H37Rv Δ*nuoF*::attB transformed with plasmid pJK-D6 (GFP)	Kan^r
H37Rv pKM444 Δ*sdh1*::pKM464	H37Rv pKM444 with the plasmid pKM464 inserted at *sdh1* (*Rv0248c*, knock-out)	Kan^r, Hyg^r
H37Rv pKM512 Δ*sdh1*::attB	H37Rv pKM444 Δ*sdh1*::pKM464 transformed with plasmid pKM512, that allows for pKM444 curing and excision of pKM464 leaving an attP site inserted in *sdh1*	Zeo^r
H37Rv Δ*sdh1*::attB	H37Rv pKM512 Δ*sdh1*::attB subcultured in presence of sucrose, resulting in loss of pKM512	-
H37Rv Δ*sdh1*::attB pJK-D6 (H37Rv Δ*sdh1*)	H37Rv Δ*sdh1*::attB transformed with plasmid pJK-D6 (GFP)	Kan^r
H37Rv pKM444 Δ*ndh*::pKM464	H37Rv pKM444 with the plasmid pKM464 inserted at *ndh* (*Rv1854c*, knock-out)	Kan^r, Hyg^r
H37Rv pKM512 Δ*ndh*::attB	H37Rv pKM444 Δ*ndh*::pKM464 transformed with plasmid pKM512, that allows for pKM444 curing and excision of pKM464 leaving an attP site inserted in *ndh*	Zeo^r
H37Rv Δ*ndh*::attB	H37Rv pKM512 Δ*ndh*::attB subcultured in presence of sucrose, resulting in loss of pKM512	-
H37Rv Δ*ndh*::attB pCRISPR-ndhA	H37Rv Δ*ndh*::attB transformed with plasmid pCRISPR-ndhA, integrated at L5-attB site	Kan^r
H37Rv Δ*ndh*::attB ndhA* L5-attB::pCRISPR-ndhA		Kan^r

	H37Rv Δ*ndh*::attB pCRISPR-ndhA containing a 14 bp deletion in *ndhA* (*Rv0392c*) (ΔGCTGATCCTGCGCC), causing a frameshift mutation	
H37Rv Δ*ndh*::attB ndhA* L5-attB::empty	H37Rv Δ*ndh*::attB ndhA* L5-attB::pCRISPR-ndhA transformed with plasmid L5-attB::empty and pBS-Int, resulting in replacement of pCRISPR-ndhA at the L5-attB site	Apr^r
H37Rv Δ*ndh*::attB ndhA* pJK-D6 L5-attB::empty<br>(H37Rv Δ*ndh2*)	H37Rv Δ*ndh*::attB ndhA* L5-attB::empty transformed with plasmid pJK-D6 (GFP)	Kan^r
H37Rv pKM444 Δ*whiB3*::pKM464<br>(H37Rv Δ*whiB3*)	H37Rv pKM444 with the plasmid pKM464 inserted at *whiB3* (*Rv3416*, knock-out)	Kan^r, Hyg^r
H37Rv pKM444 Δ*whiB7*::pKM464<br>(H37Rv Δ*whiB7*)	H37Rv pKM444 with the plasmid pKM464 inserted at *whiB7* (*Rv3197a*, knock-out)	Kan^r, Hyg^r

Table S7. List of plasmids used in this study.

Plasmid name	Description	Drug resistance marker	Reference
pKM444	PTet-Che9c RecT-Bxb1 Int, TetR, oriE, oriM	Kan^r, Cam^r	(4)
pKM464	Bxb1 attB, oriE	Hyg^r, Cam^r	(4)
pKM512	PTet-Bxb1 GP47-Int, SacRB, TetR, oriE, oriM	Zeo^r, Cam^r	(4)
pJK-D6	P$_{pJK\text{-}D6}$-*egfp,* oriC, oriM	Kan^r	(1)
pCRISPRx-Sth1Cas9-L5	Sth1 Cas9, TetR, L5 Int, L5 attP, oriE	Kan^r	(5)
pCRISPR-ndhA	Sth1 Cas9, sgRNA-ndhA, TetR, L5 Int, L5 attP, oriE. (Generated from pCRISPRx-Sth1Cas9-L5 by cloning the ndhA-specific sgRNA into the BsmBI restriction site)	Kan^r	This study
pBS-Int	L5 Int, oriE	Amp^r	(21)
L5 attB::Pleft* mWasabi	P$_{left*}$-mWasabi, L5 attP, oriE	Kan^r	(22)
L5 attB::empty	L5 attP, oriE (Derived from L5 attB::Pleft*-mWasabi, from which the Pleft*-mWasabi cassette was removed by HindIII digestion, and the Kanr marker was replaced with Aprr marker)	Apr^r	This study

Table S8. List of oligonucleotides used in this study.

Name	Sequence (5'→3')
**ORBIT oligos for gene knock-out**	
**ORBIT_ndh**	TCCAGCCGCTGCTGTACCAAGTGGCCACCGGGATTATCTCCGAGGGAGAAATCGCTCCG CCGACCCGGGTGGTTTGTACCGTACACCACTGAGACCGCGGTGGTTGACCAGACAAACC TTCGGCAACGACCATTTCGCCGAATTCGCACCCGGCATGAAGTCCATCGACGACGCGTT GGAGTTGCGTG
**ORBIT_ndh_UC**	CGGTCGGGTTGACTACCGTAG
**ORBIT_ndh_DC**	GGCGTCAAGCAGAATCACCC
**ORBIT_nuoF**	ATCAGCATCGTCAAGGATTCCGGGTTACGCGGACGCGGCGGCGCGGGCTTTGCCACCG GGACCAAGTGGTGGTTTGTACCGTACACCACTGAGACCGCGGTGGTTGACCAGACAAAC CATCCGCGCCCATCACGCGTTCGTCTACGTACGCGGTGAGGTGGTGCCGGTATTGCGCC GGCTGCACAACG
**ORBIT_nuoF_UC**	GCAAACATCGCGCATCCTGG
**ORBIT_nuoF_DC**	GGCGATTTCTCGCTGCCCATC
**ORBIT_sdh1**	TGGAAAGTTCCTGAACAACTGGCGTATGGCCGAGCTGCACGCCAAGGAGGCGCCGGACC GGGTCTGGGAGGGTTTGTACCGTACACCACTGAGACCGCGGTGGTTGACCAGACAAACC AGCAGGAGGACCACGCCGAGCTCGGCGACTACGAAGCGCGGATCAAAGTGTTCGCCGA ATGCACCATCAC
**ORBIT_sdh1_UC**	GGCCAGTGGCAGCATCACAG
**ORBIT_sdh1_DC**	CCGCGAACACCCTCAGTGAC
**ORBIT_whiB3**	CAGTTGCACAACTCGCGCGTCCGCGAACCCAACCTCCACTTGGGCGTGTCGTGCAGAGA GGGAATCAGCAGGTTTGTACCGTACACCACTGAGACCGCGGTGGTTGACCAGACAAACC GCCGCGCGAGCAGACGCTAAAGCCCCCGCACGCTCGGCGTGTCGGGGGCTTTTGCGTC TGCTGACCGGAG
**ORBIT_whiB3_UC**	CGCAGATGAACGCAGAG
**ORBIT_whiB3_DC**	GAAGATCACGACCATC
**ORBIT_whiB7**	CCCGGGTTTCGCCGAGCCCGACGCGATCGTCGTCTGAGCCGGCTCGCGCCGGCGGGCG CACCATCGCGGGGGTTTGTACCGTACACCACTGAGACCGCGGTGGTTGACCAGACAAAC CGTCCTGTTTCACCTGCTTCCTGGTCTGGTGGCGGTTCTTCGAAAGTGATCCGGACCAGG GATGCTGCGGT
**ORBIT_whiB7_UC**	GTTCGCGAGATCGACGAGATGCTG
**ORBIT_whiB7_DC**	GATTGACAACAACGCATTTGGTGTC
**ORBIT-attB-F**	CGGTGGTTGACCAGACAAACC
**ORBIT-attB-R**	GGTTTGTCTGGTCAACCACCG
**Oligos for CRISPR-mediated ndhA deletion**	
**sgRNA-ndhA_F**	GGGAACCCGGCTGATCCTGCGCCGGCA
**sgRNA-ndhA_R**	AAACTGCCGGCGCAGGATCAGCCGGGT
**ndhA_seq_F**	GTCGACATCACGCTGATCTCC
**ndhA_seq_R**	CCGGGCACGAACATCAGATC
**ndhA_seq_R2**	TCCATCAATTTCGACGTGAC
**Oligos for amplifying the Apr-resistance cassette**	
**AprR_F**	GTGTAGGCTGGAGCTGCTTC
**AprR_R**	CATATGAATATCCTCCTTAG

Table S9. Quality control of CRISPRi library

[1] Missing sgRNA number and Gini Index were extracted directly from the Mageck count function output
[2] Skew ratio was calculated on raw counts, excluding missing sgRNAs (to avoid division by zero) and empty plasmid counts (noted as >empty in RLC12) as these counts are 1000-fold higher than any sgRNA count and massively bias the skew ratio.

Sample	Reads	Mapped	Percentage mapped	Total sgRNAs in RLC12	Missing sgRNAs[1]	Gini Index[1]	Skew ratio[2]
$MMAT_{GluGlyChl}$_DMSO_1	62894468	55889640	0.8886	96700	2085	0.1525	34.639
$MMAT_{GluGlyCh}$_DMSO_2	62725170	55534564	0.8854	96700	1905	0.151	34.889
$MMAT_{GluGlyCh}$_DMSO_3	59190283	52577334	0.8883	96700	2046	0.155	36.875
$MMAT_{GluGlyCh}$_TriSLa_1	62605070	55723836	0.8901	96700	2320	0.1663	47.481
$MMAT_{GluGlyCh}$_TriSLa_2	64004441	56994328	0.8905	96700	2471	0.174	59.100
$MMAT_{GluGlyCh}$_TriSLa_3	61738707	54959454	0.8902	96700	2323	0.1768	65.400
$MMAT_{GluFA}$_DMSO_1	83166554	72669365	0.8738	96700	232	0.1052	17.429
$MMAT_{GluFA}$ _DMSO_2	135814607	118753906	0.8744	96700	252	0.1149	26.798
$MMAT_{GluFA}$ _DMSO_3	62564915	54824148	0.8763	96700	350	0.1163	20.143
$MMAT_{GluFA}$ _TriSLa_1	65534998	58898404	0.8987	96700	222	0.09141	8.670
$MMAT_{GluFA}$ _TriSLa_2	70719835	64600988	0.9135	96700	250	0.1054	10.633
$MMAT_{GluFA}$ _TriSLa_3	93804771	86454963	0.9216	96700	353	0.104	11.688

Table S10. Targeted CRISPRi chemical genomics data analysis (Log_2FC and logFDR) of the vulnerability of *Mtb* genes involved in cell wall biosynthesis (targets of current anti-tuberculosis antibiotics) towards BDM91465 in $MMAT_{GluFA}$ and $MMAT_{GluGlyChl}$.

Cell wall biosynthesis gene	Drug targeting gene product	Medium	Log Fold change (Log_2FC)[a]	Log False discovery rate (LogFDR)
inhA	Isoniazid<br>Ethionamide<br>Pyridomycin	$MMAT_{GluFA}$	1.79	5.1
		$MMAT_{GluGlyChl}$	0.99	0.36
dprE1	PBTZ169	$MMAT_{GluFA}$	2.21	5.1
		$MMAT_{GluGlyChl}$	1.08	0.1
embB	Ethambutol	$MMAT_{GluFA}$	1.48	5.1
		$MMAT_{GluGlyChl}$	1.05	0.08

[a] Positive Log fold change indicates enrichment of gene in CRISPRi library in the presence of BDM91465

# - Supplemental Figures -

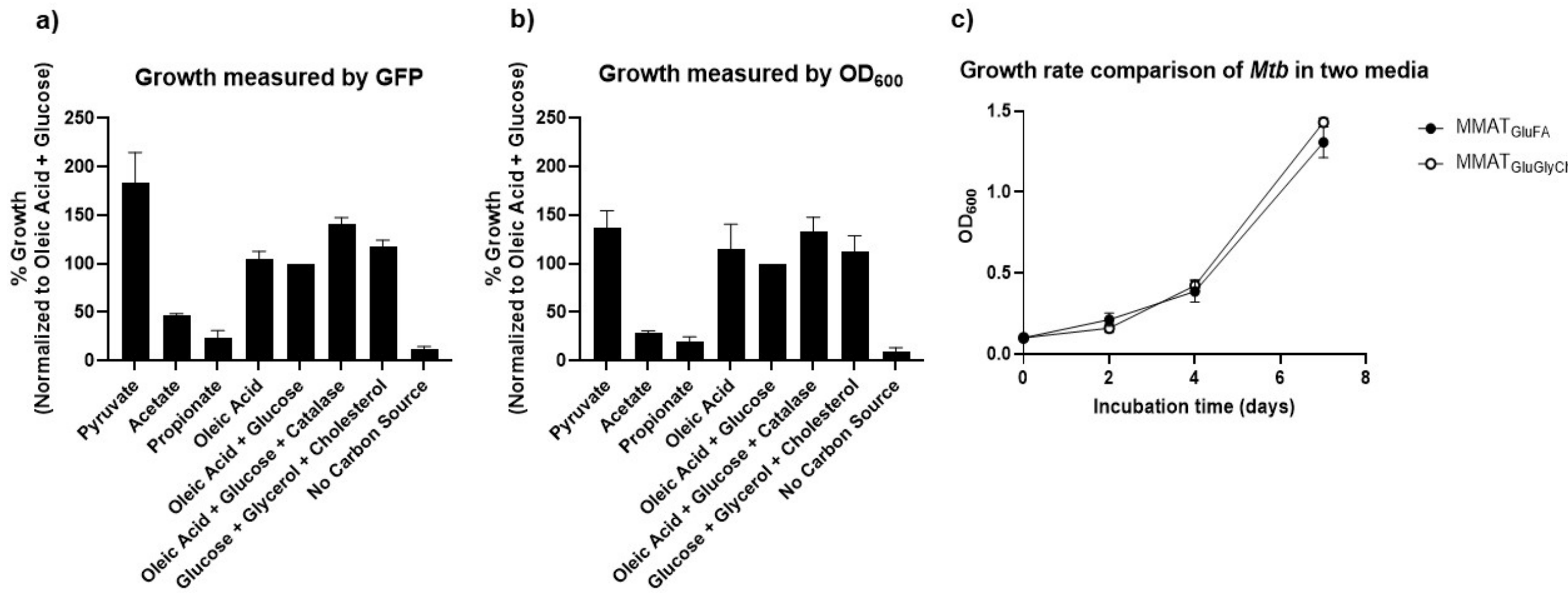

Figure S1. Growth rate of *Mtb* in MMAT media supplemented with selected carbon sources. *Mtb* was initially grown in Middlebrook 7H9-OADC-Glycerol-Tyloxapol to mid-log phase. Bacteria was then diluted to OD_{600}=0.001 in MMAT media with selected carbon sources [Pyruvate (0.2% Pyruvate); Acetate (0.2% Acetate); Propionate (0.2% Propionate); Oleic Acid (200 µM Oleic Acid); Oleic Acid + Glucose (200 µM Oleic Acid + 0.2% Glucose); Oleic Acid + Glucose + Catalase (200 µM Oleic Acid + 0.2% Glucose + 0.0003% Catalase: $MMAT_{GluFA}$) and Glucose + Glycerol + Cholesterol (0.2% Glucose + 0.2% Glycerol + 0.02% Cholesterol: $MMAT_{GluGlyChl}$)]. Growth of H37Rv-GFP was measured after 7 days of incubation by both (**a**) GFP and (**b**) OD_{600}. Which was normalized to corresponding growth in $MMAT_{GluFA}$ (without catalase) (**c**) The bacterial growth rate of H37Rv in $MMAT_{GluFA}$ and $MMAT_{GluGlyChl}$ was also monitored over 7 days by OD_{600} measurement demonstrating comparable growth. Values are the mean and SD of three independent biological replicates.

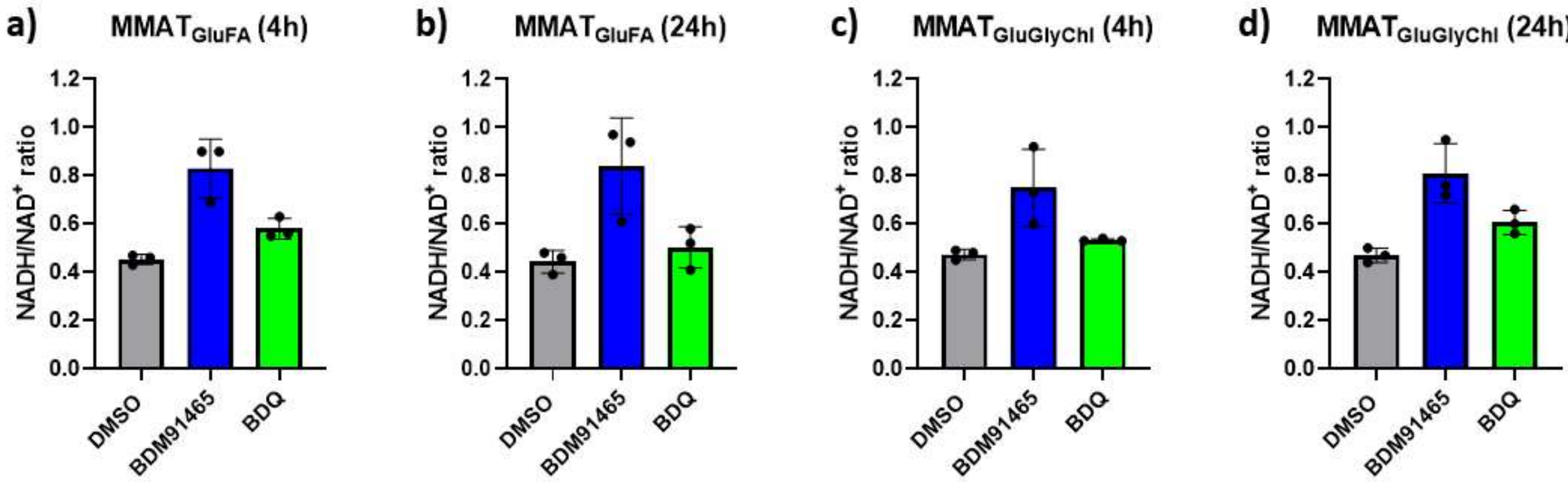

Figure S2: Impact of BDM91465 on the NADH/NAD⁺ ratio in *Mtb* grown in $MMAT_{GluFA}$ and $MMAT_{GluGlyChl}$. Log phase H37Rv-GFP growing in $MMAT_{GluFA}$ (**a, b**) or $MMAT_{GluGlyChl}$ (**c, d**) were treated with 0.4 µM BDM91465 or 2 µg/mL BDQ and NADH/NAD⁺ ratios measured following 4 h (**a, c**) or 24 h (**b, d**) exposure. Values are the mean and SD of three independent experiments.

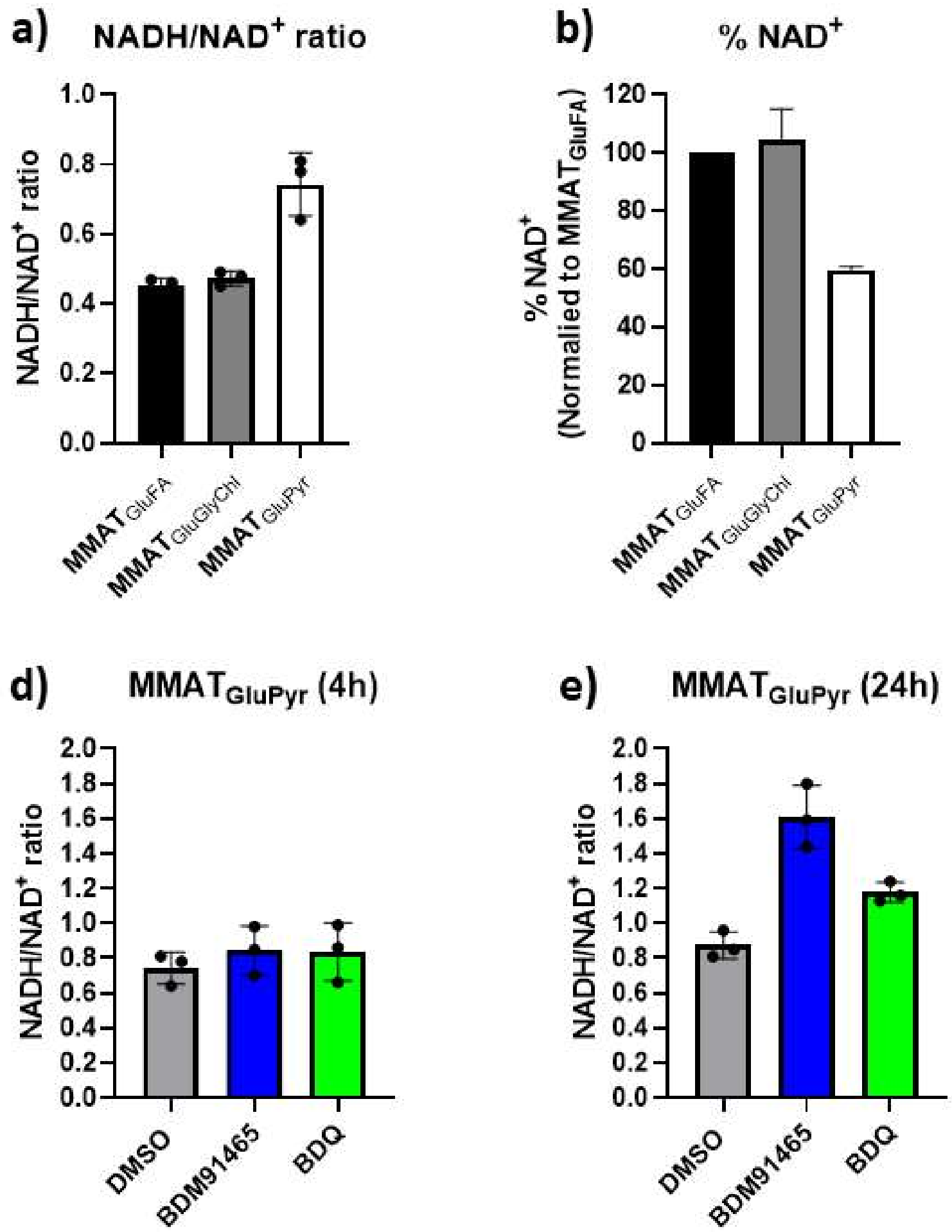

Figure S3: Impact of BDM91465 on NADH/NAD⁺ ratio in *Mtb* growing in MMAT media with pyruvate. H37Rv-GFP was grown in $MMAT_{GluPyr}$ (MMAT-based media with 0.2% glucose and 0.2% pyruvate) to mid-log phase. **(a)** H37Rv-GFP NADH/NAD⁺ ratio was assessed in $MMAT_{GluFA}$, $MMAT_{GluGlyChl}$ (like in **Fig. S2**) and in $MMAT_{GluPyr}$, in the absence of antibiotics, with associated bacterial **(b)** NAD+ and **(c)** NADH measured (normalizing to the amounts measured in $MMAT_{GluFA}$). In addition, the NADH/NAD⁺ ratio of H37Rv-GFP grown in $MMAT_{GluPyr}$ was measured following treatment with 0.4 µM BDM91465 or 2 µg/mL BDQ at **(d)** 4 h or **(e)** 24 h incubation. Values are the mean and SD of three independent experiments.

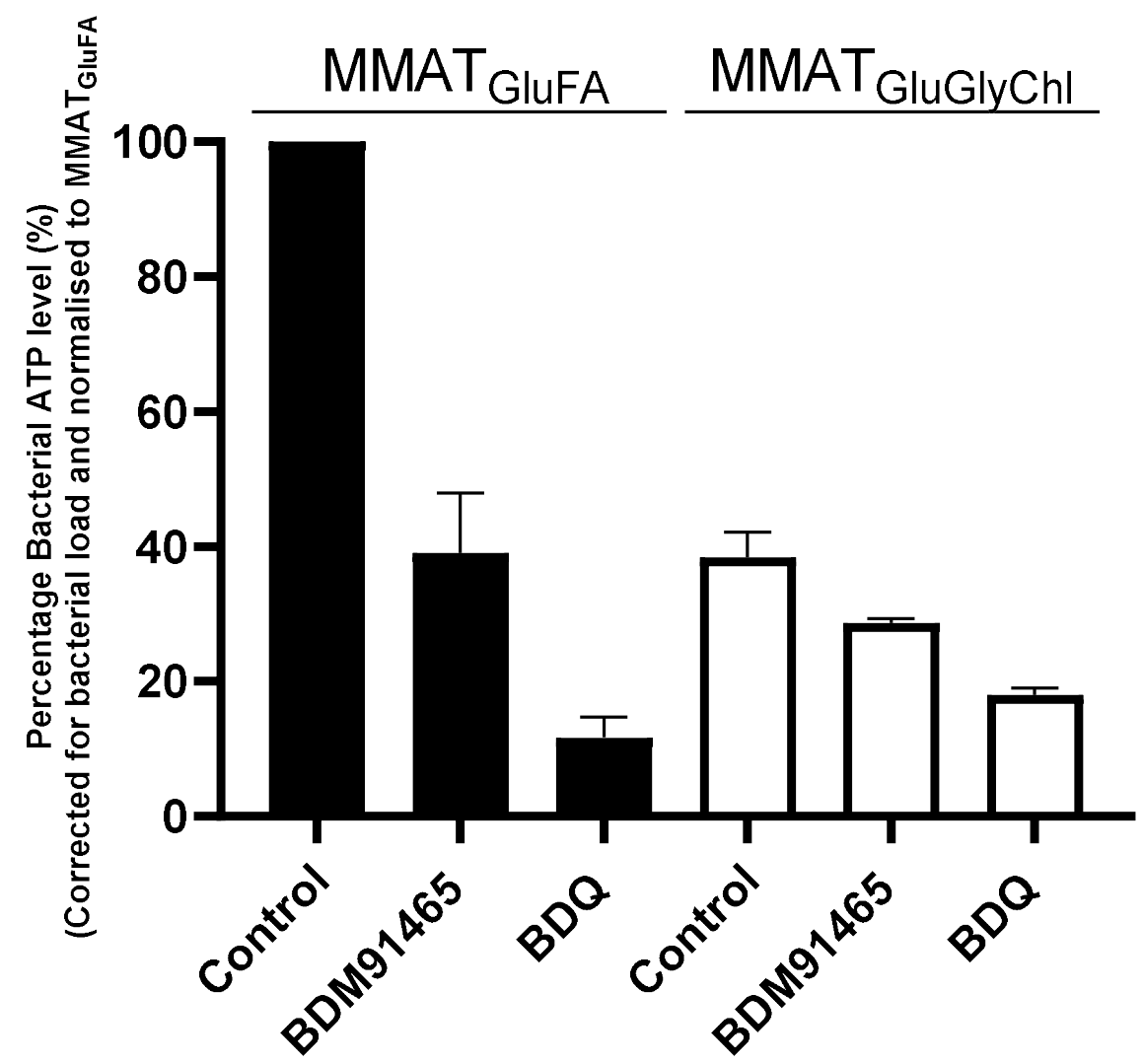

Figure S4: The impact of BDM91465 on *Mtb* ATP level when grown in $MMAT_{GluFA}$ or $MMAT_{GluGlyChl}$. H37Rv-GFP was grown in $MMAT_{GluFA}$ or $MMAT_{GluGlyChl}$ to mid-log phase. Bacteria were then treated with 1 µM BDM91465 or 1 µM BDQ for 24 h before measuring bacterial growth (by GFP fluorescence) and bacterial ATP (RLU by Bactiter-Glo). Bacterial ATP concentrations are normalized to bacterial concentration (RLU by Bactiter-Glo/Bacterial GFP), and expressed relative to basal values obtained for bacteria in $MMAT_{GluFA}$ (no antibiotic). Black columns represent values obtained in $MMAT_{GluFA}$ while white columns represent data from $MMAT_{GluGlyChl}$.. Values are the mean and SD of three independent experiments.

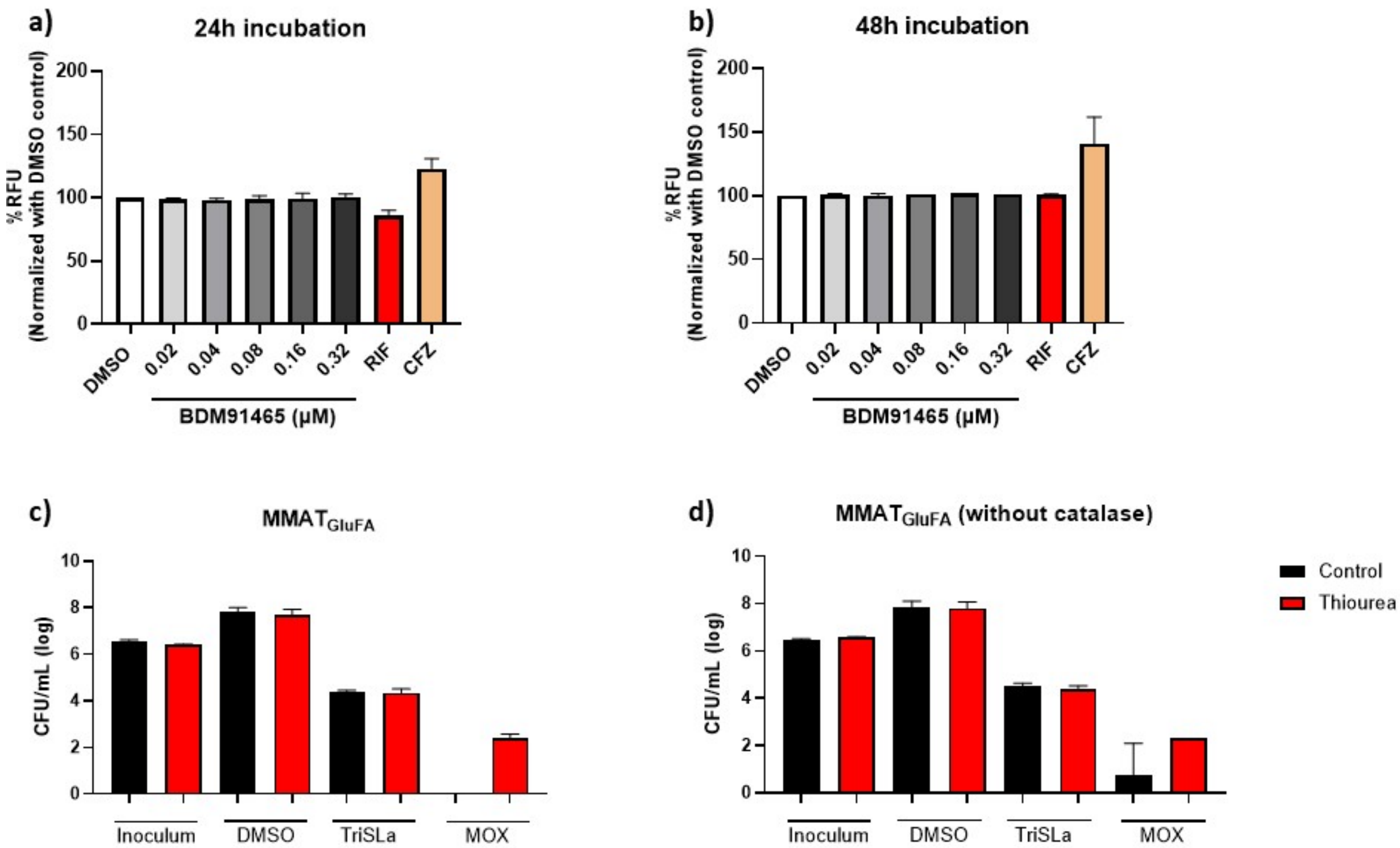

Figure S5. TriSLa does not induce detectible ROS production in *Mtb*. Using the DCFDA assay (**a, b**) the generation of ROS was monitored in H37Rv growing in MMAT$_{GluFA,}$ following a (**a**) 24-h and (**b**) 48-h incubation with increasing concentrations of TriSLa BDM91465, 1 µg/mL rifampicin (RIF) or 8 µg/mL clofazimine (CFZ). Data showed no significant induction of ROS by TriSLa exposure, though this was observed for the control CFZ. As a complement, the bactericidal activity of 0.4 µM TriSLa BDM91465 (TriSLa) and 1 µg/mL moxifloxacin (MOX) were measured in the presence and absence of the ROS scavenger; thiourea (10 mM), in **(c)** MMAT$_{GluFA}$ with catalase and in **(d)** MMAT$_{GluFA}$ without catalase. Bacterial viability was measured after 2-week compound exposure by CFU counting, and shows that thiourea does not impact TriSLa activity, but does impact the activity of moxifloxacin. All data are represented as the mean and SD of three independent experiments.

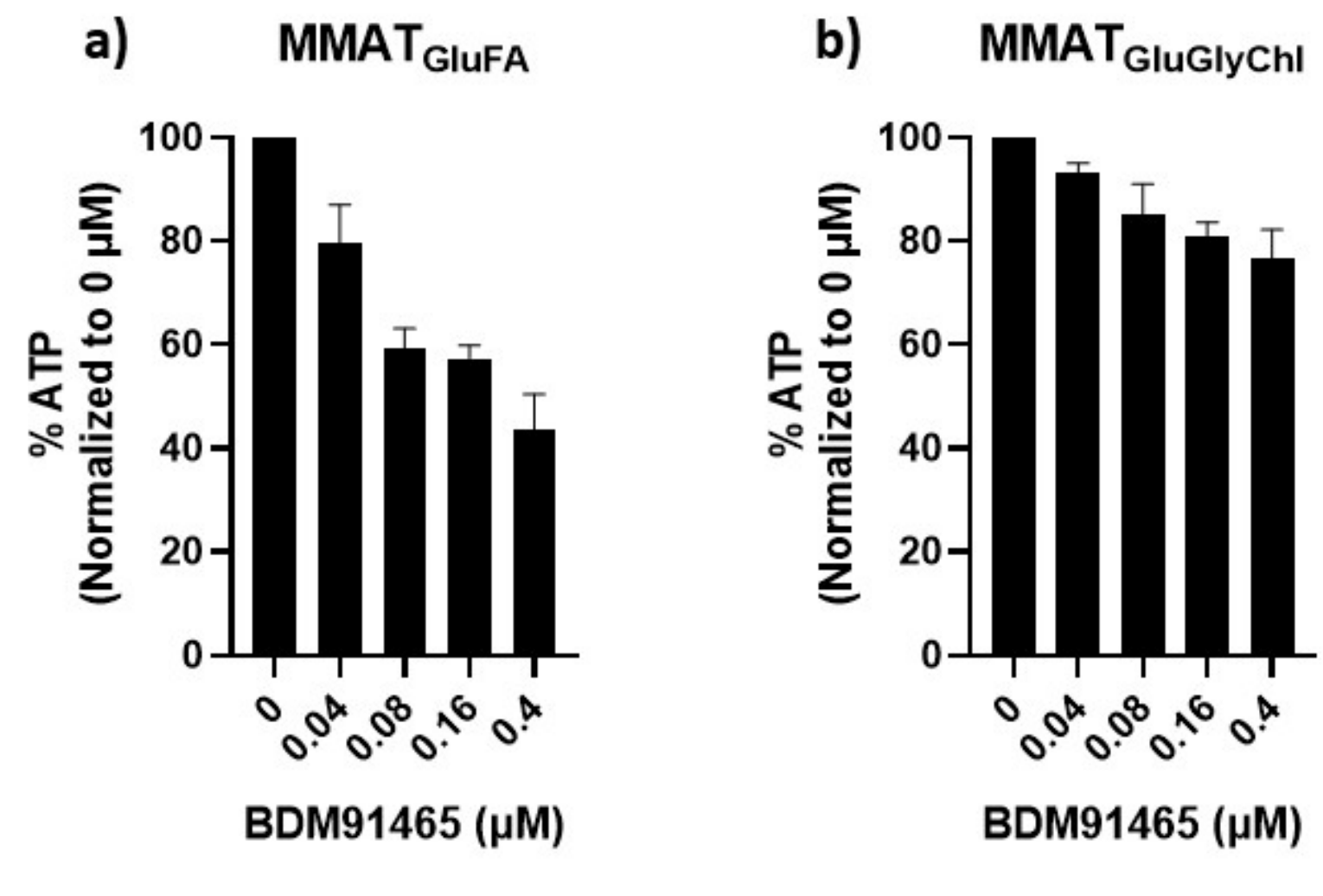

Figure S6. Early impact of BDM91465 on *Mtb* ATP production in two media conditions. *Mtb* H37Rv-GFP was grown in (**a**) MMAT$_{GluFA}$ or (**b**) MMAT$_{GluGlyChl}$ to mid-log phase. Bacteria were then treated with the indicated range of BDM91465 for 4 h before measuring bacterial ATP levels (by Bactiter-Glo assay). These data were obtained under conditions that mirror the RNAseq transcriptome analysis and demonstrate that 4-h incubation with 0.16 µM BDM91465 (used for RNAseq) was a sufficient exposure to lead to changes in bacterial ATP. Data are the mean and SD of three independent experiments.

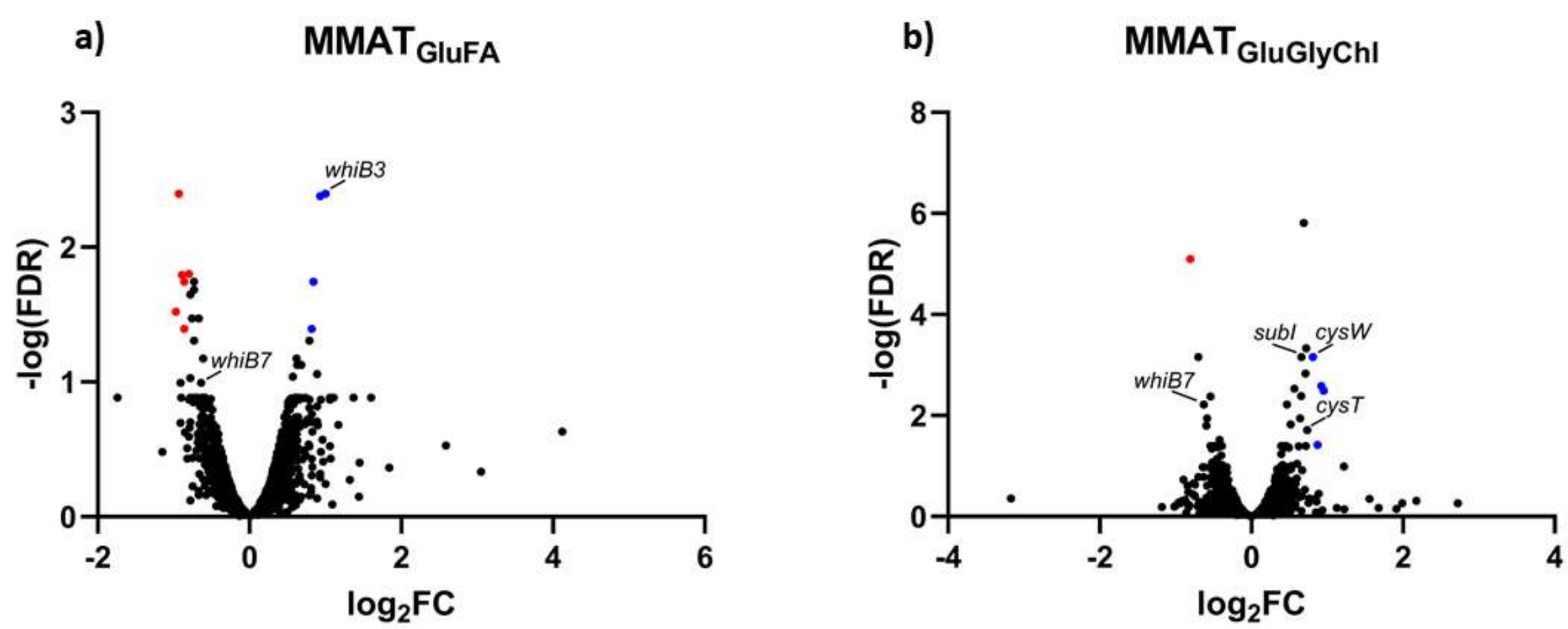

Figure S7. Transcriptomic analysis of *Mtb* treated with TriSLa BDM91465 in different media conditions. Volcano plots depict the differentially expressed genes in H37Rv *Mtb* following a 4-h exposure to 0.16 µM TriSLa BDM91465 compared to the DMSO-treated controls. Analysis was performed both (**a**) MMAT$_{GluFA}$ and in (**b**) MMAT$_{GluGlyChl}$. Significantly downregulated genes (log2FC ≤ -0.8 and FDR ≤ 0.05) are reflected in red and significantly upregulated genes (log2FC ≥ 0.8 and FDR ≤ 0.05) are reflected in blue. Data are from three independent experiments. Individual values for these RNAseq experiments are tabulated in **Data set S1**.

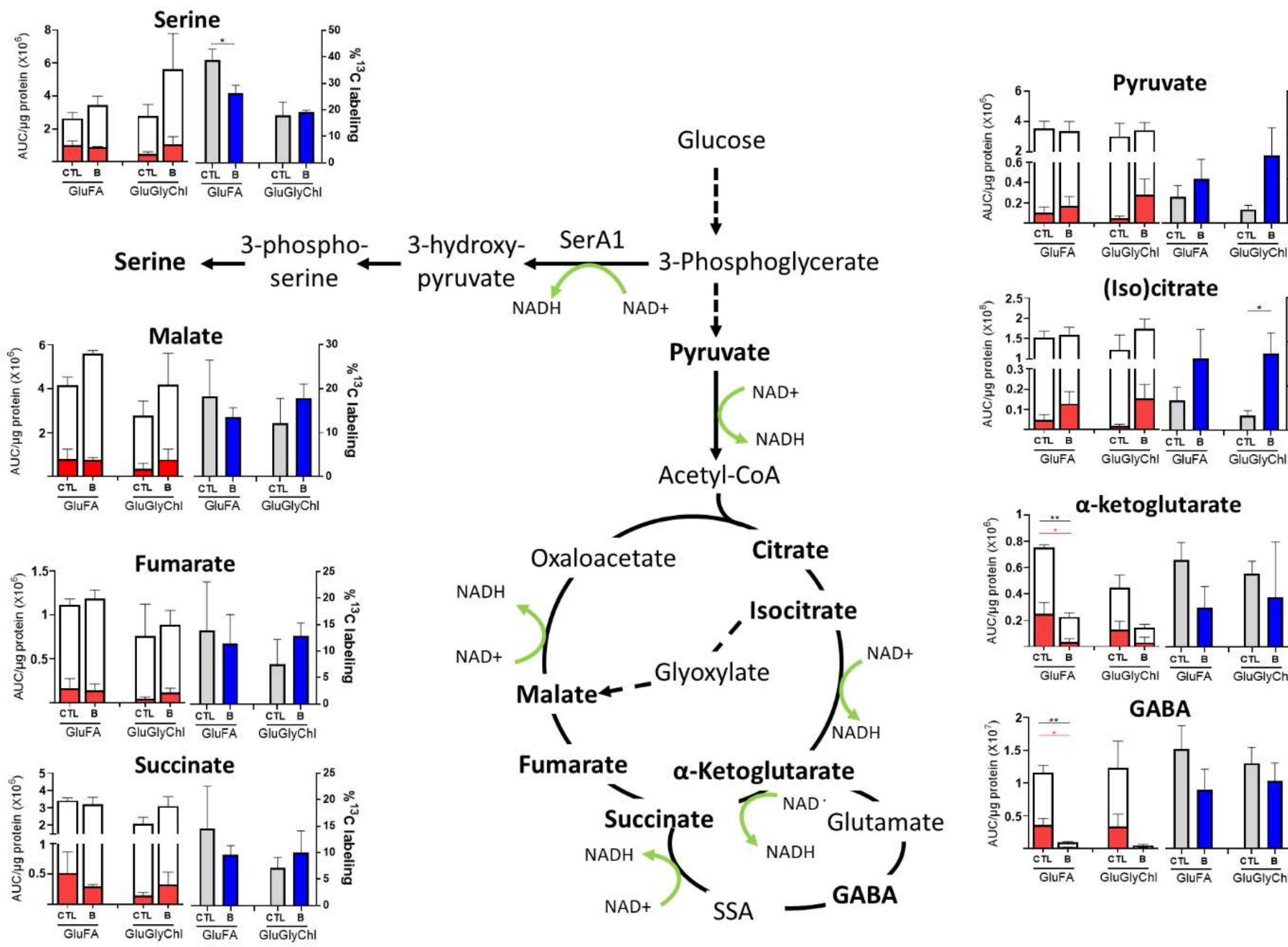

Figure S8. **Impact of bedaquiline on the metabolic flux of central metabolism in *Mtb***. H37Rv *Mtb* was incubated with DMSO or bedaquiline (2 µg/mL) for 24 h in $MMAT_{GluFA}$ or $MMAT_{GluGlyChl}$ (using $^{13}C_6$-Glucose) after which metabolomics experiments were performed, and the metabolites of the TCA cycle extracted: pyruvate, (iso)citrate, succinate, malate, fumarate and GABA (from GABA shunt) as well as serine. For each metabolite, bar charts on the left show (left y axis) the total amount of $^{13}C_6$-labelled metabolites (red bar) and unlabeled metabolites (white bar) measured. The right hand bar charts (white and blue), show the percentage of $^{13}C_6$-labelled metabolites (gray columns are for DMSO-treated sample, blue columns are for bedaquiline-treated samples). Data are the mean and standard deviation of three biological replicates. (CTL): DMSO-treated samples; (B): Bedaquiline-treated (2 µg/mL) samples. Statistical analysis was performed using the t tests with Welch's correction. * p<0.05; ** p<0.01; *** p<0.001.

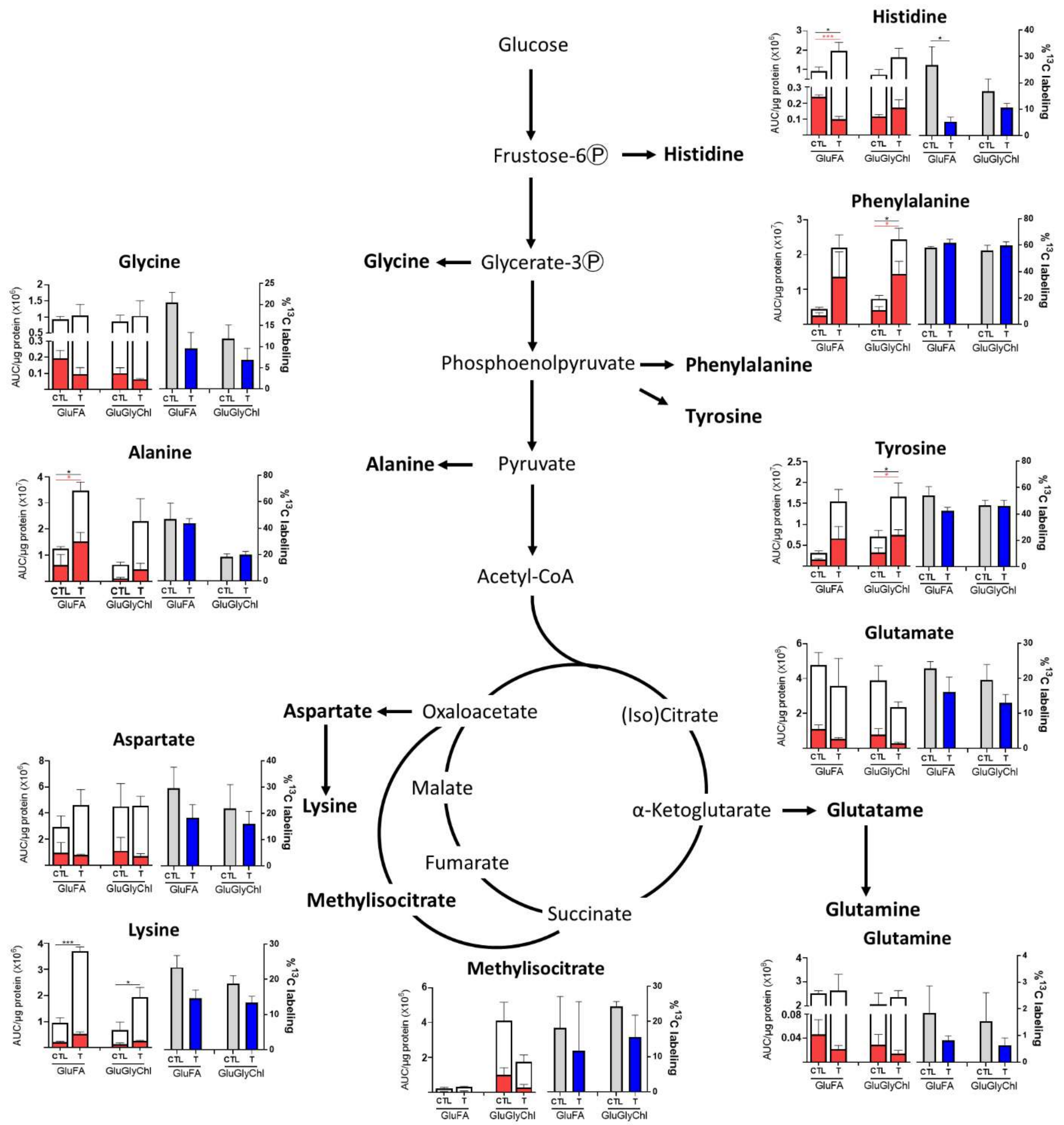

Figure S9. **Impact of TriSLa on the metabolic flux for amino acid biosynthesis and the methylisocitrate cycle of *Mtb***. H37Rv *Mtb* was incubated with DMSO or TriSLa BDM91465 (0.4 µM) for 24 h in $MMAT_{GluFA}$ or $MMAT_{GluGlyChl}$ (using $^{13}C_6$-Glucose) after which metabolomics experiments were performed, and the metabolites of the methylcitrate cycle (methylisocitrate), and amino acids: Glycine, Serine, Histidine, Phenylalanine, Tyrosine, Glutamine, Glutamate, Lysine, Aspartate were evaluated. For each metabolite, bar charts on the left show (left y axis) the total amount of $^{13}C_6$-labelled metabolites (red bar) and unlabeled metabolites (white bar) measured. The right hand bar charts (white and blue), show the percentage of $^{13}C_6$-labelled metabolites (gray columns are for DMSO-treated sample, blue columns are for TriSLa-treated samples). Data are the mean and standard deviation of three biological replicates. (CTL): DMSO-treated samples; (T): TriSLa-treated samples. Statistical analysis was performed using the t tests with Welch's correction. * $p<0.05$; ** $p<0.01$; *** $p<0.001$.

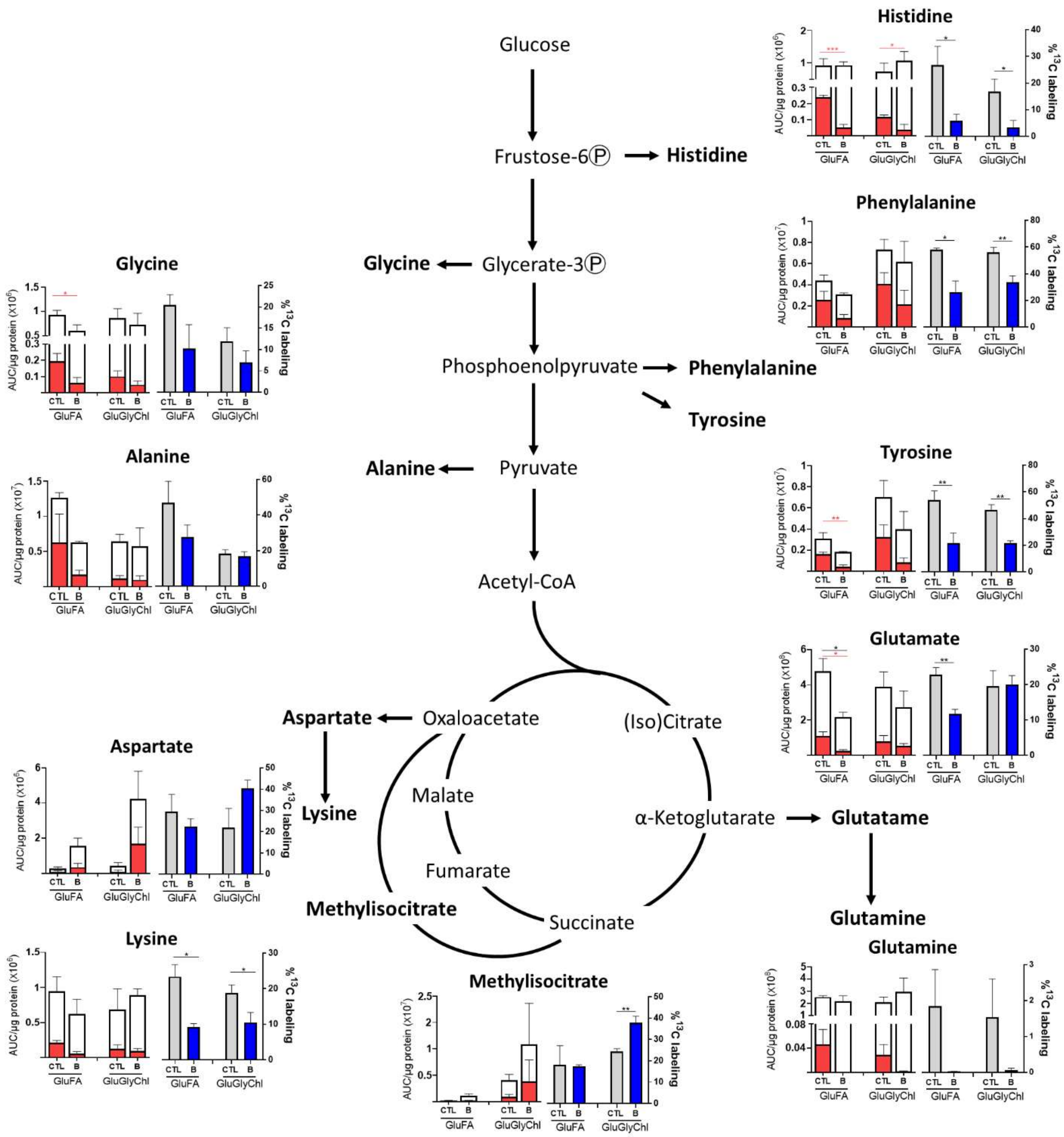

Figure S10. **Impact of Bedaquiline on the TCA cycle, the GABA shunt and the methylisocitrate pathway of *Mtb***. H37Rv *Mtb* was incubated with DMSO or bedaquiline (2 µg/mL) for 24 h in $MMAT_{GluFA}$ or $MMAT_{GluGlyChl}$ (using $^{13}C_6$-Glucose) after which metabolomics experiments were performed, and the metabolites of the methylcitrate cycle (methylisocitrate), and amino acids: Glycine, Serine, Histidine, Phenylalanine, Tyrosine, Glutamine, Glutamate, Lysine, Aspartate were evaluated. For each metabolite, bar charts on the left show (left y axis) the total amount of $^{13}C_6$-labelled metabolites (red bar) and unlabeled metabolites (white bar) measured. The right hand bar charts (white and blue), show the percentage of $^{13}C_6$-labelled metabolites (gray columns are for DMSO-treated sample, blue columns are for bedaquiline-treated samples). Data are the mean and standard deviation of three biological replicates. (CTL): DMSO-treated samples; (B): bedaquiline-treated samples. Statistical analysis was performed using the t tests with Welch's correction. * p<0.05; ** p<0.01; *** p<0.001.

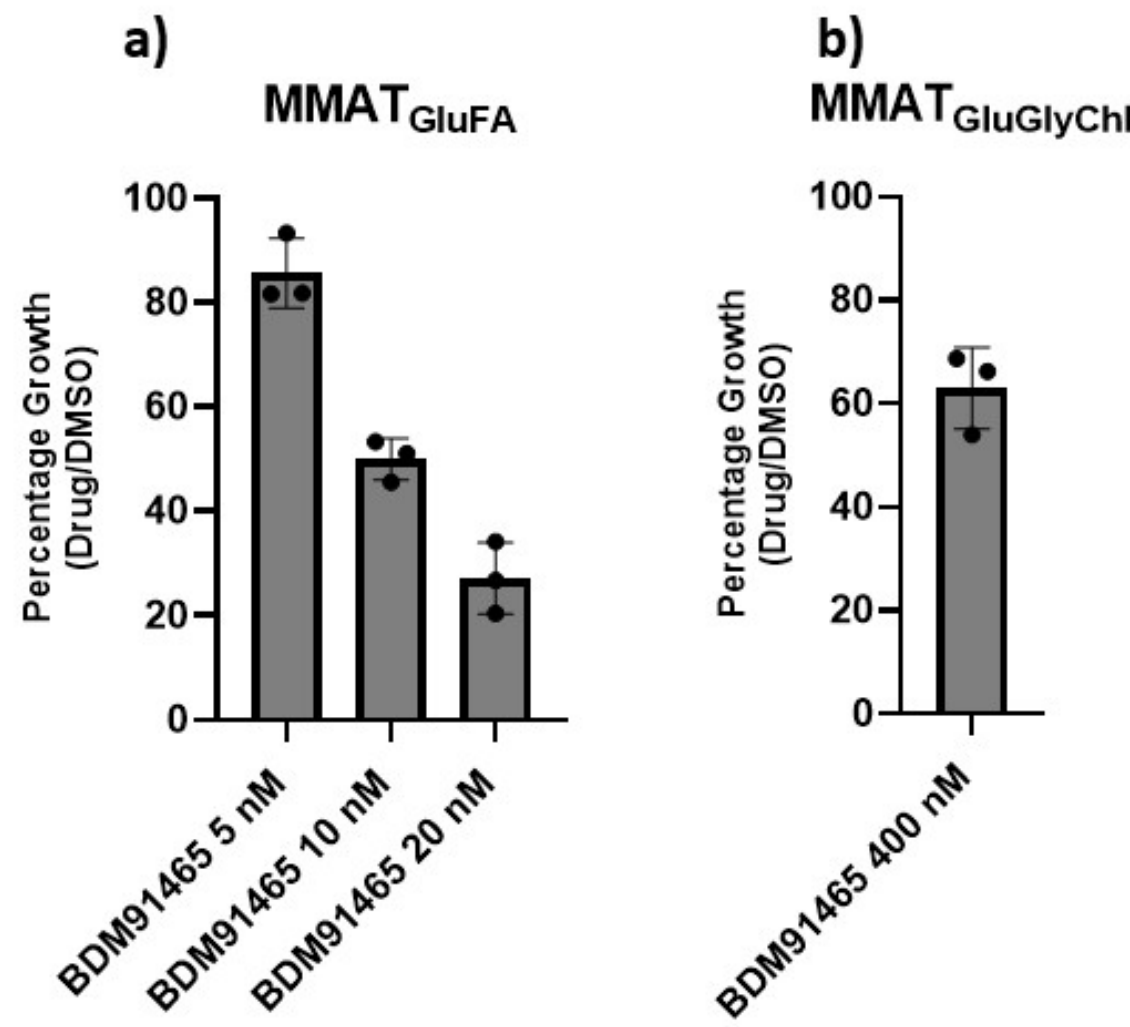

Figure S11. **Growth suppression of the *Mtb* CRISPRi RLC12 library treated with BDM91465 as used for the CRISPRi chemical genomics experiment.** Data shows the final OD_{600} of *Mtb* RLC12 CRISPRi library grown in (**a**) $MMAT_{GluFA}$ and (**b**) $MMAT_{GluGlyChl}$ in the presence of the selected concetrations of BDM91465. Data are represented as an OD_{600} relative to the bacteria treated with DMSO. Data are the mean and SD from three independent experiments used for the actual CRISPRi chemical genomics analysis

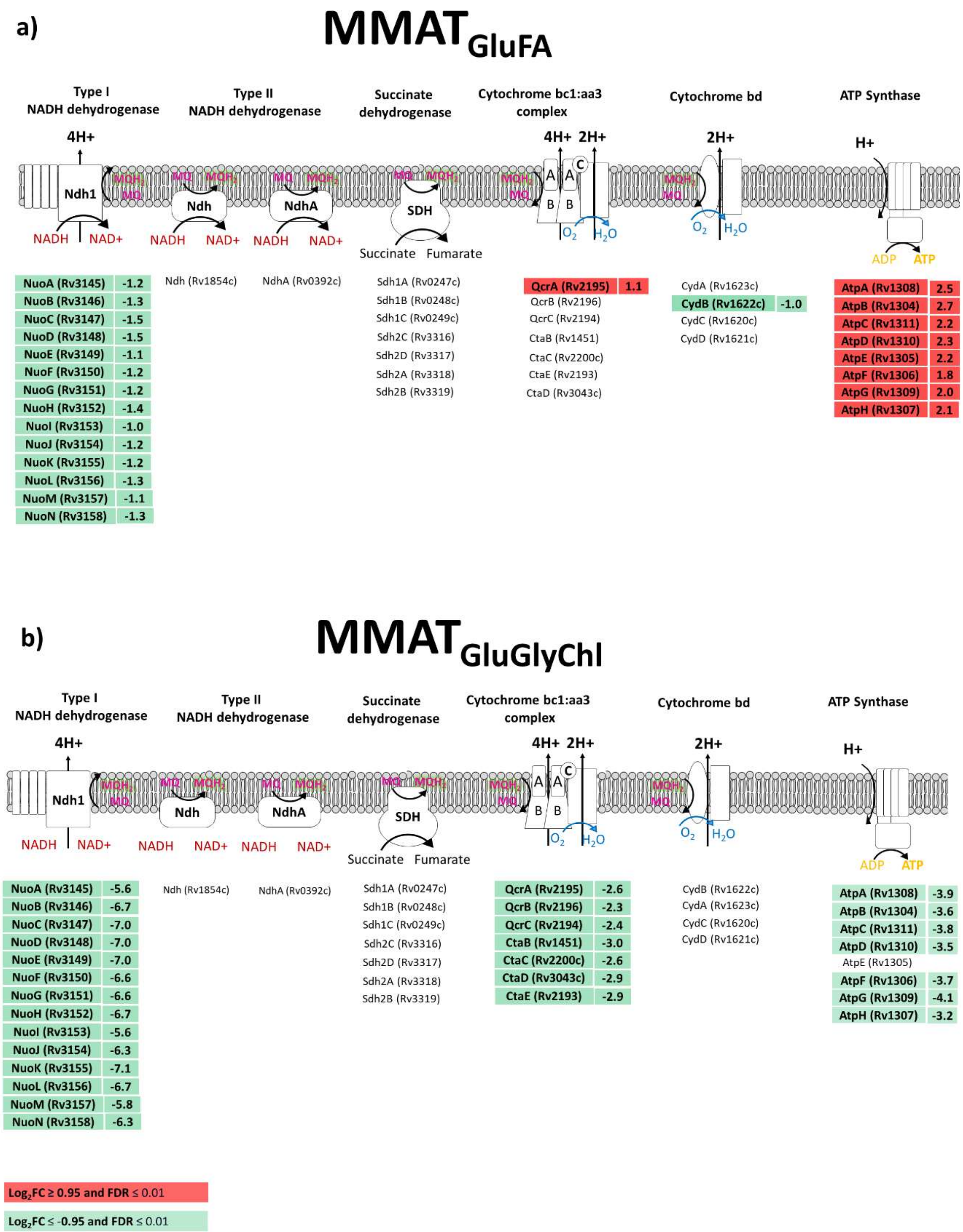

Figure S12. **CRISPRi chemical genomics data analysis - Vulnerability of genes associated with the *Mtb* electron transport chain towards treatment with BDM91465 in both** (**a**) **$MMAT_{GluFA}$ and** (**b**) **$MMAT_{GluGlyChl}$.** Depleted genes (log2FC ≤ -0.95 and FDR ≤ 0.01) with their depletion level are reflected in green and enriched genes (log2FC ≥ 0.95 and FDR ≤ 0.01) with their enrichment level are reflected in red.

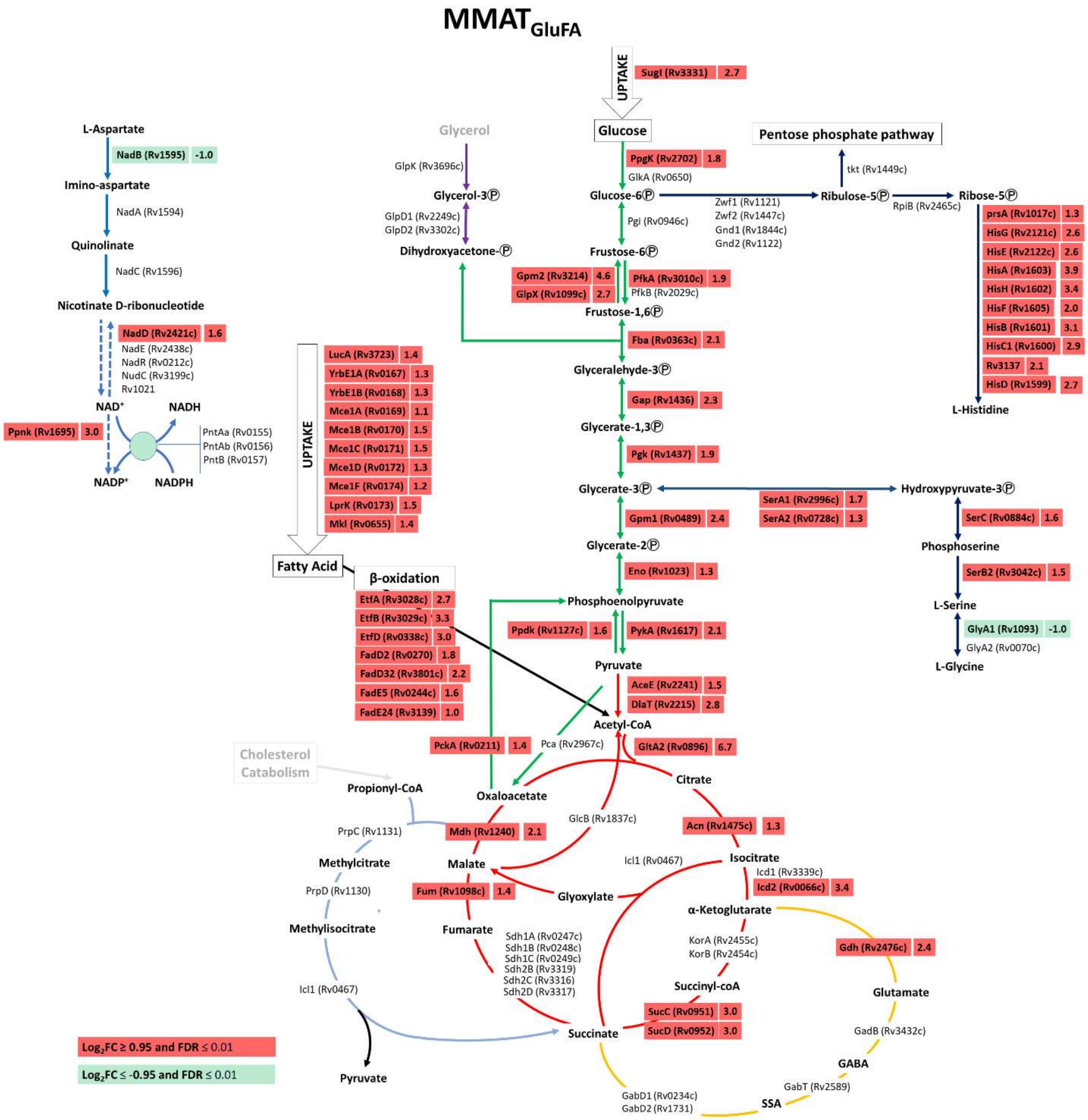

Figure S13. **CRISPRi chemical genomics data analysis - Vulnerability of *Mtb* genes associated with central metabolism towards treatment with BDM91465 in MMAT$_{GluFA}$ media.** Depleted genes (log2FC ≤ -0.95 and FDR ≤ 0.01) with their depletion level are reflected in green and enriched genes (log2FC ≥ 0.95 and FDR ≤ 0.01) with their enrichment level are reflected in red.

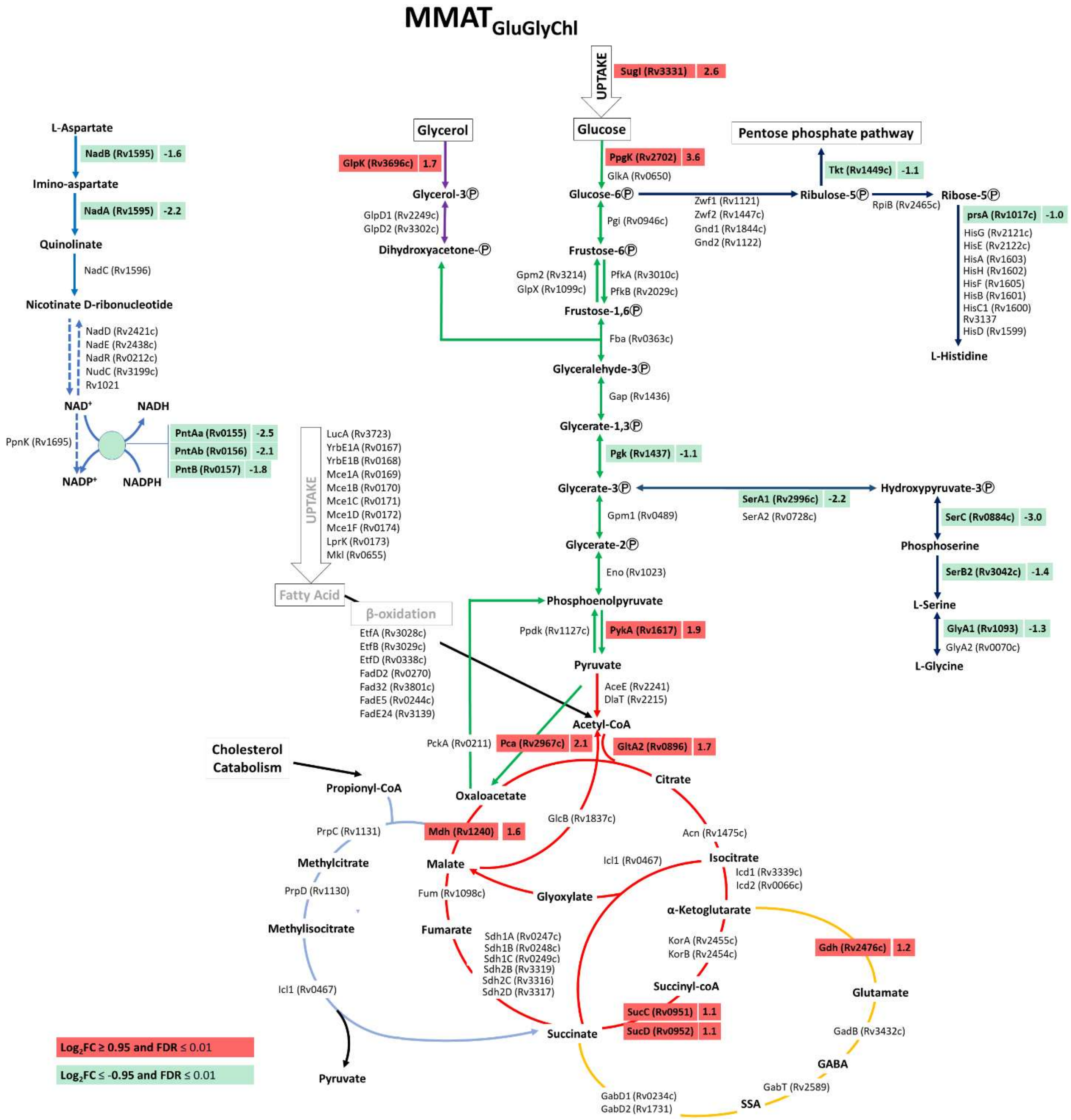

Figure S14. **CRISPRi chemical genomics data analysis - Vulnerability of *Mtb* genes associated with central metabolism towards treatment with BDM91465 in MMAT$_{GluGlyChl}$ media.** Depleted genes (log2FC ≤ -0.95 and FDR ≤ 0.01) with their depletion level are reflected in green and enriched genes (log2FC ≥ 0.95 and FDR ≤ 0.01) with their enrichment level are reflected in red.

a)

BDM88689 (µM) \ ROT (µg/ml)	200	100	50	25	13	6.3	3.1	1.6	0
1.6	1	1	2	1	0	0	1	2	3
0.8	1	1	2	1	1	1	2	3	7
0.4	3	3	4	2	2	3	4	11	13
0.2	11	11	12	13	11	40	61	78	100
0.1	36	42	39	44	66	85	87	99	103
0.05	37	49	32	55	78	88	92	100	116
0	38	41	55	57	77	92	97	102	104

b)

BDM88689 (µM) \ ROT (µg/ml)	200	100	50	25	13	6.3	3.1	1.6	0
1.6	0	0	0	0	1	3	10	22	59
0.8	0	0	0	1	1	5	17	32	78
0.4	1	0	1	1	3	20	39	55	84
0.2	17	19	15	20	41	52	71	82	99
0.1	26	31	28	38	55	68	87	95	101
0.05	28	37	36	46	59	75	91	100	108
0	37	39	39	54	70	87	96	100	104

Figure S15: Synthetic lethality of *Mtb* Ndh-2 inhibition by TriSLa BDM88689 and *Mtb* Ndh-1 inhibition by Rotenone. Checkerboard assay results for BDM88689 in combination with rotenone (ROT) on H37Rv-GFP growing in $MMAT_{GluFA}$ (**a**) and $MMAT_{GluGlyChl}$ (**b**). Bacterial growth was depicted as percentage of GFP fluorescence (Excitation 485 nm, Emission 510 nm) compared to no drug control. Percentage GFP is colored arbitrarily to facilitate analysis. Data represent the mean of at least 2 biological replicates.

a)

BDM91465 (nM) \ ROT (µg/ml)	200	100	50	25	13	6	3	2	0
160	0	0	1	1	3	5	13	21	55
80	0	-1	3	6	6	11	14	30	67
40	-1	-1	3	4	13	29	36	55	84
20	2	-1	4	21	30	47	58	75	96
10	25	21	25	35	52	63	73	85	98
5	31	34	39	51	67	78	89	98	104
0	45	37	50	62	83	96	109	107	112

b)

BDM91465 (nM) \ ROT (µg/ml)	200	100	50	25	13	6	3	2	0
160	23	24	27	41	66	92	100	98	103
80	30	31	40	61	86	99	105	109	107
40	25	36	52	68	85	99	105	109	110
20	25	33	56	80	92	98	97	106	108
10	25	36	57	81	91	97	99	107	111
5	35	45	63	82	96	104	104	108	112
0	27	47	59	83	93	100	107	110	116

Figure S16. TriSLa-resistant *Mtb* is also resistant against the synthetic lethality of Ndh-2 inhibition by TriSLa BDM91465 and Ndh-1 inhibition by rotenone. Checkerboard assay results for BDM91465 in combination with rotenone (ROT) against (**a**) H37Rv-GFP and (**b**) H37Rv TriSLa resistant clone RC1.1-GFP *Mtb* (harboring Y403H in Ndh). Bacterial growth was depicted as percentage of GFP fluorescence (Excitation 485 nm, Emission 510 nm) compared to no drug control (values were background corrected). Percentage GFP is colored arbitrarily to facilitate analysis. Data represent the mean of at least 2 biological replicates

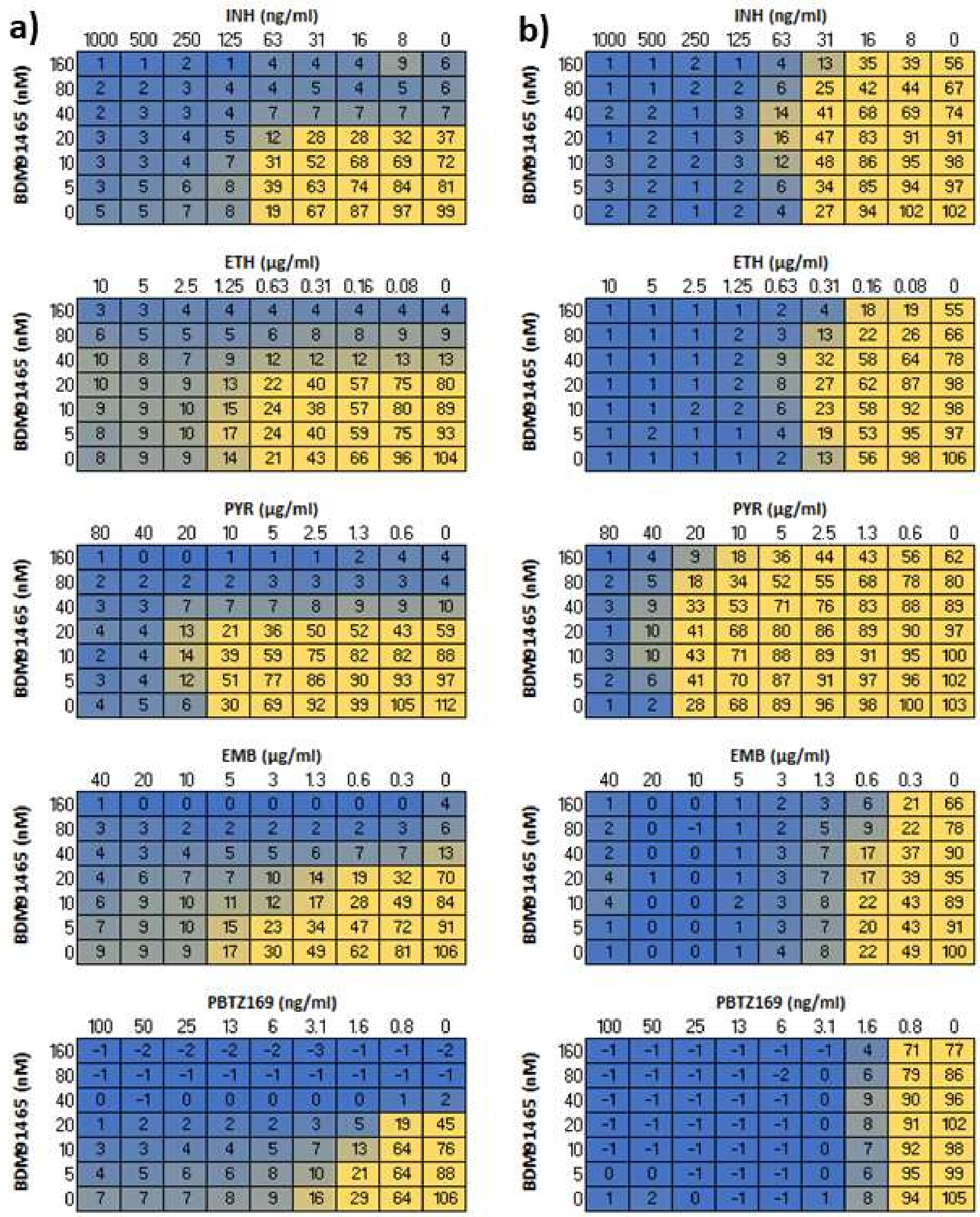

Figure S17: Evaluation of growth inhibition activity of TriSLa BDM91465 in combination with bacterial cell wall inhibitors. Checkerboard assay results of TriSLa BDM91465 in combination with antibiotics targeting the bacterial cell wall including isoniazid (INH), ethionamide (ETH), pyridomycin (PYR), ethambutol (EMB), macozinone (PBTZ169) against H37Rv-GFP, in both (**a**) $MMAT_{GluFA}$ and (**b**) $MMAT_{GluGlyChl}$. Bacterial growth was depicted as percentage of GFP fluorescence (Excitation 485 nm, Emission 510 nm) compared to no drug control (Values were background corrected). Percentage GFP is colored arbitrarily to facilitate analysis. Data represent the means of at least 2 biological replicates

a)

INH (ng/ml)

BDM88689 (µM)	1000	500	250	125	63	31	16	8	0
1.6	2	1	2	1	2	2	2	2	2
0.8	3	3	3	3	4	4	5	7	9
0.4	4	4	4	5	4	5	10	8	9
0.2	5	5	5	8	17	39	45	52	62
0.1	6	5	6	11	39	64	63	78	84
0.05	6	6	6	10	34	55	75	75	82
0	6	6	6	7	18	54	71	92	91

ETH (µg/ml)

BDM88689 (µM)	10	5	2.5	1.25	0.63	0.31	0.16	0.08	0
1.6	3	3	3	3	4	3	4	3	4
0.8	5	4	5	6	7	8	8	7	8
0.4	12	10	11	12	13	12	12	12	11
0.2	11	11	12	17	27	44	68	82	100
0.1	12	11	11	17	24	43	62	83	101
0.05	11	11	10	16	22	38	64	81	94
0	11	8	10	14	23	38	66	81	96

PYR (µg/ml)

BDM88689 (µM)	80	40	20	10	5	2.5	1.3	0.6	0
1.6	2	0	0	0	0	-1	-1	0	4
0.8	3	3	3	3	3	2	2	4	7
0.4	4	4	7	8	8	8	8	10	11
0.2	6	5	9	27	43	53	66	72	76
0.1	4	6	10	35	65	82	91	91	95
0.05	5	5	9	35	69	84	96	93	100
0	5	4	6	26	61	85	96	96	103

EMB (µg/ml)

BDM88689 (µM)	40	20	10	5	3	1.3	0.6	0.3	0
1.6	1	0	0	0	-1	-1	-1	0	2
0.8	4	4	4	4	4	3	4	3	6
0.4	7	8	10	11	11	9	9	10	11
0.2	7	10	13	18	26	40	50	63	79
0.1	8	10	12	18	30	47	61	80	96
0.05	9	11	10	16	30	47	61	78	102
0	9	8	10	16	27	47	60	84	103

PBTZ169 (ng/ml)

BDM88689 (µM)	100	50	25	13	6	3.1	1.6	0.8	0
1.6	2	0	1	0	0	-1	-1	-1	0
0.8	3	1	1	0	1	0	0	0	3
0.4	2	2	3	3	3	3	3	5	8
0.2	4	4	4	5	5	9	17	49	74
0.1	5	5	4	5	7	11	27	71	94
0.05	6	6	5	6	6	13	27	71	98
0	6	6	5	6	6	10	27	73	103

b)

INH (ng/ml)

BDM88689 (µM)	1000	500	250	125	63	31	16	8	0
1.6	3	1	1	2	4	16	50	62	63
0.8	0	2	2	5	10	24	56	61	79
0.4	0	2	3	6	13	43	77	85	89
0.2	0	2	3	7	11	44	88	95	98
0.1	1	2	2	4	11	43	90	96	100
0.05	1	2	2	3	8	39	93	96	105
0	2	2	2	2	5	33	87	96	98

ETH (µg/ml)

BDM88689 (µM)	10	5	2.5	1.25	0.63	0.31	0.16	0.08	0
1.6	1	1	2	2	4	8	20	37	53
0.8	1	2	2	2	4	14	30	42	68
0.4	2	2	2	3	12	33	57	68	79
0.2	2	2	2	4	11	31	63	80	88
0.1	2	2	2	3	6	26	59	88	98
0.05	2	2	1	2	4	19	56	94	102
0	2	1	1	1	3	12	48	93	100

PYR (µg/ml)

BDM88689 (µM)	80	40	20	10	5	2.5	1.3	0.6	0
1.6	0	3	13	32	63	71	75	72	73
0.8	0	4	17	42	64	78	75	83	90
0.4	3	5	30	68	78	86	86	92	98
0.2	1	6	38	68	80	87	90	94	98
0.1	1	3	40	68	81	87	94	93	99
0.05	0	4	39	68	82	89	88	95	98
0	0	1	31	71	83	87	90	97	98

EMB (µg/ml)

BDM88689 (µM)	40	20	10	5	3	1.3	0.6	0.3	0
1.6	1	0	0	2	4	7	14	34	78
0.8	3	1	1	2	5	9	16	37	84
0.4	4	3	2	3	5	9	17	44	88
0.2	4	2	1	2	5	10	23	46	91
0.1	2	1	1	2	4	8	21	39	92
0.05	1	1	0	2	4	8	22	44	90
0	1	1	0	1	4	9	23	46	94

PBTZ169 (ng/ml)

BDM88689 (µM)	100	50	25	13	6	3.1	1.6	0.8	0
1.6	-1	-1	0	-1	-1	0	6	86	96
0.8	-1	0	0	0	0	1	8	89	97
0.4	-1	0	0	0	0	1	9	96	103
0.2	-1	-1	0	0	0	1	10	95	104
0.1	0	0	-1	-1	-1	1	9	92	103
0.05	-1	-1	-1	0	0	1	10	94	103
0	-1	-1	-1	-1	-1	0	8	93	101

Figure S18: Evaluation of growth inhibition anti-*Mtb* activity of TriSLa BDM88689 in combination with bacterial cell wall inhibitors. Checkerboard assay results for TriSLa BDM88689 in combination with antibiotics targeting the bacterial cell wall including isoniazid (INH), ethionamide (ETH), pyridomycin (PYR), ethambutol (EMB), macozinone (PBTZ169) against H37Rv-GFP, in both (**a**) $MMAT_{GluFA}$ and (**b**) $MMAT_{GluGlyChl}$. Bacterial growth was depicted as percentage of GFP fluorescence (Excitation 485 nm, Emission 510 nm) compared to no drug control (Values were background corrected). Percentage GFP is colored arbitrarily to facilitate analysis. Data represent the means of at least 2 biological replicates

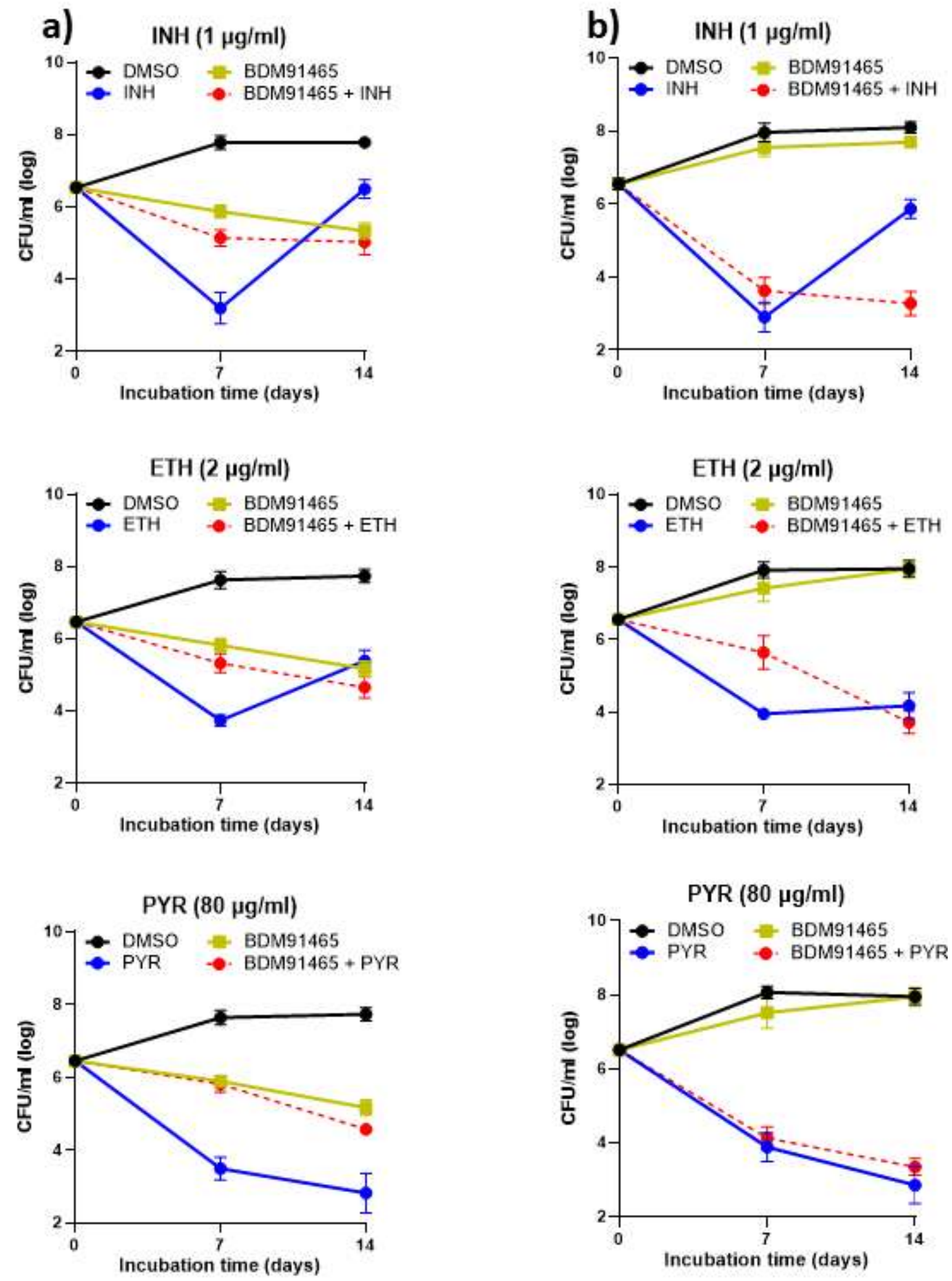

Figure S19. Evaluation of bactericidal anti-*Mtb* activity of TriSLa BDM91465 in combination with bacterial cell wall inhibitors in 2-week period. Time-kill assays of combinations of "super-MIC" concentrations of TriSLa BDM91465 (0.4 uM) in combination with isoniazid (INH), ethionamide (ETH) and pyridomycin (PYR) against H37Rv, in (**a**) $MMAT_{GluFA}$ or (**b**) $MMAT_{GluGlyChl}$ against H37Rv as measured by CFU over a 2-week period. Data show that TriSLa combinations with isoniazid and ethionamide prevent the emergence of likely resistance to the antibiotics alone. Bacterial survival was depicted as CFU/ml and values are average with SD of at least 3 biological replicates.

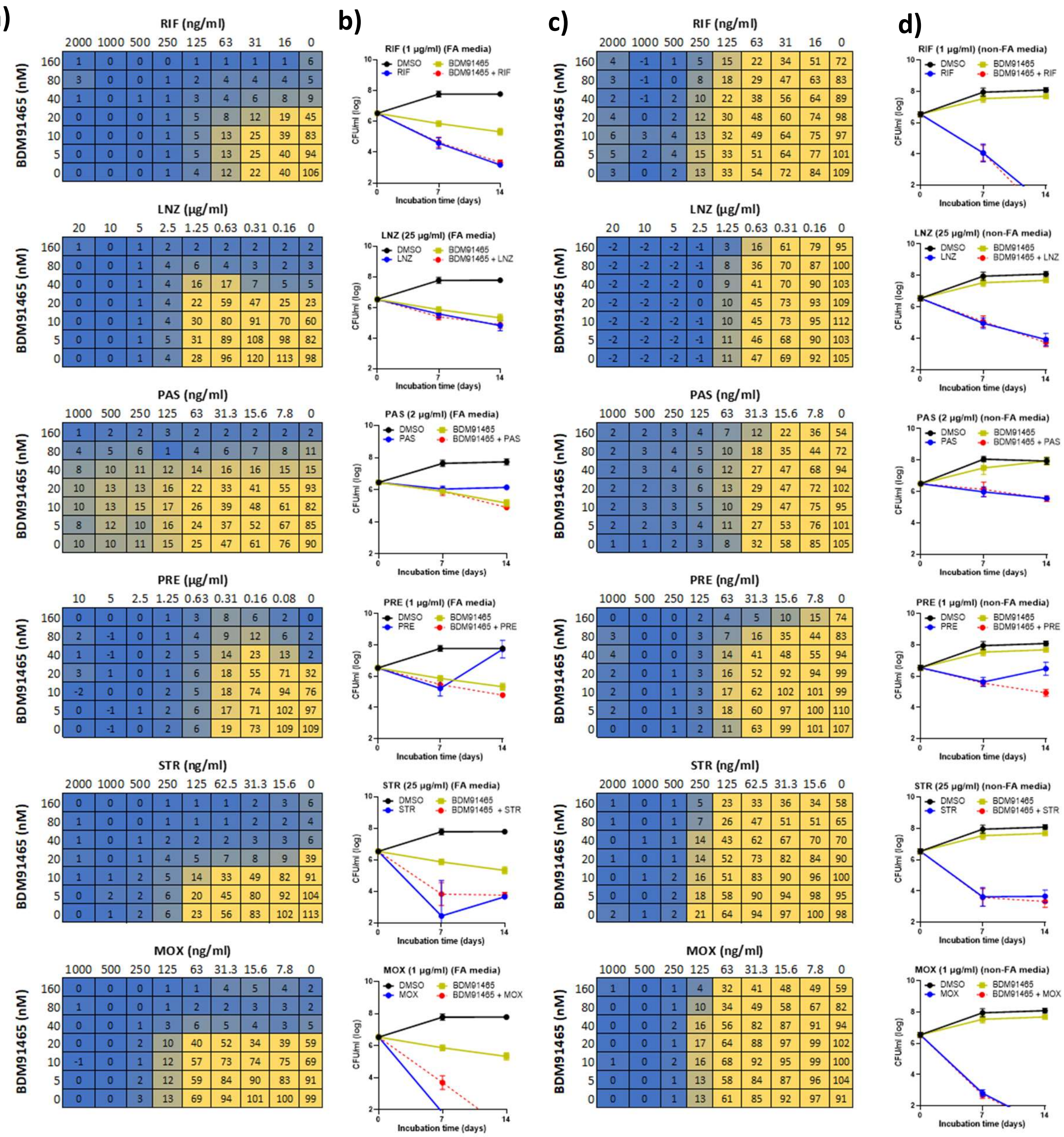

Figure S20. Evaluation of growth inhibition and bactericidal anti-*Mtb* activity of TriSLa BDM91465 in combination with different antituberculosis drugs. (**a**, **c**) Checkerboard assay results (indicating percentage GFP signal) and time-kill assay results for TriSLa BDM91465 in combination with rifampicin (RIF), linezolid (LNZ), *para*-Aminosalicylic Acid (PAS), pretomanid (PRE), streptomycin (STR) and moxifloxacin (MOX) against H37Rv-GFP. Combination studies were performed in (**a**, **b**) $MMAT_{GluFA}$ or (**c**, **d**) $MMAT_{GluGlyChl}$ media. Checkerboard data shows bacterial growth as percentage of GFP fluorescence (Excitation 485 nm, Emission 510 nm) compared to no drug control (Values were background corrected). Percentage GFP is colored arbitrarily to facilitate analysis. Data for checkerboard assay represent the means of at least 2 biological replicates. (**b**, **d**) Time-kill kinetics were performed with TriSLa BDM91465 (0.4 µM) in combination with anti-tuberculosis drugs including RIF (1 µg/ml), LNZ (25 µg/ml), PAS (1 µg/ml), PRE (1 µg/ml), STR (25 µg/ml), MOX (1 µg/ml) for a 1-week or 2-week period. Bacterial survival was depicted as CFU/ml and values are average with SD of at least 3 biological replicates.

a)

RIF (ng/ml)

BDM88689 (µM)	2000	1000	500	250	125	63	31	16	0
1.6	0	1	0	0	1	1	1	1	1
0.8	2	1	0	1	2	3	4	7	8
0.4	3	2	0	2	3	4	9	10	12
0.2	2	1	1	1	4	9	15	27	61
0.1	4	0	2	3	5	11	23	32	90
0.05	3	1	0	2	4	11	22	35	94
0	3	1	0	2	4	11	21	38	98

LNZ (µg/ml)

BDM88689 (µM)	20	10	5	2.5	1.25	0.63	0.31	0.16	0
1.6	0	0	0	3	2	2	2	2	3
0.8	0	0	1	4	6	3	3	2	2
0.4	0	0	1	5	23	18	6	5	4
0.2	0	0	0	5	32	89	90	60	52
0.1	0	0	1	5	28	98	119	108	91
0.05	0	0	0	5	28	97	123	118	101
0	1	0	1	4	26	102	125	114	106

PAS (ng/ml)

BDM88689 (µM)	1000	500	250	125	63	31.3	15.6	7.8	0
1.6	3	2	2	2	2	3	2	2	3
0.8	5	5	4	5	6	7	7	7	9
0.4	11	13	15	17	20	22	21	21	21
0.2	13	14	21	26	38	50	62	77	105
0.1	11	14	17	26	34	48	67	81	108
0.05	11	13	17	21	31	46	67	86	104
0	8	11	14	21	30	42	63	83	104

PRE (µg/ml)

BDM88689 (µM)	10	5	2.5	1.25	0.63	0.31	0.16	0.08	0
1.6	-2	-1	-1	1	4	7	7	3	1
0.8	-2	-1	0	1	5	10	13	7	1
0.4	-1	-1	0	1	4	13	30	26	3
0.2	0	-1	-1	1	5	18	60	79	39
0.1	-1	-1	0	1	6	17	64	92	86
0.05	0	-1	0	2	5	19	63	97	98
0	3	-1	0	2	7	20	66	100	101

STR (ng/ml)

BDM88689 (µM)	2000	1000	500	250	125	62.5	31.3	15.6	0
1.6	2	3	1	1	2	2	2	2	9
0.8	2	0	2	1	2	2	2	3	3
0.4	3	0	1	2	3	4	5	3	8
0.2	3	4	4	4	13	31	51	52	69
0.1	4	1	3	6	23	52	72	91	108
0.05	0	0	2	6	22	57	76	93	106
0	0	0	2	6	23	57	83	97	107

MOX (ng/ml)

BDM88689 (µM)	1000	500	250	125	63	31.3	15.6	7.8	0
1.6	0	0	0	0	1	1	1	1	1
0.8	1	0	1	1	1	3	2	3	2
0.4	1	0	1	3	6	5	5	5	6
0.2	3	0	2	10	43	61	61	60	62
0.1	1	0	1	11	57	87	90	93	93
0.05	1	0	1	11	55	89	98	96	96
0	1	1	2	12	63	93	99	97	100

b)

RIF (ng/ml)

BDM88689 (µM)	2000	1000	500	250	125	63	31	16	0
1.6	1	0	0	5	13	19	34	46	84
0.8	1	1	2	8	17	30	46	55	83
0.4	3	1	2	12	27	43	54	67	90
0.2	1	1	1	13	30	48	62	74	96
0.1	1	1	2	15	34	50	64	79	100
0.05	2	-1	2	13	34	46	66	77	98
0	0	0	2	12	32	51	61	76	97

LNZ (µg/ml)

BDM88689 (µM)	20	10	5	2.5	1.25	0.63	0.31	0.16	0
1.6	-2	-2	-2	0	4	31	70	84	98
0.8	-2	-2	-2	0	11	46	73	98	107
0.4	-2	-2	-2	0	14	55	81	97	108
0.2	-2	-2	-2	0	15	55	87	93	114
0.1	-2	-2	-2	0	14	55	81	99	109
0.05	-2	-2	-2	0	13	52	80	97	106
0	-2	-2	-2	0	12	56	81	99	110

PAS (ng/ml)

BDM88689 (µM)	1000	500	250	125	63	31.3	15.6	7.8	0
1.6	3	3	4	5	8	16	25	38	55
0.8	3	4	5	7	10	22	37	54	59
0.4	3	4	6	8	15	29	45	63	87
0.2	3	3	4	8	15	31	49	68	88
0.1	2	3	4	7	13	28	49	68	87
0.05	2	3	3	5	11	29	49	71	95
0	2	2	3	5	8	25	52	80	100

PRE (ng/ml)

BDM88689 (µM)	1000	500	250	125	63	31.3	15.6	7.8	0
1.6	0	0	0	1	2	4	9	17	74
0.8	0	1	0	2	5	11	28	41	91
0.4	5	1	0	1	10	36	83	66	98
0.2	1	0	0	2	13	47	106	78	107
0.1	2	0	0	2	11	51	99	100	101
0.05	3	0	0	2	11	57	103	99	102
0	0	0	0	2	10	49	105	105	102

STR (ng/ml)

BDM88689 (µM)	2000	1000	500	250	125	62.5	31.3	15.6	0
1.6	0	0	0	6	28	30	30	34	62
0.8	1	0	1	10	36	48	48	45	64
0.4	0	0	1	12	49	72	84	74	77
0.2	0	1	2	15	52	82	91	90	99
0.1	0	0	1	16	47	80	93	95	98
0.05	0	0	2	19	57	85	94	97	100
0	2	0	2	17	51	83	94	97	103

MOX (ng/ml)

BDM88689 (µM)	1000	500	250	125	63	31.3	15.6	7.8	0
1.6	1	0	1	16	36	54	65	76	79
0.8	1	1	2	11	42	69	76	82	86
0.4	1	0	2	18	64	87	92	98	98
0.2	2	0	2	17	64	91	95	102	102
0.1	2	0	2	16	65	90	93	99	106
0.05	2	0	2	16	67	91	97	104	108
0	1	0	2	14	65	94	100	101	104

Figure S21. Evaluation of growth inhibition anti-*Mtb* activity of TriSLa BDM88689 in combination with different anti-*Mtb* drugs. Checkerboard assay results (indicating percentage bacterial GFP signal) for TriSLa BDM88689 in combination with rifampicin (RIF), linezolid (LNZ), *para*-Aminosalicylic Acid (PAS), pretomanid (PRE), streptomycin (STR) and moxifloxacin (MOX) against H37Rv-GFP. Combination studies were performed in (**a**) $MMAT_{GluFA}$ or (**b**) $MMAT_{GluGlyChl}$ media. Bacterial growth was depicted as percentage of GFP fluorescence (Excitation 485 nm, Emission 510 nm) compared to no drug control (Values were background corrected). Percentage GFP is colored arbitrarily to facilitate analysis. Data represent the means of at least 2 biological replicates.

a)

BDQ (ng/ml)

BDM88689 (µM)	4000	2000	1000	500	250	125	63	31	0
1.6	0	1	1	1	1	1	1	1	2
0.8	1	2	3	3	2	2	2	2	3
0.4	0	2	4	5	6	4	4	4	4
0.2	1	2	4	8	11	11	11	14	41
0.1	1	2	4	7	14	18	26	39	86
0.05	1	2	4	8	15	22	32	42	95
0	0	2	4	8	17	24	30	43	99

Q203 (ng/ml)

BDM88689 (µM)	500	250	125	63	31	15.6	7.8	3.9	0
1.6	5	4	4	3	3	3	2	2	3
0.8	8	9	8	8	7	7	6	5	4
0.4	26	29	31	31	36	34	28	22	6
0.2	29	32	34	44	52	56	61	66	44
0.1	30	34	38	43	53	62	70	78	90
0.05	30	34	38	48	54	65	66	75	92
0	31	34	38	45	56	65	68	74	103

CFZ (µg/ml)

BDM88689 (µM)	10	5	2.5	1.25	0.63	0.31	0.16	0.08	0
1.6	1	1	0	1	1	1	1	1	1
0.8	1	1	2	1	2	2	2	2	2
0.4	1	3	4	5	7	7	6	5	5
0.2	3	10	12	28	43	49	60	67	70
0.1	2	3	16	33	53	64	80	87	97
0.05	4	4	22	31	59	70	85	92	107
0	2	4	17	51	67	82	89	94	105

b)

BDQ (ng/ml)

BDM88689 (µM)	4000	2000	1000	500	250	125	63	31	0
1.6	0	-1	-1	-1	-1	0	4	31	85
0.8	0	-1	-1	0	2	6	18	44	86
0.4	0	-1	0	1	4	16	34	64	92
0.2	0	-1	0	1	4	16	40	68	98
0.1	4	-1	0	1	4	15	40	69	102
0.05	2	-1	0	1	4	16	36	73	101
0	1	-1	-1	1	3	14	35	68	103

Q203 (ng/ml)

BDM88689 (µM)	50	25	12.5	6.25	3.13	1.56	0.78	0.39	0
1.6	1	0	1	0	0	1	1	4	65
0.8	3	2	2	2	3	4	7	12	70
0.4	8	6	9	12	21	37	49	59	76
0.2	8	9	12	20	41	66	80	89	95
0.1	8	9	13	22	45	77	94	103	103
0.05	8	10	14	24	54	84	104	110	115
0	7	9	13	24	61	89	105	115	116

CFZ (µg/ml)

BDM88689 (µM)	10	5	2.5	1.25	0.63	0.31	0.16	0.08	0
1.6	0	0	1	1	1	3	5	11	62
0.8	0	0	1	1	3	10	16	27	78
0.4	0	0	2	2	10	20	37	61	82
0.2	0	2	4	16	21	30	62	79	99
0.1	1	0	6	13	25	37	64	85	102
0.05	1	1	10	17	30	44	70	91	101
0	0	2	12	23	34	53	76	94	102

Figure S22: Synergistic activity of TriSLa BDM88689 with bacterial respiration inhibitors against *Mtb*. Checkerboard assays results for the combinations of TriSLa BDM88689 and bedaquiline (BDQ), telacebec (Q203) and clofazimine (CFZ) against H37Rv-GFP in (**a**) $MMAT_{GluFA}$ or (**b**) $MMAT_{GluGlyChl}$. Checkerboard data shows bacterial growth as percentage of GFP fluorescence (Excitation 485 nm, Emission 510 nm) compared to no drug control (Values were background corrected). Percentage GFP is colored arbitrarily to facilitate analysis. Data for checkerboard assay represent the mean of at least 2 biological replicates

a)

BDM91465 (nM) \ BDQ (ng/ml)	4000	2000	1000	500	250	125	63	31	0
160	0	1	2	3	2	2	10	17	55
80	0	0	2	5	7	10	13	25	63
40	0	0	2	4	8	11	32	38	73
20	0	1	2	4	9	16	34	56	82
10	0	0	1	5	8	14	36	63	83
5	0	1	2	5	12	20	41	68	90
0	-1	1	2	6	13	22	42	65	91

b)

BDM91465 (nM) \ BDQ (ng/ml)	4000	2000	1000	500	250	125	63	31	0
160	-1	-1	1	2	5	14	45	72	86
80	1	-1	0	1	6	19	50	78	96
40	-1	-1	0	1	6	23	50	83	100
20	2	-1	0	1	7	21	46	77	105
10	3	0	0	2	8	21	47	73	101
5	1	1	2	4	9	22	49	76	104
0	2	2	1	4	11	24	52	80	105

c)

BDM91465 (nM) \ Q203 (ng/ml)	50	25	12.5	6.25	3.13	1.56	0.78	0.39	0
160	1	1	1	1	2	0	1	4	54
80	6	2	6	2	3	11	9	40	78
40	6	13	9	11	19	26	28	54	90
20	7	14	17	18	31	55	60	86	97
10	8	9	13	18	36	59	87	92	105
5	8	10	19	18	46	77	83	97	103
0	7	10	13	17	54	86	101	104	111

d)

BDM91465 (nM) \ Q203 (ng/ml)	50	25	12.5	6.25	3.13	1.56	0.78	0.39	0
160	7	5	8	8	14	31	84	102	100
80	9	6	10	9	24	38	90	93	103
40	5	11	9	18	28	70	88	113	108
20	5	8	11	18	39	51	97	109	118
10	4	9	10	17	29	61	107	110	107
5	7	8	9	23	31	84	98	82	109
0	10	9	11	28	28	73	69	98	121

e)

BDM91465 (nM) \ CFZ (µg/ml)	10	5	2.5	1.25	0.63	0.31	0.16	0.08	0
160	0	0	1	0	1	1	5	15	65
80	2	0	0	1	1	15	26	42	75
40	0	0	1	2	11	16	33	58	85
20	1	2	2	9	15	22	63	95	92
10	1	1	13	13	20	60	68	89	101
5	0	2	9	18	28	51	77	87	103
0	0	4	20	27	23	59	90	98	102

f)

BDM91465 (nM) \ CFZ (µg/ml)	10	5	2.5	1.25	0.63	0.31	0.16	0.08	0
160	2	2	3	7	25	45	57	88	104
80	3	1	4	25	34	39	71	103	109
40	1	3	15	31	42	53	81	92	108
20	1	2	19	28	44	72	85	101	102
10	1	7	14	27	43	70	99	101	121
5	1	9	14	29	55	84	110	107	113
0	0	4	20	34	64	102	91	123	110

Figure S23: Synergistic activity of TriSLa BDM91465 with bacterial respiration inhibitors MMAT$_{GluGlyChl}$ against *Mtb* were mediated via Ndh-2 inhibition of TriSLa. Checkerboard assays for BDM91465 in combinations with (**a**, **b**) bedaquiline (BDQ), (**c**, **d**) telacebec (Q203) and (**e**, **f**) clofazimine (CFZ) against (**a**, **c**, **e**) WT H37Rv-GFP and (**b**, **d**, **f**) TriSLa-resistant H37Rv clone RC1.1 *Mtb* (Ndh mutation Y403H) expressing GFP Bacterial growth was depicted as percentage of GFP fluorescence (Excitation 485 nm, Emission 510 nm) compared to no drug control (Values were background corrected). Percentage GFP is colored arbitrarily to facilitate analysis. Data represent the mean of at least 2 biological replicates.

a)

AX-35 (µg/ml) vs. BDM91465 (nM)

BDM91465 (nM)	20	10	5	2.5	1.25	0.63	0.31	0.16	0
160	8	3	2	1	1	1	1	2	2
80	8	3	6	6	5	2	2	2	4
40	8	9	13	21	24	15	12	8	4
20	10	11	17	25	50	56	53	49	49
10	9	13	19	30	49	76	89	84	82
5	15	14	23	33	53	77	90	93	90
0	20	17	22	33	59	86	96	101	99

11626252 (µg/ml) vs. BDM91465 (nM)

BDM91465 (nM)	10	5	3	1.25	0.63	0.31	0.16	0.08	0
160	7	2	0	0	0	1	0	0	4
80	7	6	3	5	6	7	5	2	4
40	7	7	7	9	14	19	24	14	5
20	10	8	9	12	15	24	39	62	38
10	10	11	11	14	18	26	42	80	79
5	12	13	15	16	20	27	41	79	92
0	14	16	17	19	23	32	47	79	94

b)

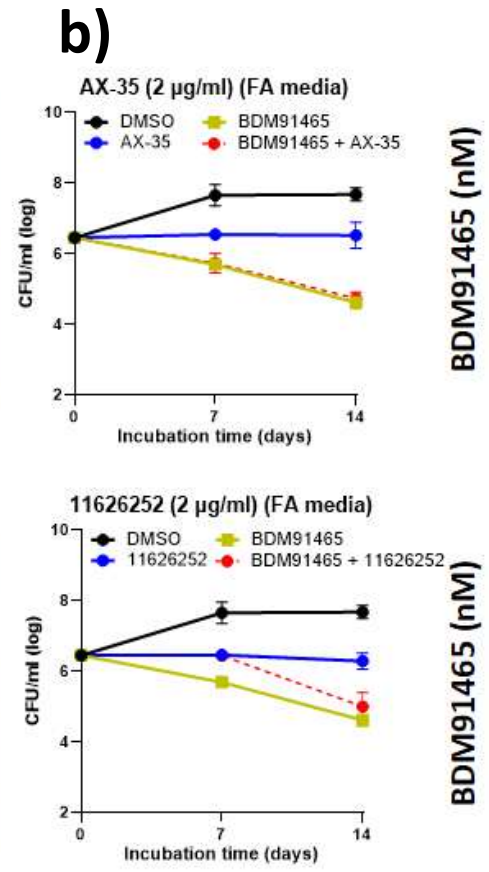

c)

AX-35 (µg/ml) vs. BDM91465 (nM)

BDM91465 (nM)	20	10	5	2.5	1.25	0.63	0.31	0.16	0
160	0	1	0	1	1	0	4	36	86
80	0	2	4	4	5	5	11	52	87
40	1	4	6	9	12	19	28	66	86
20	1	5	8	10	18	31	54	80	93
10	2	4	7	11	19	36	60	84	95
5	2	4	7	10	19	36	63	86	95
0	2	4	7	11	20	37	68	94	96

11626252 (µg/ml) vs. BDM91465 (nM)

BDM91465 (nM)	10	5	3	1.25	0.63	0.31	0.16	0.08	0
160	0	0	1	1	1	1	1	2	78
80	2	3	3	4	4	5	6	7	83
40	3	4	5	6	8	11	14	23	90
20	3	4	6	7	9	12	19	43	97
10	3	5	6	8	9	13	21	46	100
5	3	5	6	7	9	13	22	50	96
0	3	5	6	8	10	15	24	53	98

d)

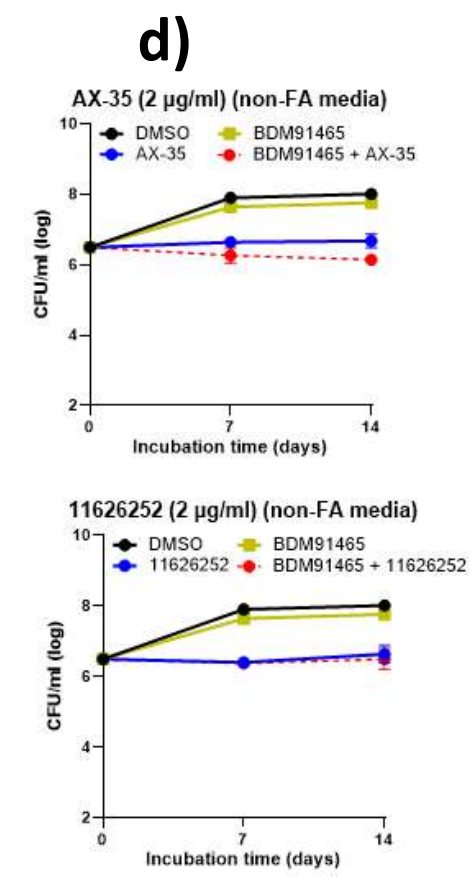

Figure S24: Synergistic activity between TriSLa BDM91465 with other mycobacterial cytochrome bc1-aa3 inhibitors against *Mtb*. (**a**, **c**) Checkerboard assays and (**b**, **d**) time kill assay data for combinations of TriSLa BDM91465 and QcrB inhibitor AX-35 (top), QcrA inhibitor 11626252 (bottom) against H37Rv-GFP or H37Rv in (**a**, **b**) $MMAT_{GluFA}$ or (**c**, **d**) $MMAT_{GluGlyChl}$. Bacterial growth was depicted as percentage of GFP fluorescence (Excitation 485 nm, Emission 510 nm) compared to no drug control (Values were background corrected). Percentage GFP is colored arbitrarily to facilitate analysis. Data represent the mean of at least 2 biological replicates. biological replicates. Time-kill kinetics (b-d) were performed with TriSLa BDM91465 (0.4 µM) in combination drugs including AX-35 (2 µg/ml) and 11626252 (2 µg/ml), for a 1-week or 2-week period. Bacterial survival was expressed in CFU/mL and values were the average with SD of at least three biological replicates.

a)

AX-35 (µg/ml) vs. BDM88689 (µM)

BDM88689 (µM)	20	10	5	2.5	1.25	0.63	0.31	0.16	0
1.6	6	4	2	2	2	1	1	1	0
0.8	6	4	5	7	6	3	3	2	4
0.4	7	7	11	17	21	13	9	6	4
0.2	10	12	17	26	49	53	49	38	41
0.1	12	13	20	31	52	77	82	83	81
0.05	9	14	22	34	51	79	91	90	91
0	11	15	22	33	53	83	92	92	93

11626252 (µg/ml) vs. BDM88689 (µM)

BDM88689 (µM)	10	5	3	1.25	0.63	0.31	0.16	0.08	0
1.6	9	6	3	3	3	3	1	1	3
0.8	9	6	6	6	6	8	4	2	4
0.4	9	7	9	11	13	18	22	14	3
0.2	11	9	10	13	15	26	43	65	43
0.1	11	11	11	14	18	28	48	77	78
0.05	12	11	11	14	19	25	47	79	88
0	10	10	10	14	18	27	45	82	90

b)

AX-35 (µg/ml) vs. BDM88689 (µM)

BDM88689 (µM)	20	10	5	2.5	1.25	0.63	0.31	0.16	0
1.6	0	1	2	2	3	4	11	67	85
0.8	1	3	5	7	10	12	23	75	89
0.4	1	3	6	9	16	33	53	81	95
0.2	1	4	7	11	20	34	60	86	98
0.1	1	4	7	11	20	37	65	89	99
0.05	2	4	7	12	19	38	66	90	96
0	1	4	6	11	20	41	65	90	99

11626252 (µg/ml) vs. BDM88689 (µM)

BDM88689 (µM)	10	5	3	1.25	0.63	0.31	0.16	0.08	0
1.6	1	2	2	2	3	3	2	4	78
0.8	3	4	5	6	7	8	12	15	84
0.4	3	4	5	6	8	11	18	36	89
0.2	2	4	5	7	8	12	20	44	95
0.1	2	4	5	7	8	13	22	47	97
0.05	3	4	5	6	8	12	20	45	97
0	2	3	5	6	8	11	20	49	98

Figure S25: Synergistic activity between TriSLa BDM88689 with other mycobacterial cytochrome bc1-aa3 inhibitors against *Mtb*. Checkerboard assays for BDM88689 in combination with QcrB inhibitor AX-35 (top), QcrA inihibitor 11626252 (bottom) against H37Rv-GFP growing in (**a**) $MMAT_{GluFA}$ and (**b**) $MMAT_{GluGlyChl}$. Bacterial growth was depicted as percentage of GFP fluorescence (Excitation 485 nm, Emission 510 nm) compared to no drug control. Percentage GFP is colored arbitrarily to facilitate analysis. Data represent the mean of at least 2 biological replicates

ROT (µg/ml)

BDM91465 (nM)	200	100	50	25	13	6	3	2	0
160	-1	0	0	0	7	61	71	64	78
80	0	0	0	15	22	77	79	81	93
40	1	2	1	12	66	71	87	86	89
20	7	9	23	52	72	87	93	99	99
10	44	63	60	82	93	96	103	98	98
5	59	70	79	94	101	103	103	102	102
0	57	70	101	101	104	98	102	113	112

BDQ (ng/ml)

BDM91465 (nM)	4000	2000	1000	500	250	125	63	31	0
160	-1	-1	0	0	3	5	8	35	71
80	3	0	0	1	7	43	57	52	70
40	1	-1	0	1	7	50	58	58	89
20	0	-1	0	2	13	64	67	83	94
10	2	0	3	3	7	53	67	81	90
5	6	1	1	2	7	62	61	81	94
0	1	2	1	4	11	65	75	85	103

CFZ (µg/ml)

BDM91465 (nM)	10	5	2.5	1.25	0.63	0.31	0.16	0.08	0
160	-4	-3	0	2	5	7	15	26	51
80	-4	0	1	2	4	12	17	24	53
40	-3	-1	3	2	9	16	28	38	76
20	-2	0	6	6	12	21	33	49	87
10	-2	3	10	12	20	23	39	56	84
5	0	6	13	19	24	31	51	60	88
0	7	16	23	26	31	46	63	82	93

Q203 (ng/ml)

BDM91465 (nM)	50	25	12.5	6.25	3.13	1.56	0.78	0.39	0
160	-3	-3	-3	-2	-2	0	6	23	49
80	-2	-2	-2	-1	-1	1	12	38	57
40	3	2	4	4	6	9	19	47	64
20	3	3	4	10	15	23	42	66	85
10	3	4	7	10	19	33	58	80	105
5	3	4	5	11	30	42	66	91	107
0	3	6	6	16	30	51	81	108	115

Figure S26. Synergistic activity of TriSLa BDM91465 with bacterial respiration inhibitors against *Mtb* in Sauton's base media. Checkerboard assays for BDM91465 in combination with rotenone (ROT), bedaquiline (BDQ), clofazimine (CFZ) and telacebec (Q203) on H37Rv-GFP growing in Sauton's base media having Glucose (0.2%) and Glycerol (0.2%) as the main carbon sources. Bacterial growth was depicted as percentage of GFP fluorescence (Excitation 485 nm, Emission 510 nm) compared to no drug control. Percentage GFP is colored arbitrarily to facilitate analysis. Data represent the mean of 2 biological replicates

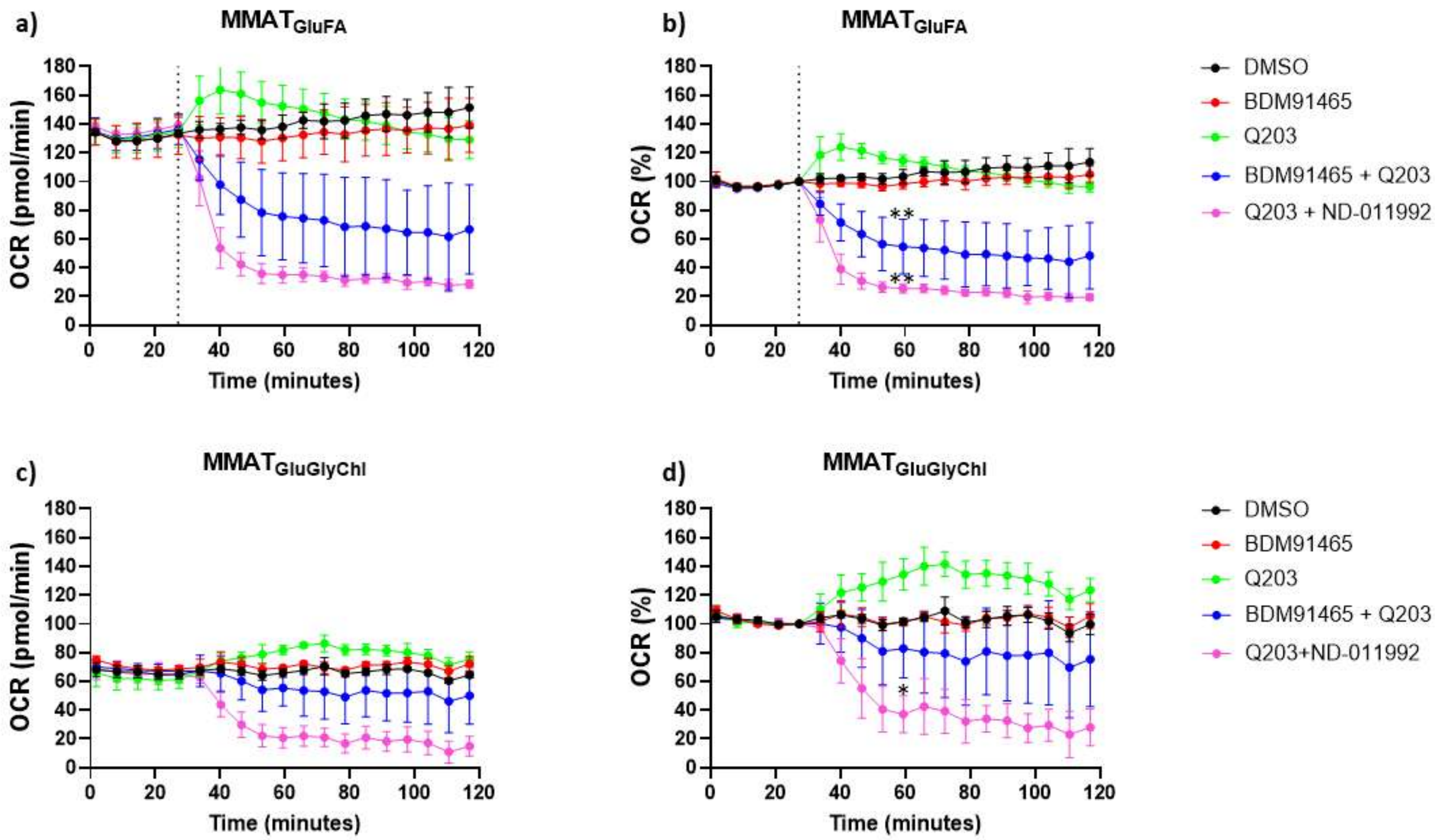

Figure S27. Effect of TriSLa and TriSLa with telacebec (Q203) combination on Oxygen Consumption Rate (OCR) of *Mtb*. Monitoring of the H37Rv oxygen consumption rate (OCR), when growing in (**a**, **b**) MMAT$_{GluFA}$ and (**c**, **d**) MMAT$_{GluGlyChl}$, in response to an injection (at 27 min) of DMSO (black circles), BDM91465 (BDM91465 at 0.6 µM)(red circles), Q203 (Telacebec at 100 nM) (green circles) (100 nM), BDM91465 (0.6 µM) + Q203 (blue circles) or Q203 + ND-011992 (12.5 µM) (pink circles). Data are shown as either (**a**, **c**) raw OCR values (pmol/min), to help appreciate the relative decrease in basal respiration of Mtb growing in MMAT$_{GluGlyChl}$ compared to MMAT$_{GluFA}$, or as percentage change in (**b, d**) OCR, representing OCR normalized to respiration occurring at 27 min. Statistical analysis was performed with Graphpad Prism v11. on data at 59 min (32 min post injection) using a non-parametric Mann-Whitney U test (*, $p<0.05$; **, $p<0.01$)

INH (ng/ml)

BDM91465 (nM)	1000	500	250	125	63	31	16	8	0
160	2	4	4	7	11	15	14	15	14
80	2	3	3	7	15	18	21	21	20
40	3	3	5	10	19	30	34	32	35
20	2	3	5	11	23	54	59	55	59
10	3	3	4	9	19	76	77	82	88
5	3	3	5	6	20	103	121	103	103
0	2	3	4	6	16	96	107	106	107

INH (ng/ml)

BDM88689 (µM)	1000	500	250	125	63	31	16	8	0
1.6	0	1	4	4	8	12	11	11	11
0.8	1	2	3	5	10	15	15	14	15
0.4	1	2	3	7	11	22	25	24	21
0.2	2	2	3	7	19	42	44	49	46
0.1	2	2	3	7	15	55	77	73	80
0.05	2	1	2	6	17	85	96	93	89
0	2	1	1	5	10	66	92	98	100

RIF (ng/ml)

BDM91465 (nM)	2000	1000	500	250	125	63	31	16	0
160	0	0	-1	1	3	5	9	10	15
80	0	0	0	1	3	7	11	16	22
40	1	0	0	0	3	10	17	23	31
20	0	1	0	2	8	17	28	38	54
10	1	0	0	2	6	21	42	53	69
5	1	1	0	4	9	34	55	72	98
0	1	0	0	4	11	28	55	74	106

RIF (ng/ml)

BDM88689 (µM)	2000	1000	500	250	125	63	31	16	0
1.6	2	2	2	2	1	3	5	6	9
0.8	-1	1	2	1	-1	2	7	9	13
0.4	-1	0	2	0	0	5	12	14	22
0.2	0	0	2	-1	2	11	23	27	45
0.1	-1	-1	-1	-1	3	19	37	47	68
0.05	-1	-1	-1	-1	2	16	43	60	91
0	0	0	-1	0	3	20	42	60	87

PBTZ169 (ng/ml)

BDM91465 (nM)	100	50	25	13	6.3	3.1	1.6	0.8	0
160	2	3	6	10	15	20	21	21	21
80	3	5	6	11	17	25	28	28	28
40	3	5	6	12	22	35	38	41	38
20	4	5	8	14	27	46	56	58	60
10	4	6	9	16	31	63	72	87	87
5	4	6	9	19	33	75	103	108	105
0	4	6	9	23	31	78	106	116	119

PBTZ169 (ng/ml)

BDM88689 (µM)	100	50	25	13	6.3	3.1	1.6	0.8	0
1.6	3	5	6	12	17	22	22	24	24
0.8	4	5	7	12	17	25	26	28	28
0.4	4	6	9	14	20	34	36	36	35
0.2	4	5	8	15	27	45	56	53	52
0.1	4	5	8	15	32	61	76	77	72
0.05	4	5	9	17	35	71	90	96	96
0	3	5	9	16	34	71	95	100	106

EMB (µg/ml)

BDM91465 (nM)	20	10	5	2.5	1.3	0.63	0.31	0.16	0
160	0	1	3	4	7	9	12	12	12
80	0	1	4	6	10	13	16	18	20
40	0	2	4	8	13	19	28	32	33
20	0	2	4	11	20	34	47	55	62
10	0	2	4	12	24	44	70	81	74
5	0	2	4	14	29	60	91	102	104
0	0	1	5	16	30	59	99	115	125

EMB (µg/ml)

BDM88689 (µM)	20	10	5	2.5	1.3	0.63	0.31	0.16	0
1.6	0	1	1	7	8	10	12	13	13
0.8	0	0	2	7	8	12	15	15	18
0.4	0	1	3	7	12	20	26	29	30
0.2	0	1	3	8	17	28	46	52	59
0.1	0	2	4	10	23	42	67	80	80
0.05	0	1	3	9	24	47	74	92	96
0	0	1	4	10	25	54	80	97	104

LNZ (µg/ml)

BDM91465 (nM)	20	10	5	2.5	1.3	0.63	0.31	0.16	0
160	0	0	1	3	6	9	10	11	12
80	0	0	2	4	8	12	13	16	18
40	0	0	1	3	8	16	21	26	31
20	0	0	2	4	8	16	29	45	55
10	1	1	1	4	8	19	36	64	76
5	1	1	1	4	8	16	35	71	125
0	1	1	2	5	9	17	31	73	123

LNZ (µg/ml)

BDM88689 (µM)	20	10	5	2.5	1.3	0.63	0.31	0.16	0
1.6	1	0	1	5	7	8	9	12	13
0.8	0	0	1	5	7	11	12	14	18
0.4	0	0	1	4	8	16	20	25	29
0.2	0	0	1	4	11	17	28	44	57
0.1	0	0	1	4	9	19	34	66	97
0.05	0	0	1	4	9	17	33	72	110
0	0	0	1	4	8	16	33	69	114

MOX (ng/ml)

BDM91465 (nM)	500	250	125	63	31	16	7.8	3.9	0
160	6	12	17	18	19	18	19	18	17
80	7	13	22	25	26	27	27	25	25
40	7	17	29	39	37	34	40	46	40
20	7	20	41	59	57	51	64	66	67
10	7	20	47	71	90	93	93	86	81
5	8	22	53	92	103	104	109	91	107
0	5	22	52	93	96	107	97	66	122

MOX (ng/ml)

BDM88689 (µM)	500	250	125	63	31	16	7.8	3.9	0
1.6	6	10	13	22	19	18	17	18	17
0.8	5	11	16	28	24	23	19	22	23
0.4	5	14	24	32	36	36	35	37	37
0.2	6	16	34	54	64	60	61	59	45
0.1	6	16	48	78	62	96	88	87	93
0.05	6	18	42	78	75	98	103	100	99
0	5	18	49	76	98	113	65	108	100

PRE (µg/ml)

BDM91465 (nM)	20	10	5.0	2.50	1.25	0.63	0.31	0.16	0
160	0	0	1	2	3	4	6	12	20
80	0	0	1	2	3	4	6	13	29
40	0	1	1	2	4	4	6	12	41
20	0	1	2	3	3	5	6	13	67
10	0	1	2	3	4	5	4	12	96
5	0	1	2	3	4	5	5	10	100
0	0	1	1	3	5	4	5	6	108

PRE (µg/ml)

BDM88689 (µM)	20	10	5.0	2.50	1.25	0.63	0.31	0.16	0
1.6	0	0	1	2	3	5	5	10	18
0.8	0	0	1	1	2	5	5	10	22
0.4	0	0	1	1	2	4	5	10	36
0.2	0	0	1	1	3	4	4	10	65
0.1	0	0	0	1	2	3	4	8	95
0.05	0	0	1	1	2	3	4	8	108
0	0	0	1	2	2	3	4	7	109

PAS (µg/ml)

BDM91465 (nM)	80	40	20	10	5	2.5	1.3	0.6	0
160	11	12	11	13	13	14	14	14	13
80	14	14	15	17	19	22	22	20	20
40	16	17	22	27	31	35	35	34	36
20	12	19	29	38	51	59	61	58	52
10	17	18	31	47	65	81	83	83	69
5	13	13	30	54	73	100	101	104	100
0	12	15	24	46	72	90	97	102	100

PAS (µg/ml)

BDM88689 (µM)	80	40	20	10	5	2.5	1.3	0.6	0
1.6	10	10	10	10	13	17	11	12	13
0.8	12	12	12	13	14	21	15	17	18
0.4	15	18	18	22	24	29	28	30	32
0.2	15	20	26	37	42	49	52	52	60
0.1	13	17	30	42	58	70	76	82	95
0.05	11	15	25	47	62	73	81	90	101
0	11	13	23	40	60	80	90	96	107

BDQ (ng/ml)

BDM91465 (nM)	1000	500	250	125	63	31	16	8	0
160	0	1	3	5	9	13	13	14	16
80	0	2	4	10	12	15	19	19	19
40	1	2	6	14	23	28	31	31	33
20	1	3	6	15	33	48	53	55	66
10	1	3	8	20	41	69	77	87	91
5	1	3	7	22	44	75	99	121	120
0	1	3	8	18	44	83	105	94	127

BDQ (ng/ml)

BDM88689 (µM)	1000	500	250	125	63	31	16	8	0
1.6	2	1	1	3	7	11	8	12	13
0.8	1	0	2	4	7	13	11	13	14
0.4	0	1	3	7	12	20	19	21	23
0.2	0	1	5	12	25	37	41	40	45
0.1	0	1	5	15	29	56	83	78	71
0.05	0	2	7	17	35	67	96	93	100
0	0	2	6	16	32	67	94	104	95

Q203 (ng/ml)

BDM91465 (nM)	250	125	63	31	16	7.8	3.9	2.0	0
160	8	8	7	7	8	9	10	10	10
80	9	9	8	9	9	11	13	15	14
40	10	10	10	10	11	13	17	21	23
20	11	11	11	12	13	16	21	29	43
10	13	12	12	13	14	17	23	33	45
5	12	13	12	15	15	20	25	31	79
0	12	14	14	19	15	19	18	29	106

Q203 (ng/ml)

BDM88689 (µM)	250	125	62.5	31	16	7.8	3.9	2.0	0
1.6	4	6	5	9	6	6	8	10	12
0.8	6	6	5	7	6	8	10	12	15
0.4	7	7	6	7	8	10	12	18	25
0.2	7	7	8	7	9	11	15	25	43
0.1	7	7	8	8	9	11	16	24	76
0.05	8	8	9	9	10	13	16	25	91
0	8	8	8	10	11	15	18	26	93

CFZ (µg/ml)

BDM91465 (nM)	10	5	2.5	1.3	0.63	0.31	0.16	0.08	0
160	0	2	5	9	20	27	32	34	33
80	0	2	2	12	22	28	36	38	41
40	2	3	4	10	25	32	40	45	52
20	2	4	7	14	29	37	43	51	73
10	0	5	23	16	26	41	61	72	94
5	7	6	15	24	30	46	69	85	106
0	4	9	22	11	29	50	65	71	115

CFZ (µg/ml)

BDM88689 (µM)	10	5	2.5	1.3	0.63	0.31	0.16	0.08	0
1.6	2	4	4	7	16	23	22	26	25
0.8	1	2	2	9	18	25	28	28	30
0.4	-1	1	4	8	21	31	36	38	42
0.2	1	2	1	10	26	37	48	56	61
0.1	0	10	11	23	25	42	61	71	90
0.05	-1	0	2	25	26	47	89	81	111
0	0	1	3	34	49	40	67	85	114

Figure S28. Efficacy of TriSLa in combination with antituberculosis drugs against intracellular *Mtb*. Checkerboard assays showing percentage GFP fluorescence from H37Rv-GFP *Mtb* within the RAW264.7 macrophages after incubation in combinations of TriSLa BDM91465 (left), or BDM88689 (right) with a panel of antibiotics including isoniazid (INH), rifampicin (RIF), macozinone (PBTZ169), ethambutol (EMB), linezolid (LNZ), moxifloxacin (MOX), pretomanid (PRE), *para*-Aminosalicylic Acid (PAS), bedaquiline (BDQ), telacebec (Q203), clofazimine (CFZ). RAW macrophages were infected with H37Rv-GFP at the MOI of 2, and incubated in the presence of drugs for 5 days. The checkerboard data shows the intracellular bacterial growth depicted as percentage of GFP fluorescence compared to no drug control. Percentage GFP is colored arbitrarily to facilitate analysis. Data represent the mean of 2 biological replicates.

References

1. K. A. I, *et al.*, New Recombinant Mycobacterium bovis BCG Expression Vectors: Improving Genetic Control over Mycobacterial Promoters. *Appl. Environ. Microbiol.* **82**, 2240–2246 (2016).

2. J. J. Baker, R. B. Abramovitch, Genetic and metabolic regulation of *Mycobacterium tuberculosis* acid growth arrest. *Sci. Rep.* **8** (2018).

3. T. Beites, *et al.*, Plasticity of the *Mycobacterium tuberculosis* respiratory chain and its impact on tuberculosis drug development. *Nat. Commun.* **10** (2019).

4. K. C. Murphy, *et al.*, Orbit: A new paradigm for genetic engineering of mycobacterial chromosomes. *mBio* **9**, 1–20 (2018).

5. A. S. Meijers, *et al.*, Efficient genome editing in pathogenic mycobacteria using *Streptococcus thermophilus* CRISPR1-Cas9. *Tuberculosis (Edinb).* **124**, 101983 (2020).

6. S. Dam, *et al.*, Tricyclic SpiroLactams Kill Mycobacteria *in vitro* and *in vivo* by Inhibiting Type II NADH Dehydrogenases. *J. Med. Chem.* **65**, 16651–16664 (2022).

7. L. Faïon, *et al.*, Design and Synthesis of Metabolically stable Tricyclic-SpiroLactams inhibitors of Mycobacterium tuberculosis type-II NADH dehydrogenase: Structure–Activity and Structure–Property Relationship Studies. [Preprint] (2026).

8. C. S. Foo, *et al.*, Arylvinylpiperazine amides, a new class of potent inhibitors targeting QcrB of *Mycobacterium tuberculosis*. *mBio* **9** (2018).

9. R. C. Hartkoorn, *et al.*, Towards a new tuberculosis drug: Pyridomycin - nature's isoniazid. *EMBO Mol. Med.* **4**, 1032–1042 (2012).

10. T. Christophe, F. Ewann, H. K. Jeon, J. Cechetto, P. Brodin, High-content imaging of *Mycobacterium tuberculosis*-infected macrophages: an *in vitro* model for tuberculosis drug discovery. *Future Med. Chem.* **2**, 1283–93 (2010).

11. H. A. Howell Wescott, D. M. Roberts, C. L. Allebach, R. Kokoczka, T. Parish, Imidazoles Induce Reactive Oxygen Species in *Mycobacterium tuberculosis* Which Is Not Associated with Cell Death. *ACS Omega* **2**, 41–51 (2017).

12. S. Ahmed, S. Chowdhury, J. Gomez, D. T. Hung, T. Parish, Benzene Amide Ether Scaffold is Active against Non-replicating and Intracellular *Mycobacterium tuberculosis*. *ACS Infect. Dis.* **9**, 1981–1992 (2023).

13. B. K. Johnson, M. B. Scholz, T. K. Teal, R. B. Abramovitch, SPARTA: Simple Program for Automated reference-based bacterial RNA-seq Transcriptome Analysis. *BMC Bioinformatics* **17**, 66 (2016).

14. B. Bosch, *et al.*, Genome-wide gene expression tuning reveals diverse vulnerabilities of *M. tuberculosis*. *Cell* **184**, 4579-4592.e24 (2021).

15. S. Li, *et al.*, CRISPRi chemical genetics and comparative genomics identify genes mediating drug potency in *Mycobacterium tuberculosis*. *Nat. Microbiol.* **7**, 766–779 (2022).

16. M. H. Larsen, K. Biermann, S. Tandberg, T. Hsu, W. R. Jacobs, Genetic Manipulation of *Mycobacterium tuberculosis*. *Curr. Protoc. Microbiol.* **Chapter 10**, Unit 10A.2 (2007).

17. M. Martin, Cutadapt removes adapter sequences from high-throughput sequencing reads. *EMBnet. J.* **17**, 10 (2011).

18. W. Li, *et al.*, MAGeCK enables robust identification of essential genes from genome-scale CRISPR/Cas9 knockout screens. *Genome Biol.* **15**, 554 (2014).

19. R. S. Jansen, *et al.*, Aspartate aminotransferase Rv3722c governs aspartate-dependent nitrogen metabolism in *Mycobacterium tuberculosis*. *Nat. Commun.* **11**, 1960 (2020).

20. R. Antoine, C. Gaudin, R. C. Hartkoorn, Intragenic Distribution of IS6110 in Clinical *Mycobacterium tuberculosis* Strains: Bioinformatic Evidence for Gene Disruption Leading to Underdiagnosed Antibiotic Resistance. *Microbiol. Spectr.* **9** (2021).

21. B. Springer, P. Sander, L. Sedlacek, K. Ellrott, E. C. Böttger, Instability and site-specific excision of integration-proficient mycobacteriophage L5 plasmids: development of stably maintained integrative vectors. *Int. J. Med. Microbiol.* **290**, 669–75 (2001).

22. K. Kolbe, *et al.*, Development and Optimization of Chromosomally-Integrated Fluorescent *Mycobacterium tuberculosis* Reporter Constructs. *Front. Microbiol.* **11**, 591866 (2020).
